# Discovery of Di(het)arylmethane and Dibenzoxanthene Derivatives as Potential Anticancer Agents

**DOI:** 10.3390/ijms25126724

**Published:** 2024-06-18

**Authors:** Andrey Smolobochkin, Dinara Niyazova, Almir Gazizov, Marat Syzdykbayev, Alexandra Voloshina, Syumbelya Amerhanova, Anna Lyubina, Margarita Neganova, Yulia Aleksandrova, Olga Babaeva, Julia Voronina, Nurbol Appazov, Oleg Sinyashin, Igor Alabugin, Alexander Burilov, Michail Pudovik

**Affiliations:** 1 Arbuzov Institute of Organic and Physical Chemistry, FRC Kazan Scientific Center, Russian Academy of Sciences, Arbuzov Str., 8, Kazan 420088, Russia; agazizov@iopc.ru (A.G.); sobaka-1968@mail.ru (A.V.); olga.babaeva@iopc.ru (O.B.);; 2 Laboratory of Engineering Profile, Department of Engineering Technology, Korkyt Ata Kyzylorda University, Ayteke bi Str., 29A, Kyzylorda 120014, Kazakhstan; din_bota.87@mail.ru (D.N.); marat.1980@mail.ru (M.S.); 3 Nazarbayev Intellectual School Chemical-Biological Direction in Kyzylorda, Sultan Beybars Str., 6, Kyzylorda 120014, Kazakhstan; 4 Institute of Physiologically Active Compounds at Federal Research Center of Problems of Chemical Physics and Medicinal Chemistry, Russian Academy of Sciences, Severnij pr., 1, Chernogolovka 142432, Russia; 5 N.S. Kurnakov Institute of General and Inorganic Chemistry, Russian Academy of Sciences, Leninskii pr., 31, Moscow 119071, Russia; 6 Limited Liability Partnership «DPS-Kyzylorda», Amangeldi Str., 112A, Kyzylorda 120014, Kazakhstan; 7 Department of Chemistry and Biochemistry, Florida State University, Chieftan Way Str., 95, Tallahassee, FL 32306-3290, USA

**Keywords:** di(het)arylmethane, dibenzoxanthene, anticancer agents, cytotoxic activity

## Abstract

A family of bifunctional dihetarylmethanes and dibenzoxanthenes is assembled via a reaction of acetals containing a 2-chloroacetamide moiety with phenols and related oxygen-containing heterocycles. These compounds demonstrated selective antitumor activity associated with the induction of cell apoptosis and inhibition of the process of glycolysis. In particular, bis(heteroaryl)methane containing two 4-hydroxy-6-methyl-2*H*-pyran-2-one moieties combine excellent in vitro antitumor efficacy with an IC_50_ of 1.7 µM in HuTu-80 human duodenal adenocarcinoma models with a high selectivity index of 73. Overall, this work highlights the therapeutic potential of dimeric compounds assembled from functionalized acetals and builds a starting point for the development of a new family of anticancer agents.

## 1. Introduction

Despite significant advances in the fight against cancer in recent decades, the demand for new anticancer medications continues to persist [1]. There are over 200 distinct types of cancer and each type necessitates tailored medications for effective treatment [2]. This need is exacerbated by the emerging resistance to cancer therapy and by the significant number of side effects associated with currently used drugs. The search continues for the ideal antitumor drugs that would selectively induce cancer cell death while minimizing the toxic effects on healthy cells in the body.

The vast majority of anticancer drugs developed in the last few decades are of natural origin [3,4]. The heterocyclic analogues of phenol derivatives of 4-hydroxy-2*H*-pyran-2-one are a well-known class of compounds found in natural molecules, with a wide range of pharmacological and biological activity [5,6,7,8,9]. The anticancer properties of substituted coumarin and phenol derivatives are especially interesting [10,11,12,13].

**Figure 1:**
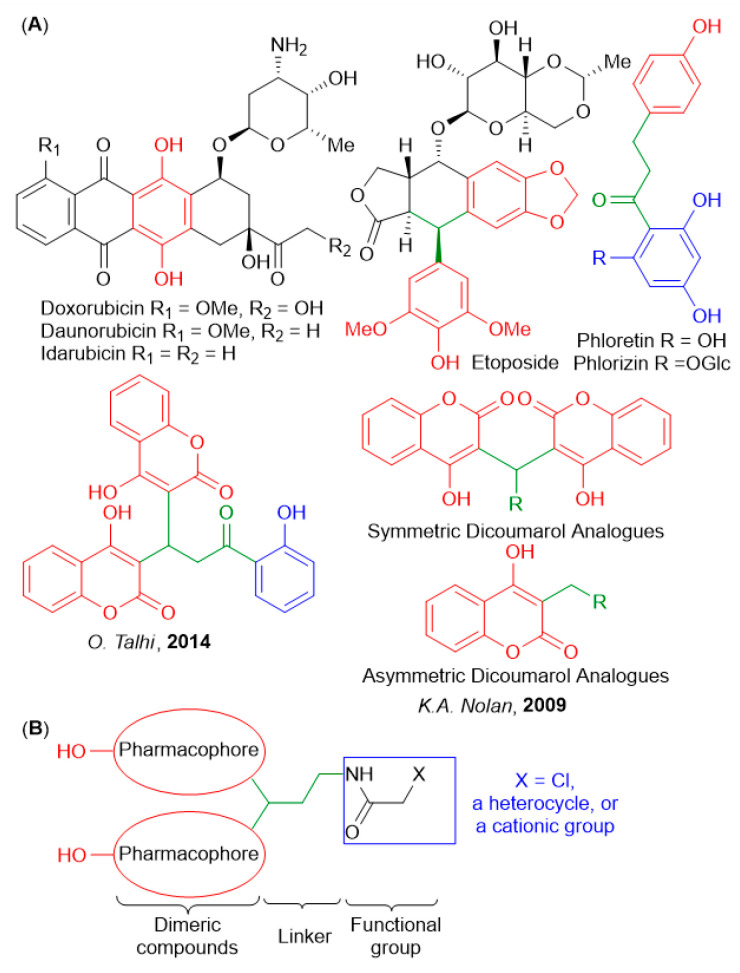
(**A**) Compounds with anticancer activity containing phenolic moieties. (**B**) Strategy for the synthesis of new dimeric compounds with anticancer activity [20,21].

In our previous studies, we found phenol derivatives that exhibit high cytotoxicity towards HuTu 80 and M-HeLa tumor cells due to the induction of apoptosis via mitochondrial pathway. An interesting feature of these compounds was a switch from redox protection to increased ROS production under the oxidative stress conditions typical for cancer cells [14,15]. Figure 1A provides selected examples of phenol- and coumarin-containing biologically active substances, including anticancer drugs. In particular, the classic anthracycline family (doxorubicin, daunorubicin, and idarubicin) of chemotherapeutic agents derived from soil bacteria features a redox-active oxygen-rich polycyclic core.

These drugs act mainly by intercalating with DNA, disrupting its function, which ultimately leads to the death of the cancer cells [16]. The quinone part of anthracyclines can undergo redox reactions with the formation of reactive oxygen species which leads to oxidative stress and DNA damage, thereby causing apoptosis [17]. On the other hand, chalcones are promising chemopreventive agents against cancer because they are effective inhibitors of histone deacetylase enzymes [18,19].

In an earlier work, Silva et al. [20] showed that biscoumarin inhibited TNFα-induced NF-jB activation in K-562 leukemia cell lines (Figure 1A) by exerting cytostatic effects at relatively low doses (IC_50_ 17.5 µM). At the same time, this compound did not affect the viability of healthy cells at concentrations exceeding 100 µM. It is very interesting that symmetric analogues of dicoumarol exhibit the greatest toxicity to pancreatic tumor cells MIA PaCa-2 and colon cancer cells HCT116, compared with nonsymmetric analogues [21].

The combination of several pharmacophores in one molecule is a promising strategy for the development of new drugs [22]. In this case, it is often possible to decrease side effects and overcome the resistance to the chemotherapeutic effects. We have recently developed a method for the synthesis of new taurine-based derivatives of diarylmethane and dibenzoxanthene, which have cytotoxic activity against human cancer cells [23].

Herein, we present the systematic SAR study with the goal to expand their potential as novel antitumor agents. As seen from Figure 1B, we chose fragments of di(het)arylmethane and dibenzoxanthene as the dimer part, and a short aliphatic chain as the linker. The presence of a 2-chloroacetamide fragment as a functional group will allow the further modification of the target molecule, offering a potentially modular approach to compounds of increasing complexity. Biological evaluation identified two leading compounds (**5a** and **6a**) with potent antitumor activity in HuTu-80 human duodenal adenocarcinoma models. These compounds combined excellent antitumor efficacy against cancer cells (1.9 and 1.7 μM doses, respectively) with low toxicity, reaching the high selectivity indexes of 73 and 24. The mechanism of action of the two leading compounds was studied in detail.

## 2. Results

### 2.1. Synthesis and Characterization of the Compounds

**Scheme 1:**
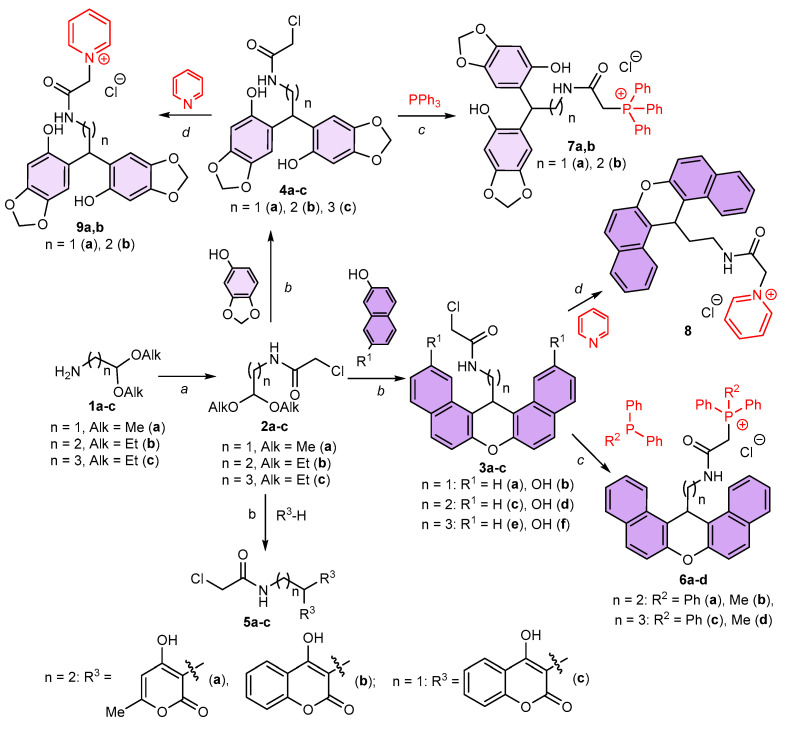
Synthetic route to di(het)arylmethane and dibenzoxanthene derivatives from bifunctional chloro acetal precursors **1a**–**c**. Cyclic substituents introduced by using the acetal moiety are shown in purple and the cationic residues introduced by the reaction of terminal carbonyl chloride are highlighted in red. Reagents and conditions: (a) ClCH_2_C(O)Cl, Et_3_N, CH_2_Cl_2_, –5–10 °C, 2 h, 62–95%; (b) CF_3_COOH, CHCl_3_, rt, 50 h, 20–76%; (c) EtOH, Δ, 72 h, 67–87%; (d) EtOH, Δ, 32 h, 49–79%.

The synthesis of the target compounds is presented in Scheme 1. Through reacting aminoacetals **1a**–**c** with 2-chloroacetyl chloride in the presence of a base, we prepared the key acetals **2a**–**c** with different lengths of the methylene spacer. The advantage of acetals for the preparation of dimeric structures is their bifunctional character as they can react with two equivalents of activated aromatic compounds via Friedel–Crafts-like alkylations with the acetal carbon. Indeed, in the presence of trifluoroacetic acid, the functionalized acetals **2** react with naphthols in chloroform to yield the series of xanthenes **3a**–**f**. Replacing naphthols with sesamol, 4-hydroxy-6-methyl-2*H*-pyran-2-one and 4-hydroxy-2*H*-chromen-2-one allowed us to prepare two additional new families of di(het)arylmethane derivatives **4a**–**c** and **5a**–**c**, respectively.

Positively charged triarylphosphonium groups are known to interact favorably with DNA through both intercalation and electrostatic attraction to negatively charged DNA backbones [24]. Similarly, the introduction of a pyridinium fragment [25] or an ammonium group [26,27] in a molecule may also enhance its DNA affinity. Thus, we speculated that the introduction of cationic moieties into a dibenzoxanthene core may have a synergistic effect which can be further modulated by the variations in the linker length. By reacting dibenzoxanthene **3c**–**f** and dihetarylmethane **4a**,**b** derivatives with substituted phosphines in boiling ethanol, phosphonium salts **6a**–**d**, **7a**,**b** were obtained. Under similar conditions, the interaction of dibenzoxanthene **3e** and diarylmethane derivatives **4a**,**b** with pyridine provided pyridinium salts **8**, **9a**,**b** in good yields. 

**Figure 2:**
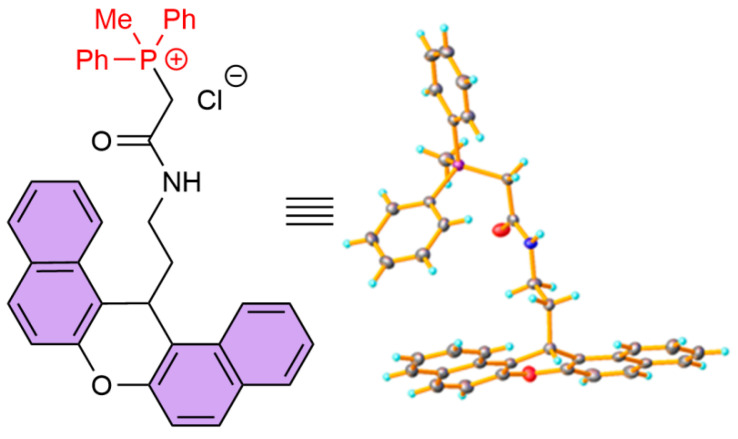
Molecular structure of investigated compound **6b**. Ellipsoids are given with a 50% probability.

Compounds **6a** and **6b** can form crystalline salts where a chloride counterion interacts with the protonated center via a hydrogen bond (Supplementary Materials). In addition, the crystal of compound **6b** is a solvate with ethanol, in a ratio of 1:1. The ethanol molecule also forms a hydrogen bond with a chloride ion (Supplementary Materials). The crystal structures of compounds **6a** and **6b** illustrate the combination of a bulky polycyclic fragment with a relatively long tether containing a urea group and ending with triphenylphosphanyl (**6a**) and methyl(diphenyl)phosphanyl (**6b**) groups. Polycyclic fragments of both molecules have a non-planar geometry. Depending on the structure, the phosphonyl fragments can participate in additional supramolecular interactions. This is seen in compound **6a**, as it were, inside the polycycle, stabilizing via CH-π interaction involving one of the phenyl rings, and in compound **6b** from the polycycle, being stabilized only by weak intermolecular interactions (Figure 2). 

**Scheme 2:**
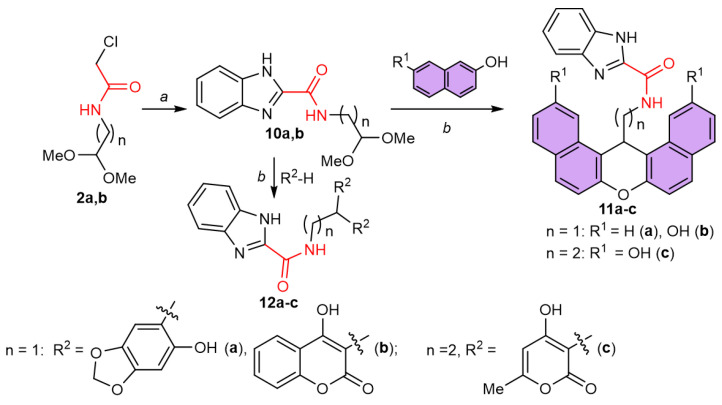
Selective activation of α-chloroacetamide moiety followed by double alkylation with the acetal group for the preparation of benzimidazole hybrids **10a**,**b**, **11a**–**c**, **12a**–**c**. Reagents and conditions: (a) benzene-1,2-diamine, DMF, Et_3_N, S_8_, 40–50 °C, 10 h, 45–63%; (b) CF_3_COOH, CHCl_3_, rt, 96 h, 38–75%.

An interesting chemoselective activation of the α-chloroacetamide moiety without the involvement of the acetal group is shown in Scheme 2. In the presence of sulfur and triethylamine, chloroacetamides **2a**,**b** react with 1,2-diaminobenzene to assemble a benzimidazole ring of compounds **10a**,**b** [28]. Acetals **10a**,**b** react with 2-naphthol and 2,7-naphthalenediol to form dibenzoxanthenes **11a**–**c**. The use of sesamol, 4-coumarin and 4-hydroxy-6-methyl-2*H*-pyran-2-one in this reaction leads to new benzimidazole derivatives **12a**–**c**.

### 2.2. Structure−Activity Relationships

The study of cytotoxic effects was an important stage in the new potential drug development. We evaluated the cytotoxicity of new compounds against both cancer and normal cell lines. Table 1 summarizes the IC_50_ values (the concentration of the test compound that causes the death of 50% of cells in the experimental population) for the new molecules. 

The new compounds show high and moderate activity against cancer lines of various origins while demonstrating moderate and low cytotoxicity against normal liver cells.

During the studies, the two leading compounds **5a** and **6a** were identified. The cytotoxic effect of **6a** against human cervical carcinoma (M-HeLa) and human duodenal adenocarcinoma (HuTu 80) cell lines was tested at concentrations of 11 µM and 1.7 µM, respectively. In both cancer lines, compound **6a** was three times more effective than the reference drug sorafenib. Compound **5a** demonstrated a high cytotoxic effect only against the HuTu 80 line at a concentration of 2.9 µM where it was 1.7 times more cytotoxic than sorafenib.

The selectivity of compounds against cancer cells is an important criterion when assessing the cytotoxic effects. The selectivity index (SI) is defined as the ratio between the IC_50_ value for normal cells and the IC_50_ value for cancer cells. The selectivity index values for the most active compounds are shown in Table 1. It can be seen that compounds **5a** and **6a** showed the highest selectivity for the human duodenal adenocarcinoma cell line (HuTu 80). Their SI values were 24 and 73, respectively. Compounds with SI ≥ 10 are generally considered highly selective [29]. According to these data, the leading compounds **5a** and **6a** exhibit high selectivity towards the human duodenal adenocarcinoma cell line HuTu 80. At the same time, the reference drugs sorafenib and doxorubicin were significantly inferior to these compounds in selectivity.

**Figure 3:**
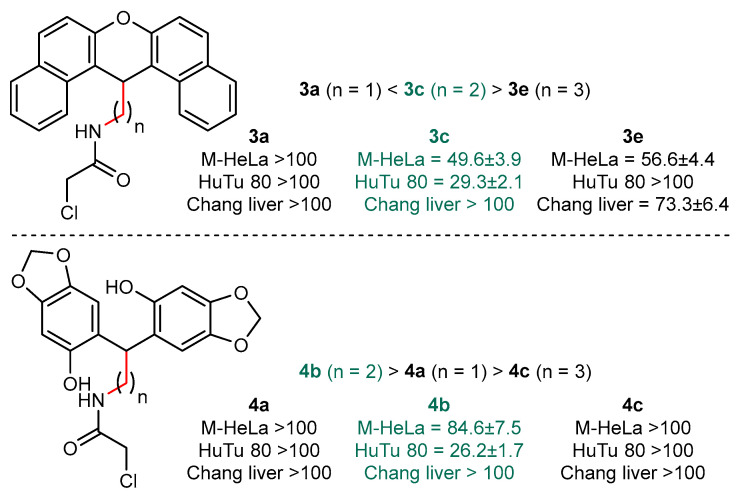
The influence of spacer length on activity by the example of compounds **3a**,**c**,**e**, **4a**–**c**.

The data on the cytotoxicity of dibenzoxanthene and dihetarylmethane derivatives against tumor and normal cell lines allow us to draw some conclusions about the influence of the structure of these compounds on their activity and selectivity of action. The analysis of these data indicates that the dibenzoxanthenes derivatives containing a methylene linker have the greatest activity. As evident from the comparison of compounds **3a**, **3c**, and **3e** (Figure 3), increasing the length of the polymethylene chain to three units results in a marked decrease in cytotoxicity. Notably, in the case of compound **3a**, this reduction is so significant that we observe no toxicity for the used concentrations. It is curious that this decrease is most clearly expressed for the tumor cell line HuTu 80. A similar picture is observed in the series of diarylmethane derivatives **4a**–**c**, which contain sesamol fragments. Thus, a spacer containing two methylene units should be considered optimal from the point of view of the cytotoxicity of the compounds under study.

**Figure 4:**
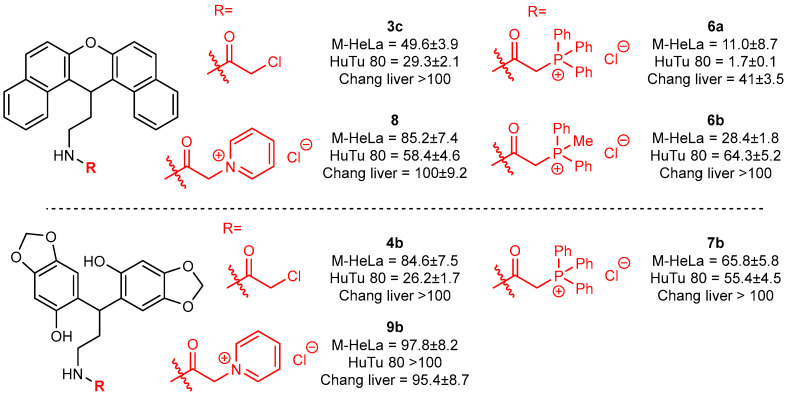
The effect of substituents in α-position on the activity of compounds **3c**, **4b**, **6a**,**b**, **7b**, **8**, **9b**.

The analysis of the effect of the nature of the nitrogen atom substituent on the cytotoxicity of dibenzoxanthene derivatives indicates that compounds **3c**, **6a** and **4b**, which contain chloroacetamide and triphenylphosphonium fragments, have the greatest activity against tumor lines (Figure 4). Replacing these substituents with both an alkylpyridinium salt and a methyldiphenylphosphonium fragment leads to a decrease in the activity of the compounds. This pattern is valid both in the series of dibenzoxanthenes and in the case of diarylmethane derivatives. It should be noted, however, that in the case of dibenzoxanthenes **3c** and **6a**, replacing the chloroacetamide fragment with a triphenylphosphonium fragment leads to an increase in cytotoxicity. We emphasize that cytotoxicity increases both in relation to tumor and normal cells, i.e., a decrease in the selectivity of action is observed at the same time. In the case of diarylmethane derivatives **4b** and **7b**, cytotoxicity towards the HuTu 80 cell line does not increase, but rather decreases.

**Figure 5:**
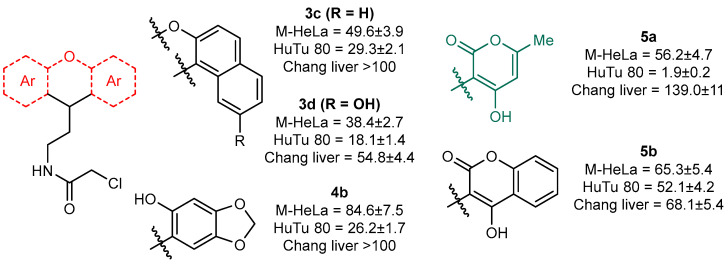
The effect of hetarylic substituents on the activity of compounds **3c**,**d**, **4b**, **5a**,**b**.

Finally, the analysis of the effect of the nature of the aryl/heteroaryl fragment on cytotoxicity against tumor cell lines allows us to conclude that the dibenzoxanthene **3d**, which features an additional hydroxyl group in the aromatic ring, and **5a**, comprising two fragments of 4-hydroxy-pyran-2-one, exhibit the highest activity. However, compound **5a** is significantly less toxic to the normal Chang liver cell line compared to compound **3d**. Conversely, compound **4b**, containing two phenolic fragments, displays the lowest activity (Figure 5).

Based on this research, several conclusions can be drawn. The length of the spacer between the aromatic/heteroaromatic fragment and the functional group exerts the greatest influence on the cytotoxicity of the resulting compounds; the optimal spacer is the one containing two methylene groups. The most active are compounds containing chloroacetamide and triphenylphosphonium fragments, with the chloroacetamide fragment providing the greatest selectivity. Compounds containing either 2,7-naphthaliniol or 4-hydroxypyran-2-one fragment display the greatest cytotoxicity towards the HuTu 80 cell line.

### 2.3. Antitumor Mechanism

**Figure 6:**
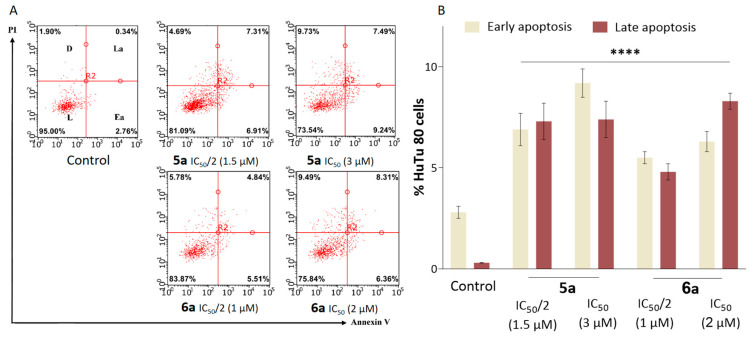
(**A**)—Induction of apoptosis in HuTu 80 cells incubated with lead compounds. 1. **5a** at concentration IC_50_/2 (1.5 µM); 2. **5a** at concentration IC_50_ (3 µM); 3. **6a** at concentration IC_50_/2 (1 µM); 4. **6a** at concentration IC_50_ (2 µM). L—living cells; D—dead cells; Ea.—early apoptotic cells; La.—late apoptotic cells. (**B**)—Representative histograms for the number of cells (% of total) in the early and late stages of apoptosis for the control and treatment groups. The values are presented as the mean ± SD; ****—*p* < 0.0001 versus control. The statistical analysis was performed using two-way ANOVA and the Bonferroni test.

The ability of the new compounds, proposed as antitumor agents, to induce cell death via apoptosis is a critical feature for their potential use in therapy. The apoptosis-inducing effects of lead compounds were investigated using flow cytometry at IC_50_/2 and IC_50_ concentrations on the HuTu 80 cell line (Figure 6A) [30]. After 24 h incubation of HuTu 80 cells in the presence of compound **5a** at a concentration of IC_50_/2, cells in both early and late stages of apoptosis were observed to be approximately the same number. As the concentration increases to the IC_50_ value, the apoptotic effects increase at the early apoptosis stage.

In contrast to **5a**, dose-dependent apoptosis was observed for compound **6a** in duodenal adenocarcinoma cells, and the apoptotic effects were predominant at the late stage (Figure 6B). The results stem from the differences in the structure of the leading compounds **6a** and **5a**.

Early stage apoptotic effects are usually accompanied by cell shrinkage and the loss of up to one-third of its volume within a few minutes. Following this, one of the two main mechanisms of apoptosis is activated: an external one through death receptors, or an internal one mediated by mitochondria. The first pathway triggers apoptosis in response to external stimuli, e.g., the binding of specific ligands to death receptors on the surface of the cell membrane. In mitochondrial apoptosis, cell death results from irreparable DNA damage which triggers an internal apoptotic cascade. Such internal induction of apoptosis is accompanied by the destruction of the mitochondrial membrane. This destruction decreases in the membrane potential, a key indicator of cell state.

The membrane potential of mitochondria can be studied using flow cytometry with the help of cationic lipophilic dyes. Such dyes are commonly referred to as “mitochondrial probes” in the literature. These dyes can serve as lipophilic probes capable of penetrating the bilipid membranes (the surface membrane of the cell, as well as the outer and inner membranes of mitochondria) and accumulating in areas with a high concentration of protons such as the inner membrane of mitochondria. This effect is accompanied by a change in the fluorescence intensity of the cells, which is registered using a flow cytometer [31].

**Figure 7:**
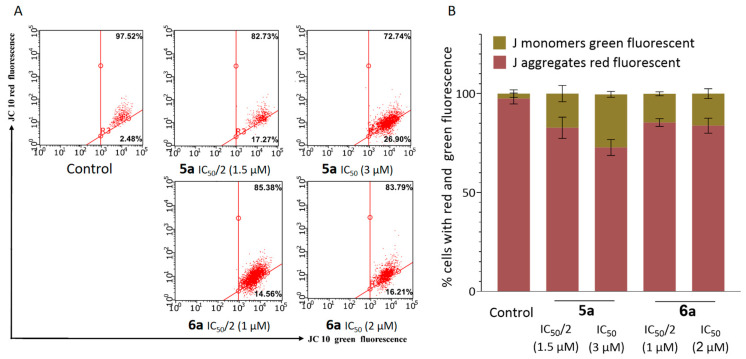
(**A**)—Effect of lead compounds on the mitochondrial membrane potential in HuTu 80 cells. 1. **5a** at concentration IC_50_/2 (1.5 µM); 2. **5a** at concentration IC_50_ (3 µM); 3. **6a** at concentration IC_50_/2 (1 µM); 4. **6a** at concentration IC_50_ (2 µM). (**B**)—Quantitative determination of portion of HuTu 80 cells (%) with red and green aggregates. Data are presented as ± SD (n = 3).

In this study, we used the fluorescent dye JC-10 from the Mitochondria Membrane Potential Kit (Sigma, St. Louis, MO, USA). Changes in mitochondrial membrane potential under the influence of lead compounds **5a** and **6a** were determined at IC_50_/2 and IC_50_ concentrations on the HuTu 80 cell line. When JC-10 accumulates in the mitochondrial matrix, it forms fluorescent J-aggregates. Their red fluorescence is characteristic for normal cells with high mitochondrial membrane potential. The reduction in mitochondrial membrane potential in apoptotic cells allows JC-10 to escape out of the mitochondria in a monomeric form which has green fluorescence. The mitochondrial membrane potential of HuTu 80 cells is decreased after 24 h treatment with the lead compounds **5a** and **6a**. The change becomes more pronounced as the concentrations of the tested compounds approach IC_50_ (Figure 7). These results indicate that the mechanism of cytotoxic action of **5a** and **6a** is likely to be related to the induction of apoptosis through the internal mitochondrial pathway.

**Figure 8:**
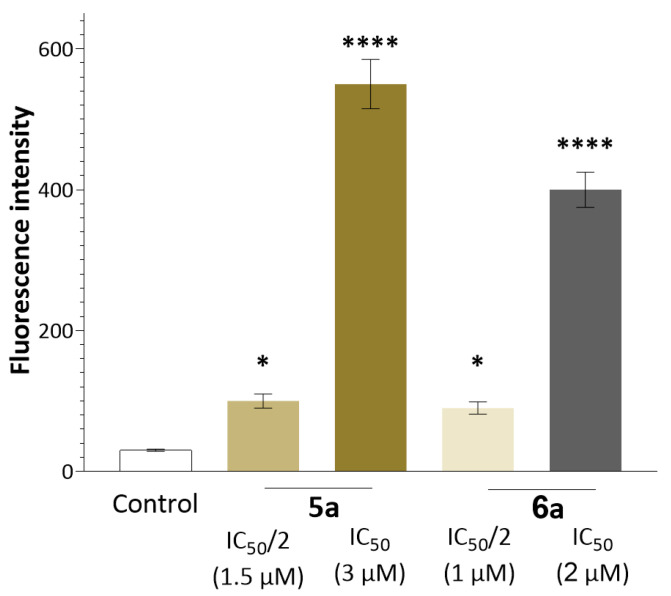
Induction of ROS production by lead compounds. **5a** at concentration IC_50_/2 (1.5 µM) and IC_50_ (3 µM); **6a** at concentration IC_50_/2 (1 µM) and IC_50_ (2 µM). Data are presented as mean ± SD of three independent experiments. * and ****, *p* < 0.05 and *p* < 0.0001, vs. control (one-way ANOVA, Dunnett’s multiple comparison tests).

The apoptosis via the mitochondrial pathway also increases the production of reactive oxygen species (ROS). As mitochondria itself becomes the target of ROS, an increase in their production disrupts mitochondrial functions and culminates in irreversible cell damage. Therefore, the effect of lead compounds **6a** and **5a** at IC_50_/2 and IC_50_ concentrations on ROS production in HuTu 80 cells was evaluated using flow cytometry. The data presented in Figure 8 demonstrate a significant reliable increase in CellROX^®^ Deep Red fluorescence intensity in the presence of HuTu 80 cells (Thermo Fisher, Waltham, MA, USA). Such an effect is not observed for intact cells in the presence of both compounds, indicating an increase in ROS production in cancer tissues.

### 2.4. Suppression of Proliferation and Glycolysis of Human Ovarian Teratocarcinoma Cells PA-1

In addition to the already studied effect (of lead compounds **5a** and **6a**) on the tumor cell survival of M-HeLa and HuTu 80, we carried out an extended analysis of the cytotoxic profile using cells of different origins. The compound **6a** was found to be the most toxic in this panel of tumor cells, with an IC_50_ of cytotoxicity starting at 2.34 ± 0.01 μM (Table 2).

Under normal conditions, the energy balance in cells primarily relies on oxidative phosphorylation occurring within the mitochondria. However, during tumorigenesis, a metabolic shift occurs. Even when oxygen is abundant, cancer cells begin to favor glycolysis over mitochondrial respiration. This metabolic preference, known as aerobic glycolysis, plays an important role in the development of cancers [32]. In particular, the Warburg Effect describes the tendency of cancer cells to preferentially convert glucose into lactate in order to support the anabolic processes associated with rapid growth and uncontrolled proliferation. Thus, metabolic therapies aimed at suppressing glycolytic function hold promise for treating cancers where ineffective standard treatment leads to a very poor prognosis [33].

To investigate whether the cytotoxicity of **5a** and **6a** is related to their effects on metabolism, we quantified their inhibition of glycolysis in human ovarian teratocarcinoma PA-1 cells using Seahorse technology, which allows the rapid assessment of glycolytic metabolism in cells using a proton efflux rate assay (ECAR) [34]. It is well known that glucose in cells is converted to pyruvate and then either to lactate in the cytoplasm or CO_2_ and water in the mitochondria. As glucose is converted to lactate, protons are released into the extracellular environment to maintain intracellular pH homeostasis [35]. This extracellular acidification is measured in real time and provides insight into the rate of glycolytic proton efflux. PA-1 cells are used for the study of glycolysis since a pronounced deregulation of cellular energy is observed in the case of ovarian cancer, mediating the growth, invasion and migration of tumor cells [36,37].

**Figure 9:**
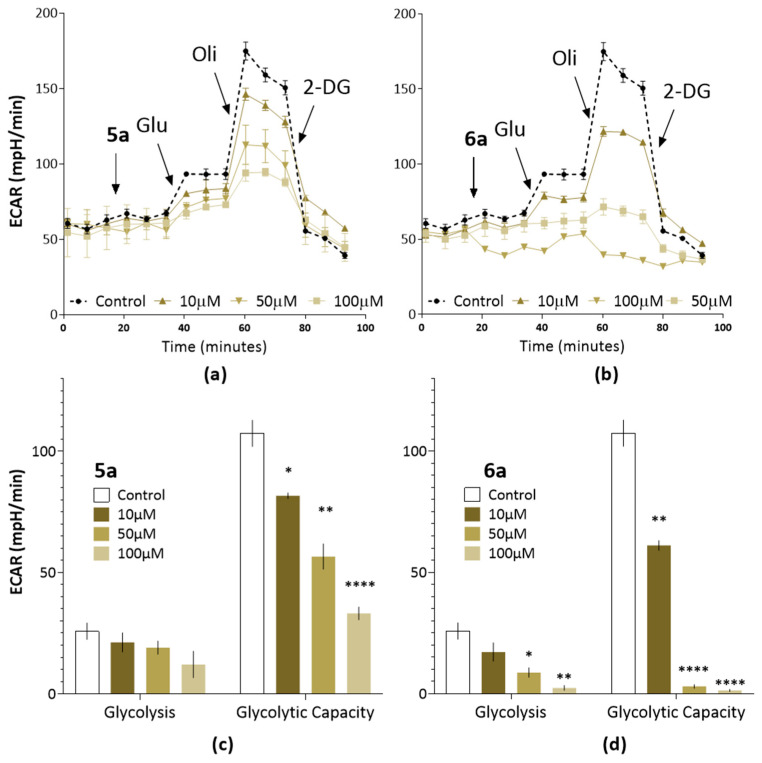
The assessment of the effect of **5a** and **6a** on the glycolytic profile of PA-1 tumor cells by measuring the rate of medium extracellular acidification (ECAR). (**a**,**b**)—Kinetic curves. (**c**,**d**)—Calculated parameters of glycolytic function (glycolysis level and glycolytic capacity). Data are presented as means ± SEM. *, ** and ****, *p* < 0.05; *p* < 0.01 and *p* < 0.0001, vs. control (one-way ANOVA, Dunnett’s multiple comparison tests).

As shown in Figure 9a,b, during the assay, we sequentially administered test compounds at different concentrations, followed by saturating with glucose to evaluate glycolysis, oligomycin to measure maximal glycolytic capacity, and 2-deoxy-D-glucose (2-DG) to inhibit the glycolysis through competitive binding of glucose hexokinase. Measuring ECAR under these conditions provides the parameters presented in Figure 9c,d: the level of glycolysis and glycolytic capacity.

It was found that the treatment of PA-1 line cells with the investigated compounds resulted in inhibited glycolysis intensity. This inhibition was evident through a decrease in extracellular acidification rate (ECAR), and this effect exhibited clear concentration dependence. Notably, compound **6a** demonstrated the greatest impact, effectively reducing extracellular acidification and completely blocking glycolysis at the maximum concentration of 100 μM (Figure 9b). Moreover, both compounds significantly reduced all calculated indicators presented in Figure 9c,d.

Importantly, compound **6a** exhibited the most pronounced ability to induce a metabolic crisis in tumor cells. This aligns well with its greatest cytotoxic activity, as observed during the analysis of cytotoxic status**.**

## 3. Materials and Methods

The ^1^H and ^13^C NMR spectra were recorded on a Bruker Avance 600 spectrometer (Billerica, MA, USA) (operating frequency 600 MHz and 150 MHz, respectively) with respect to the residual proton signals of deuterated solvents (DMSO-*d*_6_, CD_3_OD, CDCl_3_). The IR spectra were recorded on a Vector 22 Fourier spectrometer by Bruker in the range of 400–4000 cm^−1^ using KBr pellets. The elemental analysis was carried out on a CHNS analyzer Vario Macro cube (Elementar Analysensysteme GmbH, Langenselbold, Germany). The samples were weighed on Sartorius Cubis II (Göttingen, Germany) microbalance in tin capsules. VarioMacro Software V4.0.11 was used to perform quantitative measurements and evaluate the data received. The halogen content was determined using the Schöniger method. The melting points were determined in glass capillaries on a Stuart SMP 10 instrument (Sigma).

Electrospray ionization measurements were performed using UHR-QTOF Impact II mass spectrometer with Elute UHPLC system (Bruker Daltonik GmbH, Ettlingen, Germany). The column YMC-Triart C18 (50 × 2.0 mm; 3 μm) was used. The column thermostat temperature was set at 40 °C and the autosampler temperature at 12 °C. Elution solvents used Milli-Q water + 0.1% formic acid (A) and HPLC-grade methanol + 0.015% ammonium acetate (B), and the elution gradient was the following: 0 min at 5% B, 3 min at 95% B, 6 min at 95% B, 6.1 min at 5% B, and 8 min at 5% B, with a flow rate of 0.3 mL/min. The injection volume was 2 μL. Measurements were made in positive mode in the range *m*/*z* 50–1900. The ESI source conditions were as follows: capillary voltage 4500 V, desolvation temperature 220 °C, drying gas (N_2_) at flow rate of 6 L/min. The samples were prepared in HPLC-grade methanol with a concentration of 0.002 mg/mL. The solution of sodium iodide in Milli-Q water (0.2 mg/mL) was used as a calibrant. The relative error in determining the exact mass values was no more than 5 ppm. The *m*/*z* values of monoisotopic ions in the ion cluster are given in the description.

The Hystar (Bruker Daltonik GmbH, version 6.0) and the otofControl (Bruker Daltonik GmbH, version 5.2) programs were used to control the chromatograph and mass spectrometer. Data processing was performed using DataAnalysis software (Bruker Daltonik GmbH, version 5.3).

The X-ray diffraction data for the crystals of **6a**,**6b** were collected on a Bruker D8 Venture diffractometer equipped with a CCD detector (Mo-Kα, λ = 0.71073 Å, graphite monochromator). Semi-empirical absorption correction was applied using the SADABS program [38]. The structures were resolved via direct methods and refined using the full-matrix least squares in the anisotropic approximation for non-hydrogen atoms. The calculations were carried out using the SHELX-2014 program package [39] using Olex2 1.2 [40]. The crystallographic parameters for **6a**,**6b** and the structure refinement details are given in Supplementary Materials. Crystallographic data for structures reported in this paper have been deposited with the Cambridge Crystallographic Data Center (2296793, 2296794).

General Experimental Procedure for the Synthesis of **2a**–**c**. 2-chloroacetyl chloride (2.9 g, 26 mmol, 1 equiv) was added to a solution of acetal **1** (1.64 mmol, 1 equiv) in dry CH_2_Cl_2_ (20 mL) at a temperature of 5–10 °C for 2 h. The solvent was removed under reduced pressure. The residue was washed with 20 mL of benzene and dried in vacuo (10 torr, 4 h, 20 °C).

General Experimental Procedure for the Synthesis of **3**,**4**,**5**. Acetal **2** (2 mmol, 1 equiv) was added to a solution of phenol (4 mmol, 2 equiv) and trifluoroacetic acid (0.15 mL, 2 mmol, 1 equiv) in dry chloroform (10 mL). The reaction mixture was stirred at room temperature for 50 h. The solvent was removed under reduced pressure. The residue was washed with 10 mL of diethyl ether and dried in vacuo (10 torr, 10 h, 20 °C).

General Experimental Procedure for the Synthesis of **6**, **7**, **8**, **9**. Compound **3** or **4** (0.75 mmol, 1 equiv) was added to a solution of phosphine or pyridine (0.75 mmol, 1 equiv) in dry ethanol (10 mL). The reaction mixture was boiled for 72 h. The solvent was removed under reduced pressure. The residue was washed with 20 mL of benzene and dried in vacuo (10 torr, 10 h, 20 °C).

General Experimental Procedure for the Synthesis of **10**. Acetal **2** (1.1 g, 6 mmol, 1 equiv) was added to a solution of benzene-1,2-diamine (0.65 g, 6 mmol, 1 equiv) and sulfur (0.84 g) in dry DMF (6 mL) at a temperature of 40–50 °C for 10 h. The solvent was removed under reduced pressure. The residue was washed with 20 mL of ethanol dried in vacuo (10 torr, 4 h, 20 °C).

General Experimental Procedure for the Synthesis of **11**, **12**. Acetal **10** (2 mmol, 1 equiv) was added to a solution of phenol (4 mmol, 2 equiv) and trifluoroacetic acid (1 mL) in dry chloroform (10 mL). The reaction mixture was stirred at room temperature for 96 h. The solvent was removed under reduced pressure. The residue was washed with 10 mL of diethyl ether and dried in vacuo (10 torr, 10 h, 20 °C).

Cells and Materials. For the experiments, we used tumor cell cultures M-HeLa clone 11 (epithelioid carcinoma of the cervix, subline HeLa, clone M-HeLa), HuTu 80, human duodenal adenocarcinoma, Hep-2, human epithelial cells, SH-SY5Y, human neuroblast-like cells, A549, human lung adenocarcinoma, PA-1, human ovarian teratocarcinoma and MCF-7, a human mammary ductal adenocarcinoma from the collection of the Institute of Cytology, Russian Academy of Sciences (St. Petersburg, Russia); human liver cells (Chang liver) were from the collection and the Research Institute of Virology of the Russian Academy of Medical Sciences (Moscow, Russia).

MTT Assay. The cytotoxic effect on cells was determined using the colorimetric method of cell proliferation—the MTT test. NADP-H-dependent cellular oxidoreductase enzymes can, under certain conditions, reflect the number of viable cells. These enzymes reduce the tetrazolium dye (MTT) 3-(4,5-dimethylthiazol-2-yl)-2,5-diphenyl-tetrazolium bromide to insoluble blue-violet formazan, which crystallizes inside the cell. The amount of formed formazan is proportional to the number of cells with active metabolism. Cells were seeded on a 96-well Nunc plate at a concentration of 5 × 10^3^ cells per well in a volume of 100 μL of medium and cultured in a CO_2_ incubator at 37 °C until a monolayer was formed. After the nutrient medium was removed, 100 µL of the test drug solutions at the given dilutions were added to the wells, which were prepared directly in the nutrient medium with the addition of 5% DMSO to improve solubility. After 48 h of incubation of the cells with the tested compounds, the nutrient medium was removed from the plates and 100 µL of the nutrient medium without serum with MTT at a concentration of 0.5 mg/mL was added and incubated for 4 h at 37 °C. Formazan crystals were added 100 µL of DMSO to each well. Optical density was recorded at 540 nm on an Invitrologic microplate reader (Novosibirsk, Russia). The experiments for all compounds were repeated three times.

**Induction of apoptotic effects via test compounds.**

**Flow Cytometry Assay.**

Cell Culture. HuTu 80 cells at 1 × 10^6^ cells/well in a final volume of 2 mL were seeded into six-well plates. After 48 h of incubation, various concentrations of lead compounds were added to wells.

Cell Apoptosis Analysis. The cells were harvested at 2000 rpm for 5 min and were then washed twice with ice-cold PBS, and resuspended in binding buffer. Next, the samples were incubated with 5 μL of annexin V-Alexa Fluor 647 (Sigma-Aldrich, St Louis, MO, USA) and 5 μL of propidium iodide for 15 min at room temperature in the dark. Finally, the cells were analyzed using flow cytometry (Guava easy Cyte, MERCK, Rahway, NJ, USA) within 1 h. The experiments were repeated three times.

Mitochondrial Membrane Potential. Cells were harvested at 2000 rpm for 5 min and then washed twice with ice-cold PBS, followed by resuspension in JC-10 (10 µg/mL) and incubation at 37 °C for 10 min. After the cells were rinsed three times and suspended in PBS, the JC-10 fluorescence was observed using flow cytometry (Guava easy Cyte, MERCK, Rahway, NJ, USA).

Detection of Intracellular ROS. HuTU 80 cells were incubated with lead compounds at concentrations of IC_50_/2 and IC_50_ for 48 h. ROS generation was investigated using flow cytometry assay and CellROX^®^ Deep Red flow cytometry kit. For this purpose, M-HeLa cells were harvested at 2000 rpm for 5 min and then washed twice with ice-cold PBS, followed by resuspension in 0.1 mL of medium without FBS, to which was added 0.2 μL of CellROX^®^ Deep Red, which was followed by incubation at 37 °C for 30 min. The cells were washed three times and suspended in PBS. After that, the production of ROS in the cells was immediately monitored using flow cytometer (Guava easy Cyte, MERCK, Rahway, NJ, USA).

Determination of cell glycolysis. Cell glycolysis was measured with Seahorse XF Glycolysis Stress Test Kit and Seahorse Bioscience XF96 Extracellular Flux Analyzer (Agilent Technology, Boston, MA, USA). A total of 40,000 cells per well were seeded in 96-well cell culture XF microplates (Agilent Technology, Boston, MA, USA) and incubated for 24 h. ECAR levels were examined and analyzed using Seahorse Bioscience XF96 Extracellular Flux Analyzer. Cells were treated with XF basal medium (pH 7.4) containing 1 mM glutamine after sequential addition of glucose (10 mM), oligomycin (1 μM), and 2-DG (50 mM). The drugs were sequentially added into wells of XF microplates as indicated.

Statistical analysis. The IC_50_ values were calculated using the MLA–Quest Graph™ IC_50_ Calculator (AAT Bioquest, Inc., Sunnyvale, CA, USA), 14 February 2022. Statistical analysis was performed with the Mann–Whitney test (*p* < 0.05). Tabular and graphical data contain averages and standard error.

## 4. Conclusions

A library of dibenzoxanthene and dihetarylmethane derivatives was synthesized and their in vitro cytotoxicity towards cancer and normal human cell lines was studied. It was found that the optimal spacer between the (het)aromatic fragment and the functional group consists of two methylene groups. Compounds containing chloroacetamide and triphenylphosphonium fragments showed the greatest activity. Furthermore, the increase in cytotoxicity towards the HuTu 80 cell line is facilitated by fragments of either 2,7-naphthaliniol or 4-hydroxypyran-2-one. Notably, dihetarylmethane **5a** and dibenzoxanthene **6a** emerged as the most promising compounds.

Our results suggest that the mechanism of cytotoxic action for leading compounds **5a** and **6a** may be associated with the internal mitochondrial pathway for the induction of apoptosis. Additionally, these compounds inhibit the key pathway for energy production by tumor cells—glycolysis. The observed patterns will be used for the future design of compounds with high antitumor activity and selectivity, which will be reported in the due course.

## Figures and Tables

**Table 1:** Cytotoxic Effects (IC_50_, µM) of test compounds.

Test Compounds	IC_50_ (µM)		
	Cancer Cell Lines		Normal Cell Line
	M-HeLa	HuTu 80	Chang Liver
**2a**	>100	>100	>100
**2b**	>100	>100	>100
**3a**	>100	>100	>100
**3b**	96.4 ± 8.4	53.9 ± 4.3	78.2 ± 6.8
**3c**	49.6 ± 3.9	29.3 ± 2.1	>100
**3d**	38.4 ± 2.7	18.1 ± 1.4 (SI = 3)	54.8 ± 4.4
**3e**	56.6 ± 4.4	>100	73.3 ± 6.4
**4a**	>100	>100	>100
**4b**	84.6 ± 7.5	26.2 ± 1.7	>100
**4c**	>100	>100	>100
**5a**	56.2 ± 4.7	1.9 ± 0.2 (SI = 73) ^a^	139.0 ± 11
**5b**	65.3 ± 5.4	52.1 ± 4.2	68.1 ± 5.4
**6a**	11.0 ± 8.7	1.7 ± 0.1 (SI = 24)	41 ± 3.5
**6b**	28.4 ± 1.8	64.3 ± 5.2	>100
**6c**	26.6 ± 1.9	7.3 ± 0.6 (SI = 4.6)	33.4 ± 2.8
**6d**	53.2 ± 4.6	18.5 ± 1.5 (SI = 3)	56.2 ± 4.7
**7a**	73.5 ± 6.2	59.7 ± 4.6	>100
**7b**	65.8 ± 5.8	55.4 ± 4.5	>100
**8**	85.2 ± 7.4	58.4 ± 4.6	100 ± 9.2
**9a**	>100	>100	>100
**9b**	97.8 ± 8.2	>100	95.4 ± 8.7
**10a**	>100	>100	>100
**11a**	>100	>100	>100
**11b**	94.3 ± 7.9	11.5 ± 0.9	53.1 ± 4.3
**Sorafenib**	35.6 ± 2.8	5.0 ± 0.4 (SI = 7)	35.0 ± 2.7
**Doxorubicin**	3.0 ± 0.2	3.0 ± 0.2 (SI = 1)	3.0 ± 0.1

^a^ Selectivity index (SI) = IC_50_(normal)/IC_50_(tumoral). The term “>100” indicates that no IC_50_ value was reached up to 100 μM.

**Table 2:** Additional cytotoxic profile for the studied lead compounds **5a** and **6a**.

Test Compound	IC_50_ (µM)				
	Cancer Cell Lines				
	Hep-2	SH-SY5Y	A549	PA-1	MCF-7
**5a**	65.76 ± 1.18	75.78 ± 2.84	82.08 ± 3.56	80.22 ± 4.05	85.63 ± 3.12
**6a**	2.34 ± 0.01	10.86 ± 0.23	5.47 ± 0.07	11.20 ± 1.36	7.12 ± 0.76

Hep-2-human epithelial cells, SH-SY5Y-human neuroblast-like cells, A549-human lung adenocarcinoma, PA-1-human ovarian teratocarcinoma and MCF-7-human mammary ductal adenocarcinoma.

## Data Availability

Data are contained within the article and Supplementary Materials.

## Supplementary Materials

The following supporting information can be downloaded at: https://www.mdpi.com/article/10.3390/ijms25126724/s1.
