# Supplementary material for: Discovery of Di(het)arylmethane and Dibenzoxanthene Derivatives as Potential Anticancer Agents
Source: Int J Mol Sci. 2024 Jun 18;25(12):6724. doi: 10.3390/ijms25126724 (PMC11203978; doi:10.3390/ijms25126724)
Supplement: Supplementary file 1 [file ijms-25-06724-s001.zip › ijms-3033624-supplementary.pdf]

Supporting information

Discovery of di(het)arylmethane and dibenzoxanthene derivatives as potential anticancer agents

Andrey Smolobochkin [1,], Dinara Niyazova [2], Almir Gazizov [1], Marat Syzdykbayev [2,3], Alexandra Voloshina [1], Syumbelya Amerhanova [1], Anna Lyubina [1], Margarita Neganova [1,4], Yulia Aleksandrova [1,4], Olga Babaeva [1], Julia Voronina [5], Nurbol Appazov [2,6,*], Oleg Sinyashin [1], Igor Alabugin [1,7], Alexander Burilov [1] and Michail Pudovik [1]*

[1] Arbuzov Institute of Organic and Physical Chemistry, FRC Kazan Scientific Center, Russian Academy of Sciences, Arbuzov str., 8, Kazan 420088, Russia

[2] Korkyt Ata Kyzylorda University, Ayteke bi str., 29A, Kyzylorda 120014, Kazakhstan

[3] Nazarbayev Intellectual School Chemical-Biological Direction in Kyzylorda, Sultan Beybars str., 6, Kyzylorda 120014, Kazakhstan

[4] Institute of Physiologically Active Compounds at Federal Research Center of Problems of Chemical Physics and Medicinal Chemistry, Russian Academy of Sciences, Severnij pr. 1, Chernogolovka 142432, Russia

[5] N.S. Kurnakov Institute of General and Inorganic Chemistry, Russian Academy of Sciences, Leninskii pr., 31, Moscow 119071, Russia

[6] Limited Liability Partnership «DPS-Kyzylorda», Amangeldi str., 112A, Kyzylorda 120014, Kazakhstan

[7] Department of Chemistry and Biochemistry, Florida State University, Chieftan Way str., 95, Tallahassee, FL 32306-3290, USA

***** Correspondence: smolobochkin@iopc.ru (AS); nurasar.82@korkyt.kz (NA)

Single crystal X-ray analysis

The X-ray diffraction data for the crystals of **6a** and **6b** were collected on a Bruker D8 Venture diffractometer equipped with a CCD detector (Mo-Kα, λ = 0.71073 Å, graphite monochromator). Semi-empirical absorption correction was applied by the SADABS program [1]. The structures were solved by direct methods and refined by the full-matrix least squares in the anisotropic approximation for non-hydrogen atoms. The calculations were carried out by the SHELX-2014 program package [2] using Olex2 1.2 [3]. The crystallographic parameters for **6a**, **6b** and the structure refinement details are given in Table 1,2. Crystallographic data for structures reported in this paper have been deposited with the Cambridge Crystallographic Data Center (2296793-2296794).

Crystal Data for **6a**: $C_{43}H_{35}ClNO_2P$ (M =664.14 g/mol): triclinic, space group P-1 (no. 2), a = 9.0714(8) Å, b = 12.9754(11) Å, c = 14.4897(11) Å, α = 85.122(3)°, β = 78.483(3)°, γ = 83.441(4)°, V = 1656.8(2) Å^3, Z = 2, T = 100.0 K, μ(MoKα) = 0.204 mm^{-1}, $Dcalc$ = 1.331 g/cm^3, 12550 reflections measured (2.874° ≤ 2Θ ≤ 51.998°), 6426 unique (R_{int} = 0.0462, R_{sigma} =0.0896) which were used in all calculations. The final R_1 was 0.0565 (I > 2σ(I)) and wR_2 was 0.1082 (all data).

Crystal Data for **6b**: $C_{40}H_{39}ClNO_3P$ (M =648.14 g/mol): monoclinic, space group C2/c (no. 15), a = 23.9191(9) Å, b = 9.4464(4) Å, c = 30.5142(10) Å, β = 106.717(3)°, V = 6603.3(4) Å^3, Z = 8, T = 150.0 K, μ(MoKα) = 0.205 mm^{-1}, $Dcalc$ = 1.304 g/cm^3, 26286 reflections measured (3.836° ≤ 2Θ ≤ 51.998°), 6489 unique (R_{int} = 0.0527, R_{sigma} = 0.0543) which were used in all calculations. The final R_1 was 0.0441 (I > 2σ(I)) and wR_2 was 0.0960 (all data).

Compounds **6a** and **6b** in the crystals are salts with a chlorine counterion bound to a molecule of the basic substance by a hydrogen bond of the fighting type (Table S1). In addition, the crystal of compound **6b** is a crystal solvate with ethanol, in a ratio of 1:1. The ethanol molecule also forms a hydrogen bond with a chlorine ion (Table S1). The molecules of compounds **6a** and **6b** in crystals are a voluminous polycyclic fragment with a sufficiently long substituent containing a urea group and ending with triphenylphosphanyl (**6a**) and methyl(diphenyl)phosphanyl (**6b**) groups. Polycyclic fragments of both molecules have a non-planar configuration. However, the angle between the planes of benzene cycles in **6a** (26.51(6)˚) is significantly greater than in **6b** (8.15(5)˚). Analysis of the CCBC data showed that 26 structures with the same polycyclic fragments have been described to date. The angle between the corresponding planes varies from 3 to 39 degrees, in most structures the angle is 14-17˚. Apparently, the configuration of this fragment is determined by non-covalent interactions and other packaging effects. The phosphonyl fragments in the molecules are deployed in different directions. In compound **6a**, as it were, inside the polycycle, stabilizing by CH...π interaction involving one of the phenyl rings, and in compound **6b** - from the polycycle, being stabilized only by weak intermolecular interactions.

The packing of molecules in both crystals is formed due to hydrogen bonds, primarily with the participation of chlorine ion, and pi-pi interactions. Due to hydrogen bonds, infinite chains directed along the 0b axis are formed, and π…π overlapping of polycyclic fragments stitch them into layers

parallel to the b0c plane in **6b** and a three-dimensional grid in **6a** (table S2, fig. S2). The formation of the three-dimensional structure of **6b** and the stabilization of the crystal **6a** occurs due to weaker CH…O and CH…π interactions.

REFERENCES

1. G.M. Sheldrick. SADABS. Bruker AXS Inc., Madison, WI-53719, USA, 1997.
2. G.M. Sheldrick. SHELXT 2014/4 (Sheldrick, 2014).
3. Dolomanov, O.V.; Bourhis, L.J.; Gildea, R.J.; Howard, J.A.K.; Puschmann, H., OLEX2: A complete structure solution, refinement and analysis program (2009). J. Appl. Cryst., 42, 339-341.

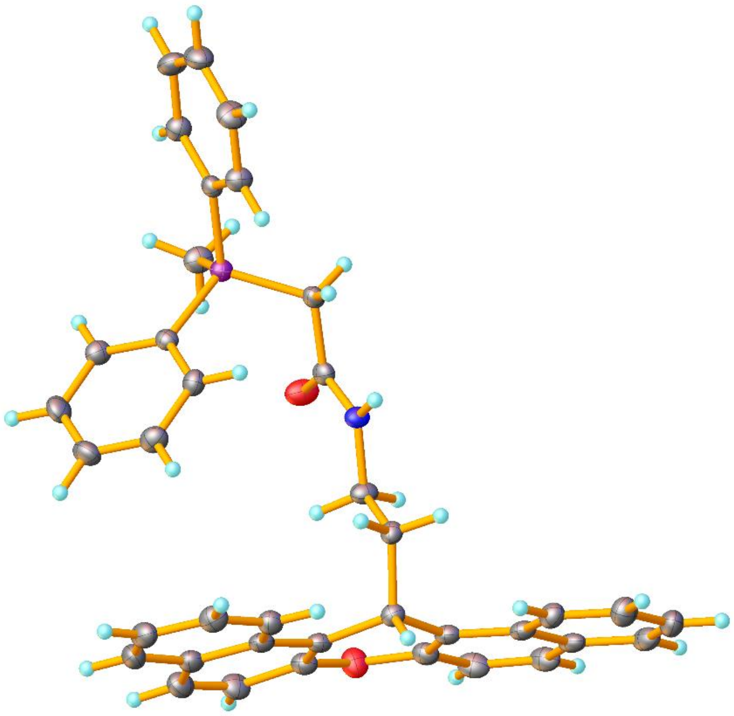

Figure S1. Molecular structure of investigated compounds **6a** and **6b** on the example of the molecule **6b.** Ellipsoids are given with a 50% probability.

Table S1. H-bonds in crystals of investigated compounds.

H-bond	D-H, Å	H...A, Å	D...A, Å	D-H...A, ˚
**1**				
N1-H1...Cl1	0.88	2.55	3.3480(18)	151
O1S-H1S...Cl1	0.84	2.27	3.1063(19)	176
C1-H1A...Cl1	0.99	2.51	3.4795(19)	168
C1-H1B...Cl1	0.99	2.56	3.502(2)	159
C9-H9...O1	0.95	2.59	3.225(3)	125
C28-H28...O1S	0.95	2.46	3.361(3)	157
C32-H32...Cl1	0.95	2.75	3.690(2)	171
C39-H39B...O1S	0.98	2.52	3.470(3)	163
C39-H39C...O1	0.98	2.58	3.154(3)	117

2				
N1-H1...Cl1	0.88	2.33	3.192(2)	165
C1-H1B...Cl1	0.99	2.53	3.435(3)	152
C32-H32...O1	0.95	2.41	3.212(3)	142
C34-H34...Cl1	0.95	2.78	3.427(3)	126
C38-H38...Cl1	0.95	2.59	3.523(3)	169
C42-H42...O1	0.95	2.58	3.484(3)	159

Table S2. π...π interactions in crystals of investigated compounds.

Interaction	Cg-Cg, Å	Alpha, ˚	Beta, ˚	Gamma, ˚	CgI_Perp, Å
**6b**					
Cg2...Cg5	3.6511(12)	6.94(9)	18.9	22.3	3.3785(8)
Cg5...Cg2	3.6510(12)	6.94(9)	22.3	18.9	3.4537(8)
Cg5...Cg9	3.5054(10)	4.15(8)	8.7	10.5	3.4467(8)
Cg5...Cg13	3.6840(10)	0.87(7)	20.7	21.5	3.4267(8)
Cg5...Cg14	3.4470(10)	6.32(7)	15.1	9.8	3.3969(8)
Cg5...Cg16	3.3525(9)	4.41(7)	4.2	7.7	3.3226(8)
Cg5...Cg17	3.5719(10)	4.19(7)	20.0	21.5	3.3229(8)
Cg9...Cg5	3.5054(10)	4.15(8)	10.5	8.7	3.4651(6)
Cg11...Cg16	3.5369(8)	5.67(5)	18.7	21.1	3.2990(6)
Cg11...Cg17	3.4670(8)	5.45(4)	12.4	17.8	3.3016(6)
Cg12...Cg17	3.5851(7)	5.77(3)	18.5	19.8	3.3737(5)
Cg13...Cg5	3.6840(10)	0.87(7)	21.5	20.7	3.4467(5)
Cg14...Cg5	3.4470(10)	6.32(7)	9.8	15.1	3.3272(5)
Cg16...Cg5	3.3525(9)	4.41(7)	7.7	4.2	3.3435(5)
Cg16...Cg11	3.5369(8)	5.67(5)	21.1	18.7	3.3511(5)
Cg17...Cg5	3.5719(10)	4.19(7)	21.5	20.0	3.3561(4)
Cg17...Cg11	3.4669(8)	5.45(4)	17.8	12.4	3.3861(4)
Cg17...Cg12	3.5850(7)	5.77(3)	19.8	18.5	3.3997(4)
**6a**					
Cg4...Cg4	3.8464(17)	0.00(13)	12.2	12.2	3.7591(11)
Cg4...Cg18	3.5427(13)	16.49(10)	28.6	14.0	3.4366(11)
Cg5...Cg6	3.9294(17)	7.27(13)	28.9	33.5	3.2766(12)
Cg5...Cg18	3.9461(13)	16.29(10)	38.0	29.8	3.4260(12)
Cg6...Cg5	3.9295(17)	7.27(13)	33.5	28.9	3.4401(11)
Cg12...Cg18	3.5453(11)	16.39(6)	28.4	14.5	3.4317(9)
Cg18...Cg4	3.5426(13)	16.49(10)	14.0	28.6	3.1115(6)
Cg18...Cg5	3.9461(13)	16.29(10)	29.8	38.0	3.1091(6)
Cg18...Cg12	3.5453(11)	16.39(6)	14.5	28.4	3.1188(6)

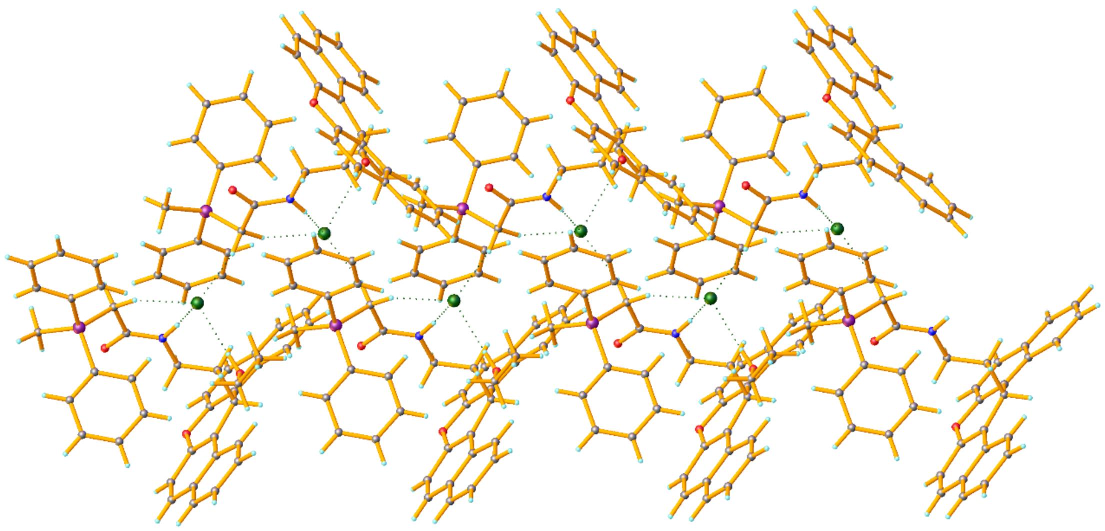

a

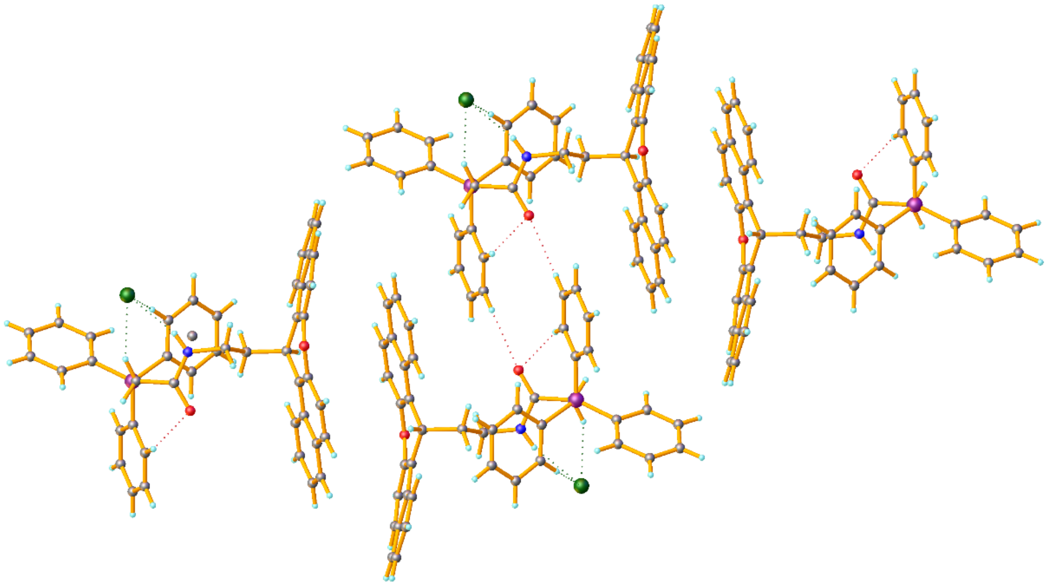

b

Figure S2. a) Fragment of H-bonded chain in crystal of **6b**. b) H-bonds and π…π interactions in fragment of crystal **6a**.

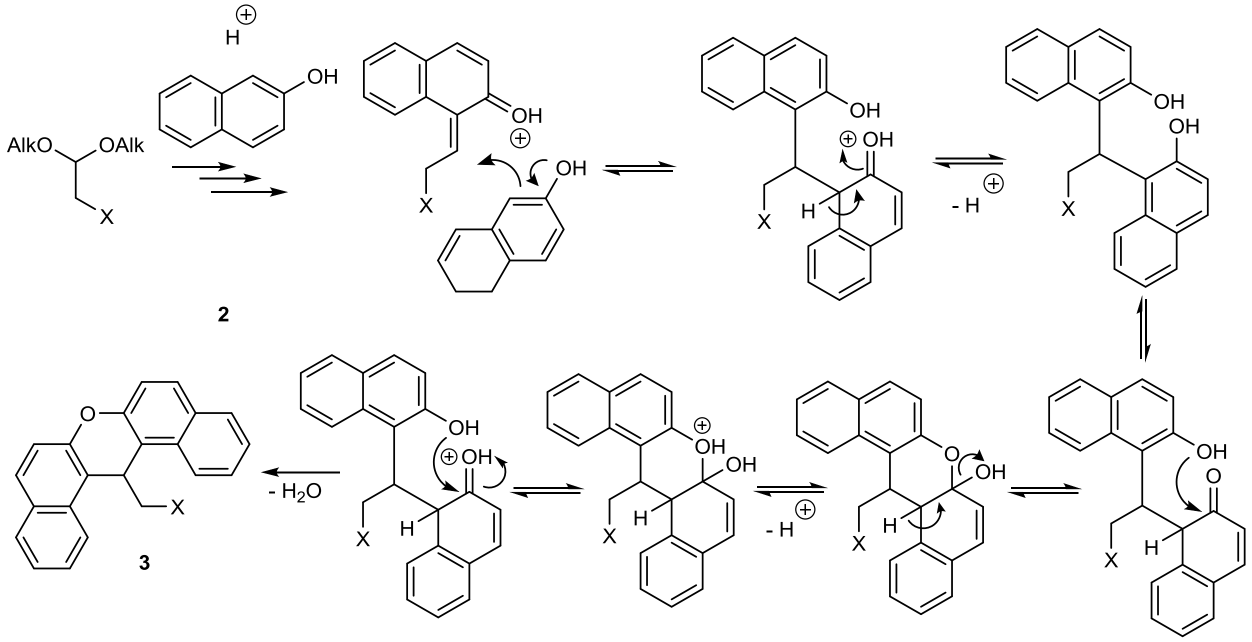

Scheme S1. Proposed mechanism for the dibenzoxanthene **3** formation

Almir S. Gazizov , Andrey V. Smolobochkin, Ekaterina A. Anikina , Anna G. Strelnik , Alexander R. Burilov , Michail A. Pudovik. Acid-Mediated C–N Bond Cleavage in 1-Sulfonylpyrrolidines: An Efficient Route towards Dibenzoxanthenes, Diarylmethanes, and Resorcinarenes. Synlett 2018; 29 (04): 467-472. DOI: 10.1055/s-0036-1590954/

Table S3. IC$_{50}$ calculation chart

Cell lines	IC$_{50}$ Charts		
	Compounds		
	**5a**	**6a**	**Doxorubicin**
**HuTu 80**	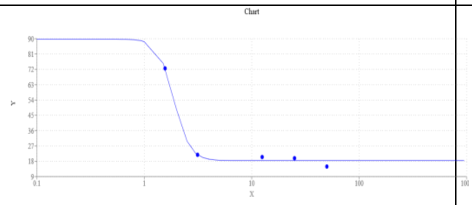	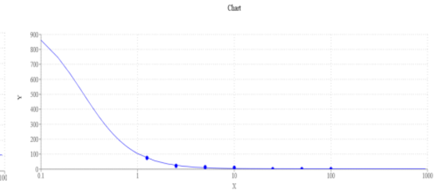	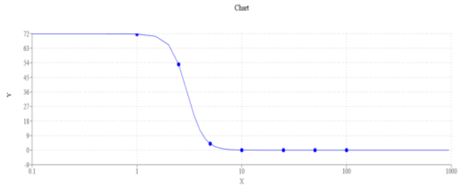
**M-HeLa**	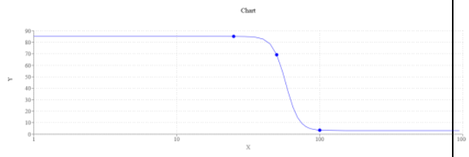	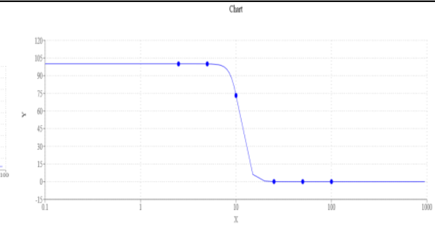	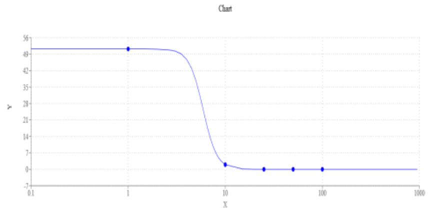
**Chang liver**	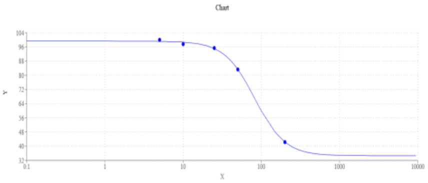	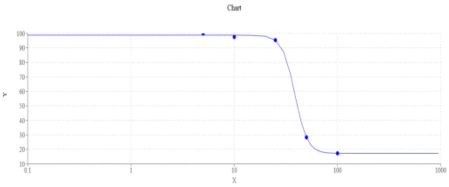	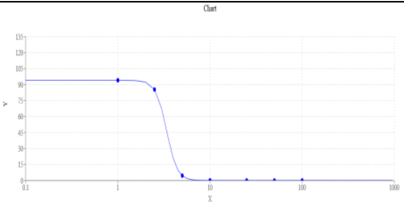

EXPERIMENTAL SECTION

General. The ^{1}H and ^{13}C NMR spectra were recorded on a Bruker Avance 600 spectrometer (operating frequency 600 MHz and 150 MHz, respectively) with respect to the residual proton signals of deuterated solvents (DMSO-d_6, CD_3OD, $CDCl_3$). The IR spectra were recorded on a Vector 22 Fourier spectrometer by Bruker in the range of 400-4000 cm^{-1} from KBr pellets. The melting points were determined in glass capillaries on a Stuart SMP 10 instrument. The elemental analysis was carried out on a CHNS analyzer Vario Macro cube (Elementar Analysensysteme GmbH, Germany). The samples were weighed on Sartorius Cubis II (Germany) microbalance in tin capsules. VarioMacro Software V4.0.11 was used to perform quantitative measurements and evaluate the data received. The halogen content was determined by the Schöniger method.

Electrospray ionization measurements were performed using UHR-QTOF Impact II mass spectrometer with Elute UHPLC system (Bruker Daltonik GmbH, Germany). The column YMC-Triart C18 (50×2.0 mm; 3µm) was used. The column thermostat temperature was set at 40°C and the autosampler temperature at 12°C. Elution solvents were used Milli-Q water + 0.1% formic acid (A) and HPLC-grade methanol + 0.015% ammonium acetate (B) and elution gradient was the following: 0 min at 5% B, 3 min at 95% B, 6 min at 95% B, 6.1 min at 5% B, 8 min at 5% B with a flow rate of 0.3 mL/min. The injection volume was 2 µL. Measurements were made in positive mode in the range *m/z* 50-1900. The ESI source conditions were as follows: capillary voltage 4500 V, desolvation temperature 220°C, drying gas (N_2) at flow rate of 6 L/min. The samples were prepared in HPLC-grade methanol with a concentration of 0.002 mg/ml. The solution of sodium iodide in Milli-Q water (0.2 mg/mL) was used as a calibrant. The relative error in determining the exact mass values was no more than 5 ppm. The *m/z* values of monoisotopic ions in the ion cluster are given in the description.

The Hystar (Bruker Daltonik GmbH, version 6.0), the otofControl (Bruker Daltonik GmbH, version 5.2) programs were used to control the chromatograph and mass spectrometer. Data processing was performed by DataAnalysis software (Bruker Daltonik GmbH, version 5.3).

The X-ray diffraction data for the crystals of **6a**,**6b** were collected on a Bruker D8 Venture diffractometer equipped with a CCD detector (Mo-Kα, λ = 0.71073 Å, graphite monochromator). Semi-empirical absorption correction was applied by the SADABS program [36]. The structures were resolved by direct methods and refined by the full-matrix least squares in the anisotropic approximation for non-hydrogen atoms. The calculations were carried out by the SHELX-2014 program package [37] using Olex2 1.2 [38]. The crystallographic parameters for **6a**,**6b** and the structure refinement details are given in Supporting Information. Crystallographic data for structures reported in this paper have been deposited with the Cambridge Crystallographic Data Center (2296793, 2296794).

General Experimental Procedure for the Synthesis of 2a-c. 2-Chloroacetyl chloride (2.9 g, 26 mmol, 1 equiv) was added to a solution of acetal **1** (1.64 mmol, 1 equiv) in dry CH_2Cl_2 (20 mL) at a temperature of 5–10 °C for 2 h. The solvent was eliminated under reduced pressure. The residue was washed with 20 mL of benzene and dried in vacuo (10 torr, 4 h, 20 °C).

2-Chloro-N-(2,2-dimethoxyethyl)acetamide (**2a**). Yellow oil; yield 68%; IR (KBr, cm^{-1}) 1670, 2836, 2945; ^{1}H NMR (600 MHz, DMSO-d_6) δ 8.26 (s, 1H), 4.38 (t, 1H, *J* = 5.3 Hz), 4.08 (s, 2H), 3.28 (s, 6H), 3.21 (t, 2H, *J* = 5.7 Hz); ^{13}C NMR (150 MHz, DMSO-d_6) δ 166.66, 102.24, 53.81, 42.98, 41.27. Anal. Calcd for $C_6H_{12}ClNO_3$: C, 39.68; H, 6.66; Cl, 19.52; N, 7.71. Found: C, 39.83; H, 6.84; Cl, 19.40; N, 7.85.

2-Chloro-N-(3,3-diethoxypropyl)acetamide (**2b**). Yellow oil; yield 95%; IR (KBr, cm^{-1}) 1664, 2883, 2932; ^{1}H NMR (600 MHz, $CDCl_3$) δ 7.30 (s, 1H), 4.33 (t, 1H, *J* = 5.0 Hz), 3.78 (s, 2H), 3.47-3.38 (m, 2H), 3.30-3.22 (m, 2H), 3.18-3.10 (m, 2H), 1.65-1.53 (m, 2H), 0.96 (t, 6H, *J* = 7.1 Hz); ^{13}C NMR (150 MHz,

$CDCl_3$) δ 165.71, 102.09, 61.84, 42.42, 35.54, 32.53, 15.07. Anal. Calcd for $C_9H_{18}ClNO_3$: C, 48.32; H, 8.11; Cl, 15.85; N, 6.26. Found: C, 48.21; H, 8.21; Cl, 15.76; N, 6.35.

2-Chloro-N-(4,4-diethoxybutyl)acetamide (**2c**). Yellow oil; yield 62%; IR (KBr, cm^{-1}) 1663, 2948. ^1H NMR (600 MHz, $CDCl_3$) δ 6.79 (s, 1H), 4.48 (t, 1H, *J* = 4.9 Hz), 4.01 (s, 2H), 3.67-3.61 (m, 2H), 3.50-3.42 (m, 2H), 3.35-3.27 (m, 2H), 1.71-1.57 (m, 4H), 1.18 (t, 6H, *J* = 7.1 Hz); ^{13}C NMR (150 MHz, $CDCl_3$) δ 165.81, 102.52, 61.46, 42.62, 39.50, 30.87, 24.34, 15.27. Anal. Calcd for $C_{10}H_{20}ClNO_3$: C, 50.52; H, 8.48; Cl, 14.91; N, 5.89. Found: C 50.43; H 8.56; Cl 14.84; N 5.95.

General Experimental Procedure for the Synthesis of 3,4,5. Acetal **2** (2 mmol, 1 equiv) was added to a solution of phenol (4 mmol, 2 equiv), trifluoroacetic acid (0.15 ml, 2 mmol, 1 equiv) in dry chloroform (10 mL). The reaction mixture was stirred at room temperature for 50 hours. The solvent was eliminated under reduced pressure. The residue was washed with 10 mL of diethyl ether and dried in vacuo (10 torr, 10 h, 20 °C).

N-((14H-dibenzo[a,j]xanthen-14-yl)methyl)-2-chloroacetamide (**3a**). White solid; yield 40%; mp 200-203 °C. IR (KBr, cm^{-1}) 1435, 1459, 1517, 1663; ^1H NMR (600 MHz, DMSO-d_6) δ 8.52 (d, 2H, *J* = 8.5 Hz), 8.22 (t, 1H, *J* = 6.2 Hz), 7.96 (d, 2H, *J* = 8.0 Hz), 7.92 (d, 2H, *J* = 8.8 Hz), 7.67 (t, 2H, *J* = 7.1 Hz), 7.51 (t, 2H, *J* = 7.4 Hz), 7.46 (d, 2H, *J* = 8.8 Hz), 5.79 (t, 1H, *J* = 6.3 Hz), 3.74 (s, 2H), 3.45 (t, 2H, *J* = 6.1 Hz); ^{13}C NMR (150 MHz, DMSO-d_6) δ 166.78, 150.28, 132.11, 131.08, 129.33, 129.02, 127.36, 124.95, 123.25, 117.80, 116.01, 45.86, 42.81, 30.71. Anal. Calcd for $C_{24}H_{18}ClNO_2$: C, 74.32; H, 4.68; Cl, 9.14; N, 3.61. Found: C, C 74.43; H 4.56; Cl 9.22; N 3.54. MS (ESI) *m/z* calcd for 387.8, found 388.1 $[M + H]^+$.

2-Chloro-N-((2,12-dihydroxy-14H-dibenzo[a,j]xanthen-14-yl)methyl)acetamide (**3b**). White solid; yield 55%; mp >250 °C. IR (KBr, cm^{-1}) 1453, 1518, 3438; ^1H NMR (600 MHz, DMSO-d_6) δ 9.82 (s, 2H), 8.09 (t, 1H, *J* = 6.1 Hz), 8.18-8.00 (m, 4H), 7.66 (d, 2H, *J* = 2.4 Hz), 7.20 (d, 2H, *J* = 8.8 Hz), 7.10 (dd, 2H, *J* = 8.7 Hz, *J* = 2.3 Hz), 5.40 (t, 1H, *J* = 6.0 Hz), 3.83 (s, 2H), 3.48-3.41 (m, 2H); ^{13}C NMR (150 MHz, DMSO-d_6) δ 166.71, 156.98, 150.78, 133.91, 130.52, 128.95, 125.54, 117.10, 114.47, 114.35, 105.29, 45.02, 42.68, 30.95. Anal. Calcd for $C_{24}H_{18}ClNO_4$: C, 68.66; H, 4.32; Cl, 8.44; N, 3.34. Found: C, 68.83; H, 4.26; Cl, 8.36; N, 3.57. MS (ESI) *m/z* calcd for 419.8, found 421.2 $[M + H]^+$.

N-(2-(14H-dibenzo[a,j]xanthen-14-yl)ethyl)-2-chloroacetamide (**3c**). White solid; yield 76%; mp 147-149 °C. IR (KBr, cm^{-1}) 1434, 1458, 1516, 1660, 2940; ^1H NMR (600 MHz, DMSO-d_6) δ 8.57 (d, 2H, *J* = 8.5 Hz), 7.98 (d, 2H, *J* = 8.1 Hz), 7.92 (d, 2H, *J* = 9.0 Hz), 7.90 (br s, 1H), 7.68 (t, 2H, *J* = 7.7 Hz), 7.52 (t, 2H, *J* = 7.4 Hz), 7.45 (d, 2H, *J* = 8.8 Hz), 5.75 (t, 1H, *J* = 4.9 Hz), 3.78 (s, 2H), 2.79-3.71 (m, 2H), 2.16-2.09 (m, 2H); ^{13}C NMR (150 MHz, DMSO-d_6) δ 165.84, 149.79, 131.48, 131.19, 129.18, 127.47, 124.98, 123.51, 117.67, 116.47, 42.92, 36.27, 35.65, 28.97. Anal. Calcd for $C_{25}H_{20}ClNO_2$: C, 74.72; H, 5.02; Cl, 8.82; N, 3.49. Found: C, 74.83; H, 4.91; Cl, 18.72; N, 3.61. MS (ESI) *m/z* calcd for 401.8, found 402.3 $[M + H]^+$, 424.8 $[M + Na]^+$.

2-Chloro-N-(2-(2,12-dihydroxy-14H-dibenzo[a,j]xanthen-14-yl)ethyl)acetamide (**3d**). White solid; yield 60%; mp 137-139 °C. IR (KBr, cm^{-1}) 1437, 1469, 1543, 1661, 2952; ^1H NMR (600 MHz, DMSO-d_6) δ 9.90 (s, 2H), 7.94 (t, 1H, *J* = 5.7 Hz), 7.82 (d, 2H, *J* = 8.6 Hz), 7.78 (d, 2H, *J* = 8.7 Hz), 7.60 (s, 2H), 7.19 (d, 2H, *J* = 8.5 Hz), 7.11 (d, 2H, *J* = 8.4 Hz), 5.28 (t, 1H, *J* = 4.7 Hz), 3.81 (s, 2H), 2.79-2.68 (m, 2H), 2.19-2.10 (m, 2H); ^{13}C NMR (150 MHz, DMSO-d_6) δ 170.76, 165.83, 157.08, 150.16, 133.14, 130.83, 128.92, 125.59, 117.09, 114.20, 105.17, 60.21, 42.91, 36.27, 34.27. Anal. Calcd for $C_{25}H_{20}ClNO_4$: C, 69.21; H, 4.65; Cl, 8.17; N, 3.23. Found: C, 69.38; H, 4.87; Cl, 8.05; N, 3.15.

N-(3-(14H-dibenzo[a,j]xanthen-14-yl)propyl)-2-chloroacetamide (**3e**). White solid; yield 69%; mp 164-165 °C. IR (KBr, cm^{-1}) 1435, 1458, 1516, 1655; ^1H NMR (600 MHz, DMSO-d_6) δ 8.53 (d, 2H, *J* = 8.5 Hz), 7.96 (d, 2H, *J* = 8.0 Hz), 7.93-7.82 (m, 3H), 7.67 (t, 2H, *J* = 7.7 Hz), 7.50 (t, 2H, *J* = 7.4 Hz), 7.43 (d, 2H, *J* = 8.9 Hz), 5.73 (t, 1H, *J* = 4.6 Hz), 3.81 (s, 2H), 2.83-2.69 (m, 2H), 1.98-1.86 (m, 2H), 1.10-1.02 (m, 2H); ^{13}C NMR (150 MHz, DMSO-d_6) δ 165.88, 149.84, 131.53, 131.17, 129.14, 128.99, 127.39, 124.89, 123.54, 117.65, 116.80, 42.94, 39.39, 33.68, 30.32, 25.25. Anal. Calcd for $C_{26}H_{22}ClNO_2$: C, 75.08; H, 5.33; Cl, 8.52; N, 3.37. Found: C, 75.23; H, 5.18; Cl, 8.67; N, 3.31. MS (ESI) *m/z* calcd for 415.9, found 416.7 $[M + H]^+$.

2-Chloro-N-(3-(2,12-dihydroxy-14H-dibenzo[a,j]xanthen-14-yl)propyl)acetamide (**3f**). White solid; yield 53%; mp 180 °C. IR (KBr, cm^{-1}) 15145, 1656, 2975, 3006; ^1H NMR (600 MHz, Acetone-d_6) δ 8.76 (br s, 2H), 8.81 (d, 2H, *J* = 8.8 Hz), 7.75 (d, 2H, *J* = 8.8 Hz), 7.69 (s, 2H), 7.33 (br s, 1H), 7.17 (d, 2H, *J* = 8.8 Hz), 7.14 (dd, 2H, *J* = 8.7, 2.3 Hz), 5.37 (t, 1H, *J* = 4.6 Hz), 3.89 (s, 2H), 3.10-3.04 (m, 2H), 2.11-2.07 (m, 2H), 1.31-1.21 (m, 2H); ^{13}C NMR (150 MHz, Acetone-d_6) δ 165.80, 156.42, 150.42, 133.21, 130.42, 128.22, 125.89, 116.28, 114.96, 114.18, 104.97, 42.30, 39.34, 32.42, 30.44, 25.16. Anal. Calcd for $C_{26}H_{22}ClNO_4$: C, 69.72; H, 4.95; Cl, 7.91; N, 3.13. Found: C, 69.93; H, 4.88; Cl, 7.77; N 3.29. MS (ESI) *m/z* calcd for 447.9, found 448.6 $[M + H]^+$, 470.4 $[M + Na]^+$.

N-(2,2-bis(6-hydroxybenzo[d][1,3]dioxol-5-yl)ethyl)-2-chloroacetamide (**4a**). White solid; yield 54%; mp 180-182 °C. IR (KBr, cm^{-1}) 1439, 1489, 1505, 1643, 2924, 3365; ^1H NMR (600 MHz, DMSO-d_6) δ 9.05 (br s, 2H), 8.00 (br s, 1H), 6.62 (s, 2H), 6.41 (s, 2H), 5.85 (d, 4H, *J* = 12.8 Hz), 4.64 (t, 1H, *J* = 7.9 Hz), 3.98 (s, 2H), 3.61-3.50 (m, 2H); ^{13}C NMR (150 MHz, DMSO-d_6) δ 166.20, 149.98, 145.93, 140.00, 120.75, 108.36, 100.88, 98.07, 43.09, 42.32, 37.27. Anal. Calcd for $C_{18}H_{16}ClNO_7$: C, 54.90; H, 4.10; Cl, 9.00; N, 3.56. Found: C 55.13; H 4.26; Cl 9.06; N 3.59.

N-(3,3-bis(6-hydroxybenzo[d][1,3]dioxol-5-yl)propyl)-2-chloroacetamide (**4b**). White solid; yield 87%; mp 166-168 °C. IR (KBr, cm^{-1}) 1438, 1486, 1504, 1658, 2764, 2786, 3015, 3090; ^1H NMR (600 MHz, DMSO-d_6) δ 8.97 (br s, 2H), 8.13 (br s, 1H), 6.69 (s, 2H), 6.39 (s, 2H), 5.84 (d, 4H, *J* = 13.2 Hz), 4.42 (t, 1H, *J* = 7.8 Hz), 4.01 (s, 2H), 3.04-2.95 (m, 2H), 2.02-1.95 (m, 2H); ^{13}C NMR (150 MHz, DMSO-d_6) δ 166.04, 149.56, 145.62, 140.10, 122.80, 108.12, 100.84, 97.99, 43.16, 38.72, 34.66, 33.85. Anal. Calcd for $C_{19}H_{18}ClNO_7$: C, 55.96; H, 4.45; Cl, 8.69; N, 3.43. Found: C, 55.83; H, 4.56; Cl, 8.82; N, 3.31.

N-(4,4-bis(6-hydroxybenzo[d][1,3]dioxol-5-yl)butyl)-2-chloroacetamide (**4c**). White solid; yield 74%; mp 189-191°C. IR (KBr, cm^{-1}) 1453, 1485, 1502, 1656, 2897, 3118, 3351; ^1H NMR (600 MHz, DMSO-d_6) δ 8.91 (br s, 2H), 8.12 (br s, 1H), 6.67 (s, 2H), 6.38 (s, 2H), 5.84 (d, 4H, *J* = 11.4 Hz), 4.41 (t, 1H, *J* = 7.9 Hz), 3.99 (s, 2H), 3.16-3.02 (m, 2H), 1.86-1.76 (m, 2H), 1.41-1.25 (m, 2H); ^{13}C NMR (150 MHz, DMSO-d_6) δ 166.10, 149.55, 145.48, 140.11, 123.32, 108.04, 100.81, 98.01, 43.16, 39.62, 36.15, 31.61, 28.22. Anal. Calcd for $C_{20}H_{20}ClNO_7$: C, 56.95; H, 4.78; Cl, 8.40; N, 3.32. Found: C, 56.83; H, 4.88; Cl, 8.56; N, 3.21.

N-(3,3-bis(4-hydroxy-6-methyl-2-oxo-2H-pyran-3-yl)propyl)-2-chloroacetamide (**5a**). White solid; yield 66%; mp 132-134 °C. IR (KBr, cm^{-1}) 1455,1647, 2873, 2943; ^1H NMR (600 MHz, Acetone-d_6) δ 6.13 (s, 2H), 4.42 (t, 1H, *J* = 7.9 Hz), 4.03 (s, 2H), 3.26 (t, 2H, *J* = 6.9 Hz), 2.42 (q, 2H, *J* = 7.2 Hz), 2.25 (s, 6H); ^{13}C NMR (150 MHz, Acetone-d_6) δ 168.70, 168.38, 165.66, 161.57, 103.54, 102.25, 42.34, 38.01, 28.12, 18.48. Anal. Calcd for $C_{17}H_{18}ClNO_7$: C, 53.20; H, 4.73; Cl, 9.24; N, 3.65. Found: C, C 53.34; H, 4.69; Cl, 9.17; N, 3.44. MS (ESI) *m/z* calcd for 383.7, found 384.2 $[M + H]^+$, 406.2 $[M + Na]^+$, 423.5 $[M + K]^+$. ESI-HRMS, *m/z*: $[M+H^+]^+$ calcd for $C_{17}H_{19}ClNO_7$ 384.0845, found 384.0847; $[M+Na^+]^+$ calcd for $C_{17}H_{18}ClNNaO_7$ 406.0664, found 406.0666.

N-(3,3-bis(4-hydroxy-2-oxo-2H-chromen-3-yl)propyl)-2-chloroacetamide (**5b**). White solid; yield 63%; mp 134 °C. IR (KBr, cm^{-1}) 1453, 1495, 1656, 2725, 2873, 2946; ^1H NMR (600 MHz, $CDCl_3$) δ 12.04 (br s, 2H), 8.01 (d, 2H, *J* = 7.2 Hz), 7.61 (d, 2H, *J* = 7.4 Hz), 7.43-7.33 (m, 4H), 6.70 (s, 1H), 4.60 (t, 1H, *J* = 7.7 Hz), 3.99 (s, 2H), 3.47-3.33 (m, 2H), 2.80-2.63 (m, 2H); ^{13}C NMR (150 MHz, $CDCl_3$) δ 166.09, 152.33, 152.17, 132.77, 124.92, 124.83, 124.30, 124.06, 116.54, 105.11, 42.52, 38.79, 30.50, 28.28. Anal. Calcd for $C_{23}H_{18}ClNO_7$: C, 60.60; H, 3.98; Cl, 7.78; N, 3.07. Found: C, 60.74; H, 4.15; Cl, 7.68; N, 2.94. MS (ESI) *m/z* calcd for 455.8, found 456.9 $[M + H]^+$.

N-(2,2-bis(4-hydroxy-2-oxo-2H-chromen-3-yl)ethyl)-2-chloroacetamide (**5c**). White solid; yield 47%; mp 187-188 °C. IR (KBr, cm^{-1}) 1462, 1495, 1667, 2817, 2971; ^1H NMR (600 MHz, DMSO-d_6) δ 7.94-7.86 (m, 2H), 7.61-7.51 (m, 2H), 7.38-7.25 (m, 4H), 5.58 (t, 1H, *J* = 7.3 Hz), 4.11 (s, 2H), 3.25-3.15 (m, 1H), 3.06-2.97 (m, 2H); ^{13}C NMR (150 MHz, DMSO-d_6) δ 166.17, 162.83, 153.19, 132.76, 124.88, 124.04, 117.36, 116.74, 105.89, 103.03, 45.38, 43.62, 28.28. Anal. Calcd for $C_{22}H_{16}ClNO_7$: C, 59.81; H, 3.65; Cl, 8.02; N, 3.17. Found: C, 59.98; H, 3.81; Cl, 7.92; N, 3.04.

General Experimental Procedure for the Synthesis of 6,7,8,9. Compound **3** or **4** (0.75 mmol, 1 equiv) was added to a solution of phosphine or pyridine (0.75 mmol, 1 equiv) in dry ethanol (10 mL). The reaction mixture was boiled for 72 hours. The solvent was eliminated under reduced pressure. The residue was washed with 20 mL of benzene and dried in vacuo (10 torr, 10 h, 20°C).

(2-((2-(14H-dibenzo[a,j]xanthen-14-yl)ethyl)amino)-2-oxoethyl)triphenylphosphonium chloride (**6a**). White solid; yield 76%; mp 235°C. IR (KBr, cm^{-1}) 1437, 1458, 1516, 1662, 2855, 2923; ^1H NMR (600 MHz, DMSO-d_6) δ 8.97 (s, 1H), 8.56 (d, 2H, *J* = 8.5 Hz), 7.98 (d, 2H, *J* = 8.1 Hz), 7.92 (d, 2H, *J* = 8.9 Hz), 7.73-7.62 (m, 11H), 7.59-7.50 (m, 8H), 7.43 (d, 2H, *J* = 8.8 Hz), 5.68 (t, 1H, *J* = 5.4 Hz), 4.59 (d, 2H, *J* = 15.2 Hz), 2.63-2.52 (m, 2H), 1.88-1.76 (m, 2H); ^{13}C NMR (150 MHz, DMSO-d_6) δ 162.66 (d, *J* = 5.0 Hz), 149.77, 135.15, 134.18 (d, *J* = 10.5 Hz), 131.46, 131.15, 130.25 (d, *J* = 12.7 Hz), 129.19 (d, *J* = 4.4 Hz), 127.51, 125.03, 123.60, 119.42, 118.84, 117.66, 116.56, 40.63, 36.00 (d, *J* = 154.9 Hz), 31.34 (d, *J* = 55.2 Hz), 28.78. ^{31}P NMR (161 MHz, DMSO-d_6) δ 22.10. Anal. Calcd for $C_{43}H_{35}ClNO_2P$: C, 77.76; H, 5.31; Cl, 5.34; N, 2.11; P, 4.66. Found: C, 77.84; H, 5.21; Cl, 5.47; N, 2.04; P, 4.63. MS (ESI) *m/z* calcd for 664.2, found 629.1 $[M + H - Cl]^+$. ESI-HRMS, *m/z*: $[M\text{-}Cl^-]^+$ calcd for $C_{43}H_{35}NO_2P$ 628.2400, found 628.2400.

(2-((2-(14H-dibenzo[a,j]xanthen-14-yl)ethyl)amino)-2-oxoethyl)(methyl)diphenylphosphonium chloride (**6b**). White solid; yield 87%; mp 161-162 °C. IR (KBr, cm^{-1}) 1458, 1516, 1552, 1664, 2942; ^1H NMR (600 MHz, DMSO-d_6) δ 8.63 (t, 1H, *J* = 5.5 Hz), 8.55 (d, 2H, *J* = 8.5 Hz), 7.99 (d, 2H, *J* = 7.3 Hz), 7.93 (d, 2H, *J* = 8.9 Hz), 7.81-7.76 (m, 4H), 7.71 -7.65 (m, 4H), 7.57 -7.51 (m, 6H), 7.44 (d, 2H, *J* = 8.8 Hz), 5.70 (t, 1H, *J* = 5.0 Hz), 4.23 (d, 2H, *J* = 15.2 Hz), 2.63-2.56 (m, 2H), 2.60 (d, 3H, *J* = 14.8 Hz), 1.92-1.86 (m, 2H); ^{13}C NMR (150 MHz, DMSO-d_6) δ 163.51 (d, *J* = 4.9 Hz), 150.30, 135.14, 133.18 (d, *J* = 10.4 Hz), 131.81 (d, *J* = 31.8 Hz), 130.55, 130.42, 129.68, 128.02, 125.53, 124.09, 121.78, 120.92, 118.17, 117.13, 36.52 (d, *J* = 79.6 Hz), 31.20 (d, *J* = 56.4 Hz), 29.31, 7.72 (d, *J* = 55.6 Hz). ^{31}P NMR (161 MHz, DMSO-d_6) δ 21.91. Anal. Calcd for $C_{38}H_{33}ClNO_2P$: C, 75.80; H, 5.52; Cl, 5.89; N, 2.33; P, 5.14. Found: C, 75.93; H, 5.70; Cl, 5.77; N, 2.21; P, 4.91. MS (ESI) *m/z* calcd for 601.2, found 567.3 $[M + H - Cl]^+$.

(2-((3-(14H-dibenzo[a,j]xanthen-14-yl)propyl)amino)-2-oxoethyl)triphenylphosphonium chloride (**6c**). White solid; yield 67%; mp 204-205 °C. IR (KBr, cm^{-1}) 1458, 1516, 1552, 1664, 2942; ^1H NMR (600 MHz, DMSO-d_6) δ 8.72 (t, 1H, *J* = 5.6 Hz), 8.51 (d, 2H, *J* = 8.5 Hz), 7.99 (d, 2H, *J* = 8.1 Hz), 7.93 (d, 2H, *J* = 8.9 Hz), 7.81-7.78 (m, 2H), 7.67 -7.58 (m, 13H), 7.51 (t, 2H, *J* = 7.4 Hz), 7.48 (d, 2H, *J* = 8.8 Hz), 7.38-7.35 (m, 2H), 5.69 (t, 1H, *J* = 4.6 Hz), 4.85 (d, 2H, *J* = 15.1 Hz), 2.65-2.56 (m, 2H), 1.86-1.75 (m, 2H), 0.96-0.84 (m, 2H); ^{13}C NMR (150 MHz, DMSO-d_6) δ 162.65 (d, *J* = 4.9 Hz), 149.88, 135.21, 134.16 (d, *J* = 10.5 Hz),

131.35 (d, *J* = 52.4 Hz), 130.28 (d, *J* = 12.7 Hz), 129.11 (d, *J* = 20.3 Hz), 128.81, 127.46, 124.95, 123.55, 119.55, 118.96, 117.72, 116.85, 40.82, 33.82, 31.49 (d, *J* = 56.3 Hz), 30.24, 24.98. ^{31}P NMR (161 MHz, DMSO-d_6) δ 21.80. Anal. Calcd for $C_{44}H_{37}ClNO_2P$: C, 77.92; H, 5.50; Cl, 5.23; N, 2.07; P, 4.57. Found: C, 78.08; H, 5.63; Cl, 5.38; N, 1.89; P, 4.69. MS (ESI) *m/z* calcd for 677.2, found 642.3 $[M + H - Cl]^+$, 6652.5 $[M + Na - Cl]^+$. ESI-HRMS, *m/z*: $[M-Cl^-]^+$ calcd for $C_{44}H_{37}NO_2P$ 642.2556, found 642.2561.

2-((3-(14H-dibenzo[a,j]xanthen-14-yl)propyl)amino)-2-oxoethyl)(methyl)diphenylphosphonium chloride (**6d**). White solid; yield 85%; mp 199-200 °C. IR (KBr, cm^{-1}) 1458, 1515, 1668, 2880, 2925; ^{1}H NMR (600 MHz, DMSO-d_6) δ 8.64 (t, 1H, *J* = 5.8 Hz), 8.52 (d, 2H, *J* = 8.5 Hz), 7.98 (d, 2H, *J* = 8.1 Hz), 7.93 (d, 2H, *J* = 8.8 Hz), 7.84-7.78 (m, 4H), 7.74 (t, 2H, *J* = 7.7 Hz), 7.67 (t, 2H, *J* = 7.7 Hz), 7.61 -7.56 (m, 4H), 7.51 (t, 2H, *J* = 7.5 Hz), 7.47 (d, 2H, *J* = 8.8 Hz), 5.70 (t, 1H, *J* = 4.8 Hz), 4.29 (d, 2H, *J* = 15.2 Hz), 2.71-2.62 (m, 2H), 2.59 (d, 3H, *J* = 14.7 Hz), 1.91-1.80 (m, 2H), 1.01-0.89 (m, 2H); ^{13}C NMR (150 MHz, DMSO-d_6) δ 162.93 (d, *J* = 5.0 Hz), 149.88, 134.73, 132.62 (d, *J* = 10.3 Hz), 131.35 (d, *J* = 53.0 Hz), 130.03 (d, *J* = 12.7 Hz), 129.17, 129.02, 127.45, 124.95, 123.55, 121.20, 120.62, 117.71, 116.87, 56.52, 33.87, 30.70 (d, *J* = 57.3 Hz), 25.16, 19.05, 7.27 (d, *J* = 55.8 Hz). ^{31}P NMR (161 MHz, DMSO-d_6) δ 21.73. Anal. Calcd for $C_{39}H_{35}ClNO_2P$: C, 76.03; H, 5.73; Cl, 5.75; N, 2.27; P, 5.03. Found: C, 76.20; H, 5.88; Cl, 5.89; N, 2.12; P, 5.12.

(2-((2,2-Bis(6-hydroxybenzo[d][1,3]dioxol-5-yl)ethyl)amino)-2-oxoethyl)triphenylphosphonium chloride (**7a**). White solid; yield 47%; mp 178-181 °C. IR (KBr, cm^{-1}) 1439, 1485, 1504, 1668, 2890, 3206; ^{1}H NMR (600 MHz, DMSO-d_6) δ 9.22 (s, 2H), 8.40 (t, 1H, *J* = 5.3 Hz), 7.88-7.85 (m, 2H), 7.74-7.67 (m, 11H), 7.63-7.54 (m, 2H), 6.55 (s, 2H), 6.49 (s, 2H), 5.86 -5.83 (m, 4H), 4.88 (d, 2H, *J* = 15.1 Hz), 4.46 (t, 1H, *J* = 7.9 Hz), 3.51-3.41 (m, 2H); ^{13}C NMR (150 MHz, DMSO-d_6) δ 162.94 (d, *J* = 5.0 Hz), 150.11, 145.93, 139.88, 135.26, 134.16 (d, *J* = 10.5 Hz), 130.40 (d, *J* = 12.9 Hz), 120.41, 119.36 (d, *J* = 88.5 Hz), 108.26, 100.88, 98.21, 42.20, 37.44, 32.63 (d, *J* = 57.2 Hz). ^{31}P NMR (161 MHz, DMSO-d_6) δ 21.48. Anal. Calcd for $C_{36}H_{31}ClNO_7P$: C, 65.91; H, 4.76; Cl, 5.40; N, 2.13; P, 4.72. Found: C, 66.25; H, 4.71; Cl, 5.31; N, 1.98; P, 4.89. MS (ESI) *m/z* calcd for 655.1, found 620.5 $[M + H - Cl]^+$.

(2-((3,3-Bis(6-hydroxybenzo[d][1,3]dioxol-5-yl)propyl)amino)-2-oxoethyl)triphenylphosphonium chloride (**7b**). White solid; yield 61%; mp >250 °C. IR (KBr, cm^{-1}) 1439, 1485, 1503, 1558, 1678, 2894, 3066, 3218; ^{1}H NMR (600 MHz, DMSO-d_6) δ 9.07 (s, 2H), 8.83 (t, 1H, *J* = 5.6 Hz), 7.87-7.82 (m, 3H), 7.81-7.76 (m, 6H), 7.74-7.69 (m, 6H), 6.58 (s, 2H), 6.45 (s, 2H), 5.84 (d, 4H, *J* = 16.8 Hz), 4.97 (d, 2H, *J* = 15.1 Hz), 4.33 (t, 1H, *J* = 7.8 Hz), 2.87-2.77 (m, 2H), 1.81-1.67 (m, 2H); ^{13}C NMR (150 MHz, DMSO-d_6) δ 162.82 (d, *J* = 4.9 Hz), 149.68, 145.60, 139.98, 135.26, 134.28 (d, *J* = 10.8 Hz), 130.42 (d, *J* = 12.7 Hz), 122.55, 119.35 (d, *J* = 88.2 Hz), 108.08, 100.84, 98.08, 38.87, 34.82, 33.58, 31.75 (d, *J* = 56.1 Hz). ^{31}P NMR (161 MHz, DMSO-d_6) δ 22.08. Anal. Calcd for $C_{37}H_{33}ClNO_7P$: C, 66.32; H, 4.96; Cl, 5.29; N, 2.09; P, 4.62. Found: C, 66.37; H, 5.08; Cl, 5.21; N, 2.09; P, 4.50. MS (ESI) *m/z* calcd for 669.2, found 635.1 $[M + H - Cl]^+$.

1-(2-((2-(14H-dibenzo[a,j]xanthen-14-yl)ethyl)amino)-2-oxoethyl)pyridin-1-ium chloride (**8**). White solid; yield 78%; mp 156 °C. IR (KBr, cm^{-1}) 1435, 1488, 1515, 1682; ^{1}H NMR (600 MHz, DMSO-d_6) δ 8.84 (d, 2H, *J* = 6.0 Hz), 8.68 (t, 1H, *J* = 5.1 Hz), 7.64-7.51 (m, 3H), 8.08 (t, 2H, *J* = 7.0 Hz), 7.99 (d, 2H, *J* = 8.1 Hz), 7.94 (d, 2H, *J* = 8.9 Hz), 7.70 (t, 2H, *J* = 7.7 Hz), 7.53 (t, 2H, *J* = 7.4 Hz), 7.46 (d, 2H, *J* = 8.9 Hz), 5.80 (t, 1H, *J* = 5.0 Hz), 5.27 (s, 2H), 2.86-2.68 (m, 2H), 2.28-2.06 (m, 2H); ^{13}C NMR (150 MHz, DMSO-d_6) δ 164.23, 149.77, 146.50, 146.37, 131.46, 131.15, 129.20, 129.15, 127.78, 127.47, 124.99, 123.57, 117.62, 116.48, 61.78, 36.53, 35.61, 28.87. Anal. Calcd for $C_{30}H_{25}ClN_2O_2$: C, 74.91; H, 5.24; Cl, 7.37; N, 5.82. Found: C, 75.16; H, 5.37; Cl, 7.31; N, 5.69. MS (ESI) *m/z* calcd for 480.2, found 446.1 $[M + H - Cl]^+$.

1-(2-((2,2-Bis(6-hydroxybenzo[d][1,3]dioxol-5-yl)ethyl)amino)-2-oxoethyl)pyridin-1-ium chloride (**9a**). White solid; yield 57%; mp 178-180 °C. IR (KBr, cm^{-1}) 1439, 1489, 1503, 1676, 3232; ^{1}H NMR (600 MHz, DMSO-d_6) δ 9.23 (s, 2H), 8.93 (d, 2H, J = 6.0 Hz), 8.64 (t, 1H, J = 7.9 Hz), 8.54 (t, 1H, J = 5.6 Hz), 8.15 (t, 2H, J = 7.0 Hz), 6.61 (s, 2H), 6.50 (s, 2H), 5.85 (d, 4H, J = 12.2 Hz), 5.36 (s, 2H), 4.67 (t, 1H, J = 7.9 Hz), 3.63-3.55 (m, 2H); ^{13}C NMR (150 MHz, DMSO-d_6) δ 164.63, 150.15, 146.54, 146.47, 145.91, 139.91, 127.94, 120.67, 108.32, 100.88, 98.18, 62.12, 42.55, 37.11. Anal. Calcd for $C_{23}H_{21}ClN_2O_7$: C, 58.42; H, 4.48; Cl, 7.50; N, 5.92. Found: C, 58.56; H, 4.71; Cl, 7.57; N, 6.07. MS (ESI) *m/z* calcd for 472.1, found 438.6 $[M + H - Cl]^+$, 460.5 $[M + Na - Cl]^+$.

1-(2-((3,3-Bis(6-hydroxybenzo[d][1,3]dioxol-5-yl)propyl)amino)-2-oxoethyl)pyridin-1-ium chloride (**9b**). White solid; yield 79%; mp 159 °C. IR (KBr, cm^{-1}) 1488, 1503, 1684; ^{1}H NMR (600 MHz, DMSO-d_6) δ 9.12 (s, 2H), 8.99 (d, 2H, J = 6.0 Hz), 8.77 (t, 1H, J = 5.5 Hz), 8.65 (t, 1H, J = 7.9 Hz), 8.18 (t, 2H, J = 7.0 Hz), 6.68 (s, 2H), 6.47 (s, 2H), 5.85 (d, 4H, J = 78 Hz), 5.45 (s, 2H), 4.44 (t, 1H, J = 7.8 Hz), 3.12-2.95 (m, 2H), 2.14-2.01 (m, 2H); ^{13}C NMR (150 MHz, DMSO-d_6) δ 164.38, 149.72, 146.65, 146.46, 145.58, 139.99, 127.91, 122.59, 108.06, 100.81, 98.12, 62.03, 38.92, 34.89, 33.65. Anal. Calcd for $C_{24}H_{23}ClN_2O_7$: C, 59.20; H, 4.76; Cl, 7.28; N, 5.75. Found: C, 59.27; H, 4.91; Cl, 7.43; N, 5.61.

General Experimental Procedure for the Synthesis of 10. Acetal **2** (1.1 g, 6 mmol, 1 equiv) was added to a solution of benzene-1,2-diamine (0.65 g, 6 mmol, 1 equiv), sulfur (0.84 g) in dry DMF (6 mL) at a temperature of 40–50 °C for 10 h. The solvent was eliminated under reduced pressure. The residue was washed with 20 mL of ethanol dried in vacuo (10 torr, 4 h, 20 °C).

N-(2,2-dimethoxyethyl)-1H-benzo[d]imidazole-2-carboxamide (**10a**). Yellow oil; yield 50%. ^{1}H NMR (600 MHz, DMSO-d_6) δ 13.25 (s, 1H), 8.75 (t, 1H, J = 6.1 Hz), 7.79-7.67 (m, 1H), 7.61-7.49 (m, 1H), 7.41-7.22 (m, 2H), 4.62 (t, 1H, J = 5.5 Hz), 4.45 (t, 2H, J = 5.8 Hz), 3.31 (s, 6H); ^{13}C NMR (150 MHz, DMSO-d_6) δ 159.28, 145.89, 142.94, 134.96, 124.57, 123.03, 120.34, 113.02, 101.93, 53.46, 40.87. Anal. Calcd for $C_{12}H_{15}N_3O_3$: C, 57.82; H, 6.07; N, 16.86. Found: C, C, 57.67; H, 5.89; N, 16.98.

N-(3,3-diethoxypropyl)-1H-benzo[d]imidazole-2-carboxamide (**10b**). Yellow oil; yield 65%. ^{1}H NMR (600 MHz, DMSO-d_6) δ 8.15 (s, 1H), 7.53-7.49 (m, 2H), 7.20-7.13 (m, 2H), 4.54 (t, 1H, J = 5.3 Hz), 3.62-3.56 (m, 2H), 3.48-3.42 (s, 4H), 1.88-1.81 (m, 2H), 1.14 (t, 6H, J = 7.0 Hz); ^{13}C NMR (150 MHz, DMSO-d_6) δ 159.08, 145.72, 101.84, 61.76, 39.64, 35.46, 33.20, 15.37. Anal. Calcd for $C_{15}H_{21}N_3O_3$: C, 61.84; H, 7.27; N, 14.42. Found: C, 62.05; H, 7.51; N, 14.54.

General Experimental Procedure for the Synthesis of 11,12. Acetal **10** (2 mmol, 1 equiv) was added to a solution of phenol (4 mmol, 2 equiv), trifluoroacetic acid (1 ml) in dry chloroform (10 mL). The reaction mixture was stirred at room temperature for 96 hours. The solvent was eliminated under reduced pressure. The residue was washed with 10 mL of diethyl ether and dried in vacuo (10 torr, 10 h, 20 °C).

N-((14H-dibenzo[a,j]xanthen-14-yl)methyl)-1H-benzo[d]imidazole-2-carboxamide (**11a**). White solid; yield 54%; mp 216 °C. IR (KBr, cm^{-1}) 1446, 1461, 1514, 1659; ^{1}H NMR (600 MHz, DMSO-d_6) δ 9.02 (t, 1H, J = 6.4 Hz), 8.69 (d, 2H, J = 8.5 Hz), 7.97-7.84 (m, 4H), 7.64-7.44 (m, 6H), 7.39 (t, 2H, J = 7.4 Hz), 7.30-7.22 (m, 2H), 5.96 (t, 1H, J = 6.3 Hz), 3.69 (t, 2H, J = 6.3 Hz); ^{13}C NMR (150 MHz, DMSO-d_6) δ 159.48, 150.25, 145.80, 132.12, 131.02, 129.25, 128.85, 127.01, 124.75, 123.46, 117.71, 116.31, 45.58, 31.18. Anal. Calcd for $C_{30}H_{21}N_3O_2$: C, 79.10; H, 4.65; N, 9.22. Found: C, 79.19; H, 4.76; N, 9.36. MS (ESI) *m/z* calcd for 455.2, found 456.1 $[M + H]^+$, 478.3 $[M + Na]^+$.

N-((2,12-dihydroxy-14H-dibenzo[a,j]xanthen-14-yl)methyl)-1H-benzo[d]imidazole-2-carboxamide (**11b**). White solid; yield 74%; mp 224-225 °C. IR (KBr, cm^{-1}) 1452, 1480, 1519, 1669, 3085; ^{1}H NMR (600 MHz, acetone-d_6) δ 8.43 (s, 2H), 7.87 (s, 2H), 7.76-7.69 (m, 4H), 7.65-7.60 (m, 2H), 7.38-7.32 (m, 2H), 7.17 (d, 2H, *J* = 8.8 Hz), 7.06-6.99 (m, 2H), 5.69 (t, 1H, *J* = 5.7 Hz), 3.98-3.88 (m, 2H); ^{13}C NMR (150 MHz, acetone-d_6) δ 158.24, 156.42, 150.88, 144.67, 137.51, 133.74, 130.19, 128.64, 125.83, 123.99, 116.21, 116.04, 114.21, 113.73, 105.04, 44.24, 31.45. Anal. Calcd for $C_{30}H_{21}N_3O_4$: C, 73.91; H, 4.34; N, 8.62. Found: C, 74.12; H, 4.43; N, 8.60. MS (ESI) *m/z* calcd for 487.5, found 488.5 $[M + H]^+$.

N-(2-(2,12-dihydroxy-14H-dibenzo[a,j]xanthen-14-yl)ethyl)-1H-benzo[d]imidazole-2-carboxamide (**11c**). White solid; yield 53%; mp 212-214 °C. IR (KBr, cm^{-1}) 1439, 1471, 1732, 3009; ^{1}H NMR (600 MHz, DMSO-d_6) δ 8.69 (t, 1H, *J* = 5.4 Hz), 7.83 (d, 2H, *J* = 9.0 Hz), 7.78 (d, 2H, *J* = 8.9 Hz), 7.62 (s, 2H), 7.57-7.52 (m, 2H), 7.28-7.23 (m, 2H), 7.20 (d, 2H, *J* = 8.7 Hz), 7.11 (d, 2H, *J* = 8.7 Hz), 5.37-5.30 (m, 1H), 3.06-2.92 (m, 2H), 2.37-2.26 (m, 2H); ^{13}C NMR (150 MHz, DMSO-d_6) δ 159.04, 158.51, 157.03, 150.24, 145.76, 138.36, 133.22, 130.82, 128.89, 125.61, 123.81, 117.33, 117.06, 116.47, 114.47, 114.25, 105.20, 36.36, 34.31, 29.47. Anal. Calcd for $C_{31}H_{23}N_3O_4$: C, 74.24; H, 4.62; N, 8.38. Found: C, 74.24; H, 4.76; N, 8.39.

N-(2,2-bis(6-hydroxybenzo[d][1,3]dioxol-5-yl)ethyl)-1H-benzo[d]imidazole-2-carboxamide (**12a**). White solid; yield 54%; mp 216-217 °C. ^{1}H NMR (600 MHz, DMSO-d_6) δ 9.07 (s, 2H), 8.46 (t, 1H, *J* = 5.7 Hz), 7.59-7.55 (m, 2H), 7.27-7.23 (m, 2H), 6.73 (s, 2H), 6.37 (s, 2H), 5.85-5.80 (m, 4H), 4.82 (t, 1H, *J* = 8.0 Hz), 3.82-3.77 (m, 2H); ^{13}C NMR (150 MHz, DMSO-d_6) δ 158.89, 150.04, 145.98, 140.04, 123.76, 120.69, 108.52, 100.90, 98.11, 42.16, 37.57. Anal. Calcd for $C_{24}H_{19}N_3O_7$: C, 62.47; H, 4.15; N, 9.11. Found: C, 62.61; H, 4.34; N, 8.91.

N-(2,2-bis(4-hydroxy-2-oxo-2H-chromen-3-yl)ethyl)-1H-benzo[d]imidazole-2-carboxamide (**12b**). White solid; yield 66%; mp 263-265 °C. ^{1}H NMR (600 MHz, DMSO-d_6) δ 8.46 (s, 1H), 7.93-7.87 (m, 2H), 7.62-7.51 (m, 2H), 7.37-7.25 (m, 4H), 5.58 (t, 1H, *J* = 7.3 Hz), 4.11 (s, 2H), 3.25-3.16 (m, 1H), 3.06-2.96 (m, 1H); ^{13}C NMR (150 MHz, DMSO-d_6) δ 166.17, 163.96, 162.83, 162.37, 162.08, 153.19, 152.98, 133.22, 132.76, 124.88, 124.72, 124.04, 117.36, 117.23, 116.74, 105.89, 103.03, 45.38, 43.62, 28.28. Anal. Calcd for $C_{28}H_{19}N_3O_7$: C, 66.01; H, 3.76; N, 8.25. Found: C, 65.87; H, 3.91; N, 8.16. MS (ESI) *m/z* calcd for 509.3, found 510.5 $[M + H]^+$.

N-(3,3-bis(4-hydroxy-6-methyl-2-oxo-2H-pyran-3-yl)propyl)-1H-benzo[d]imidazole-2-carboxamide (**12c**). White solid; yield 71%; mp 189-191 °C. ^{1}H NMR (600 MHz, DMSO-d_6) δ 8.73 (t, 1H, *J* = 6.0 Hz), 7.69-7.59 (m, 2H), 7.33-7.26 (m, 2H), 6.00 (s, 2H), 4.52 (t, 1H, *J* = 8.1 Hz), 3.30-3.19 (m, 2H), 2.31-2.21 (m, 2H), 2.14 (s, 6H); ^{13}C NMR (150 MHz, DMSO-d_6) δ 167.47, 166.14, 163.71, 160.82, 158.84, 146.09, 138.77, 123.73, 116.59, 102.70, 101.51, 100.62, 88.58, 38.37, 30.21, 28.60, 19.50. Anal. Calcd for $C_{23}H_{21}N_3O_7$: C, 61.19; H, 4.69; N, 9.31. Found: C, 61.31; H, 4.84; N, 9.25. MS (ESI) *m/z* calcd for 451.1, found 452.7 $[M + H]^+$.

Copies of ^{1}H, ^{13}C NMR spectra

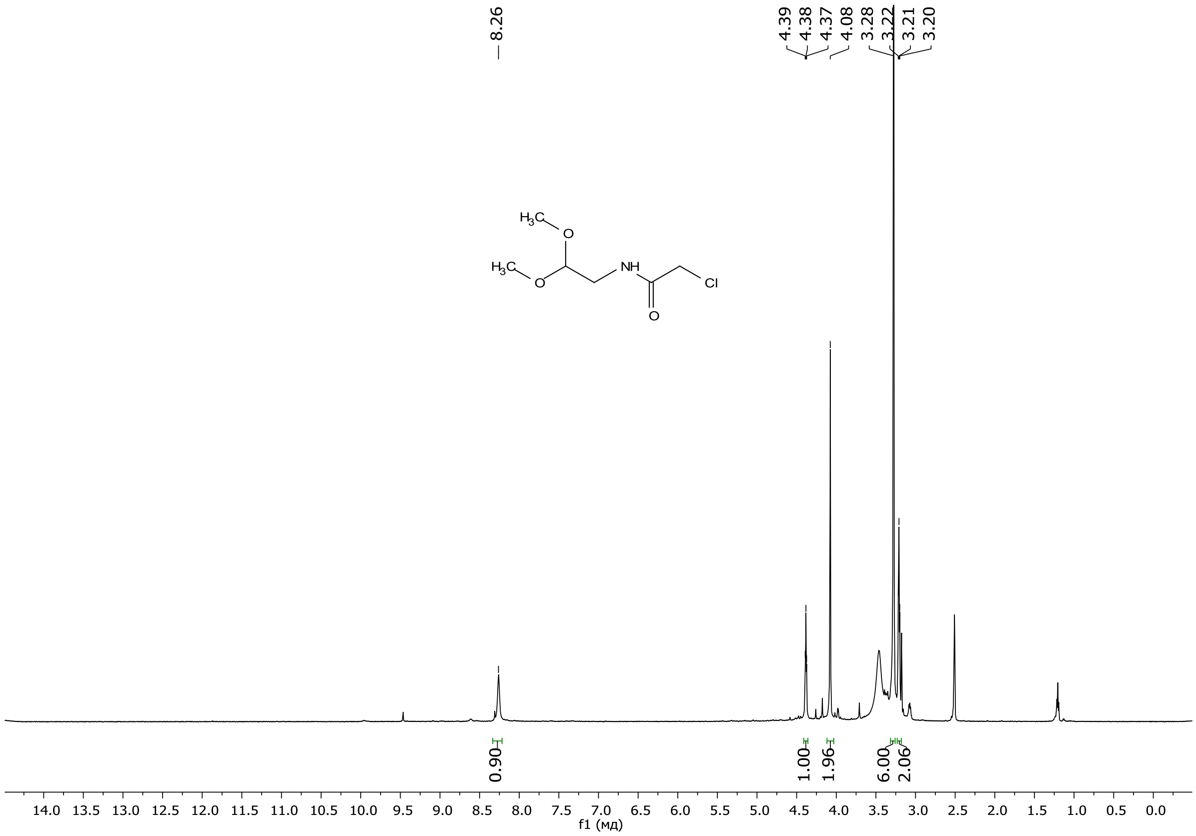

Figure S3. ^{1}H NMR (DMSO-d_6, 600 MHz) spectrum of the compound **2a**

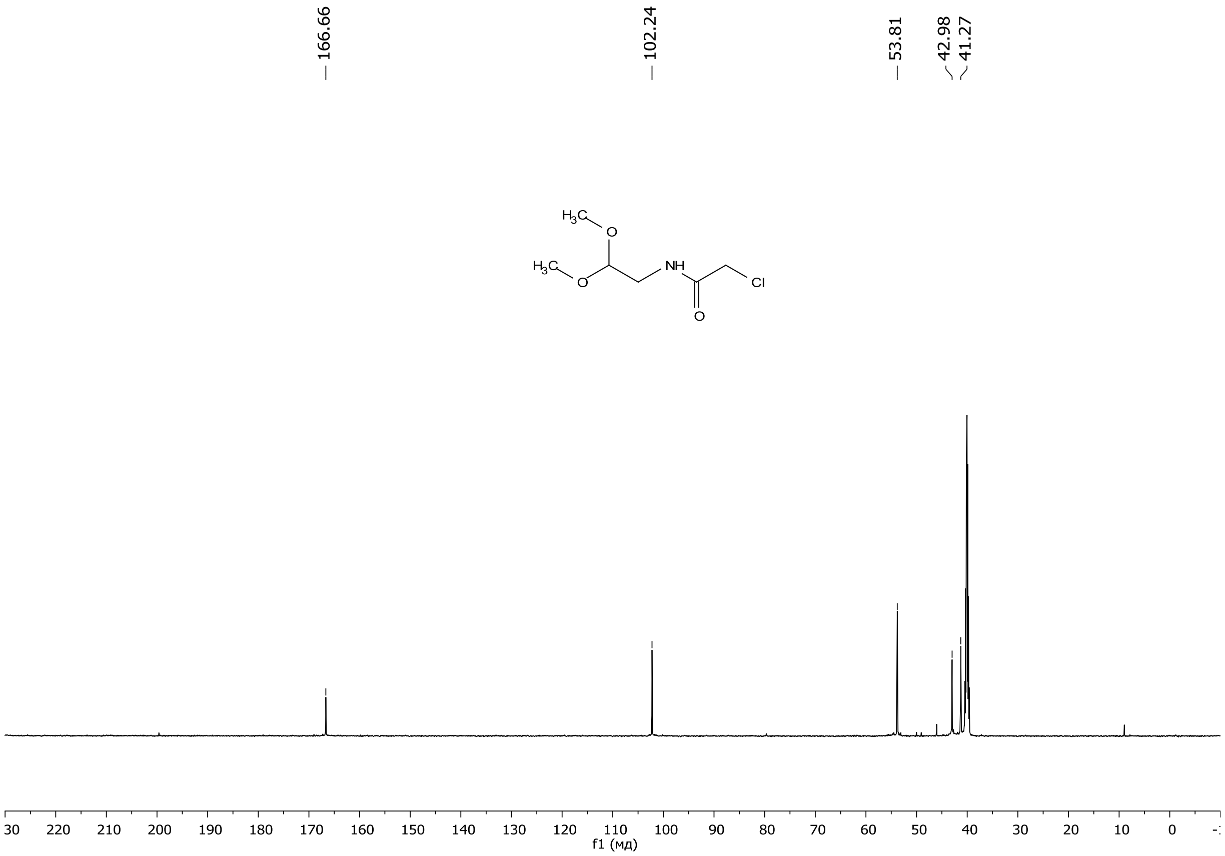

Figure S4. $^{13}C\{^{1}H\}$ NMR (DMSO-d_6, 150 MHz) spectrum of the compound **2a**

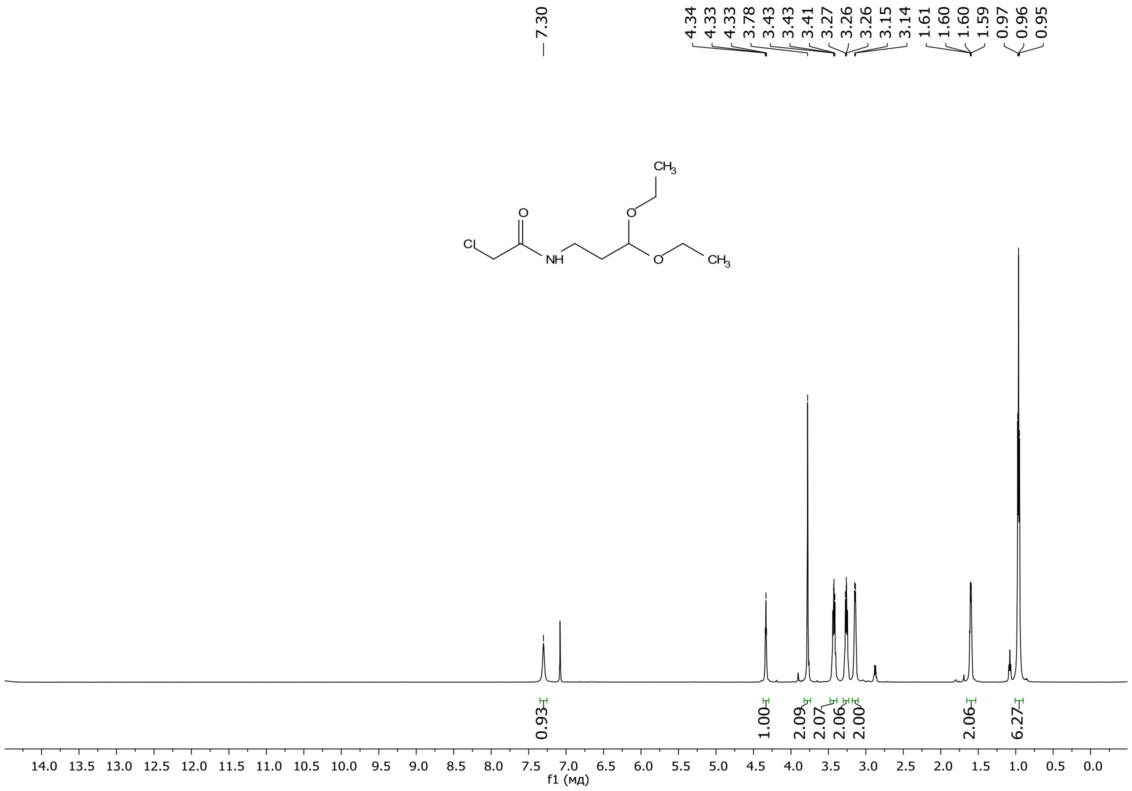

Figure S5. ^{1}H NMR ($CDCl_3$, 600 MHz) spectrum of the compound **2b**

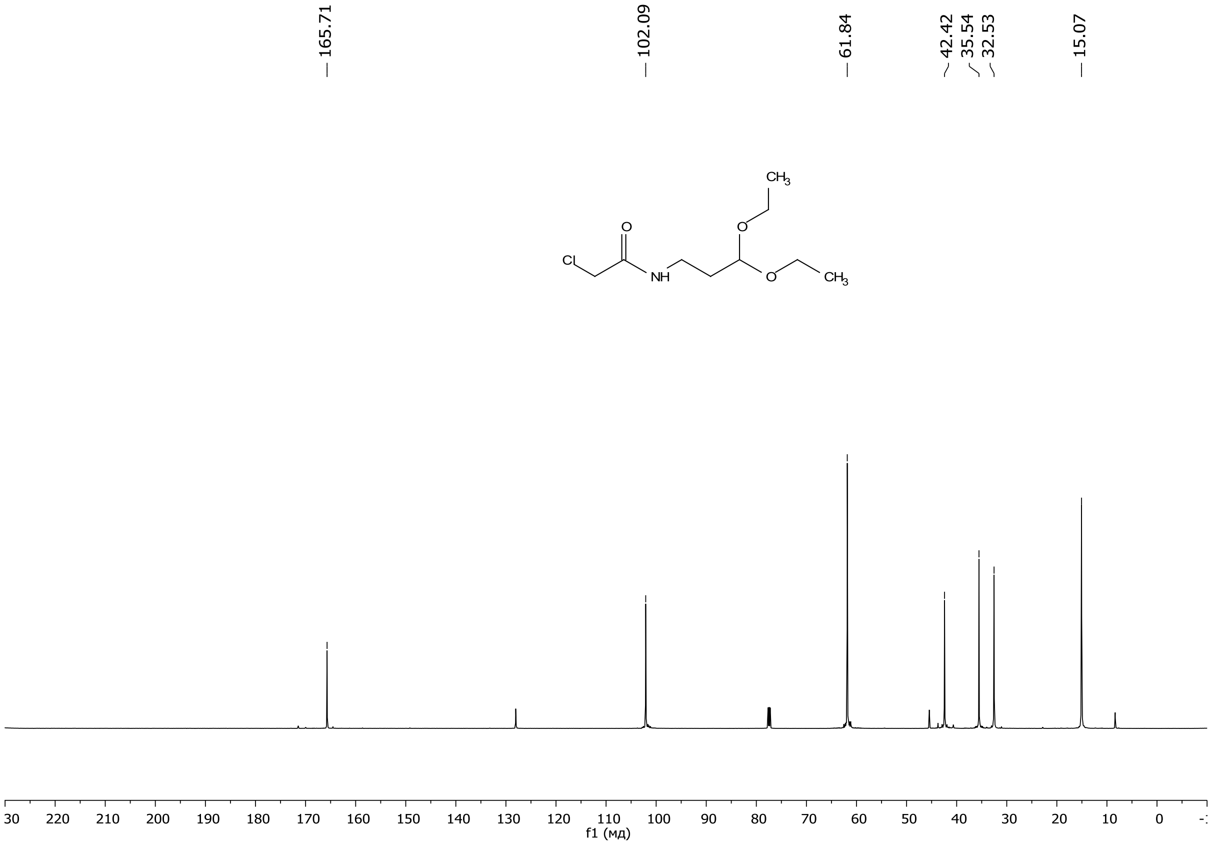

Figure S6. $^{13}C\{^{1}H\}$ NMR ($CDCl_3$, 150 MHz) spectrum of the compound **2b**

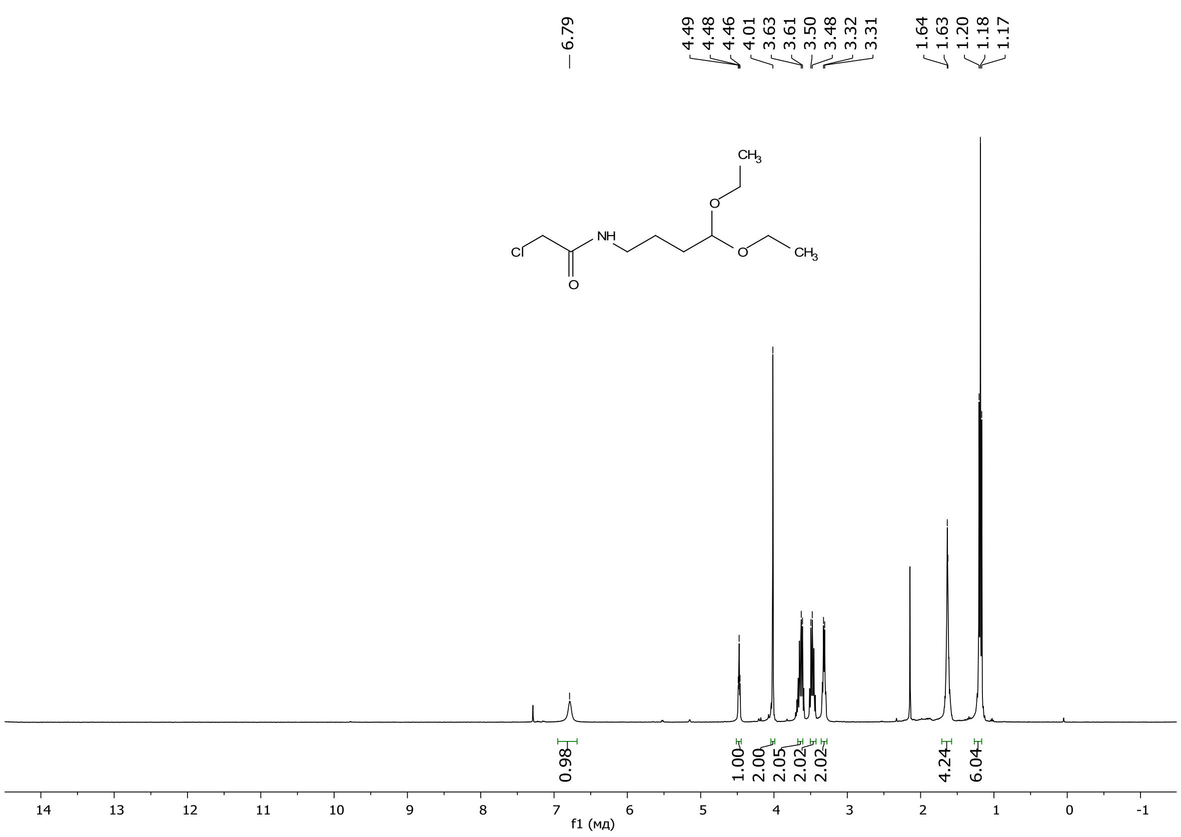

Figure S7. ^{1}H NMR ($CDCl_3$, 600 MHz) spectrum of the compound **2c**

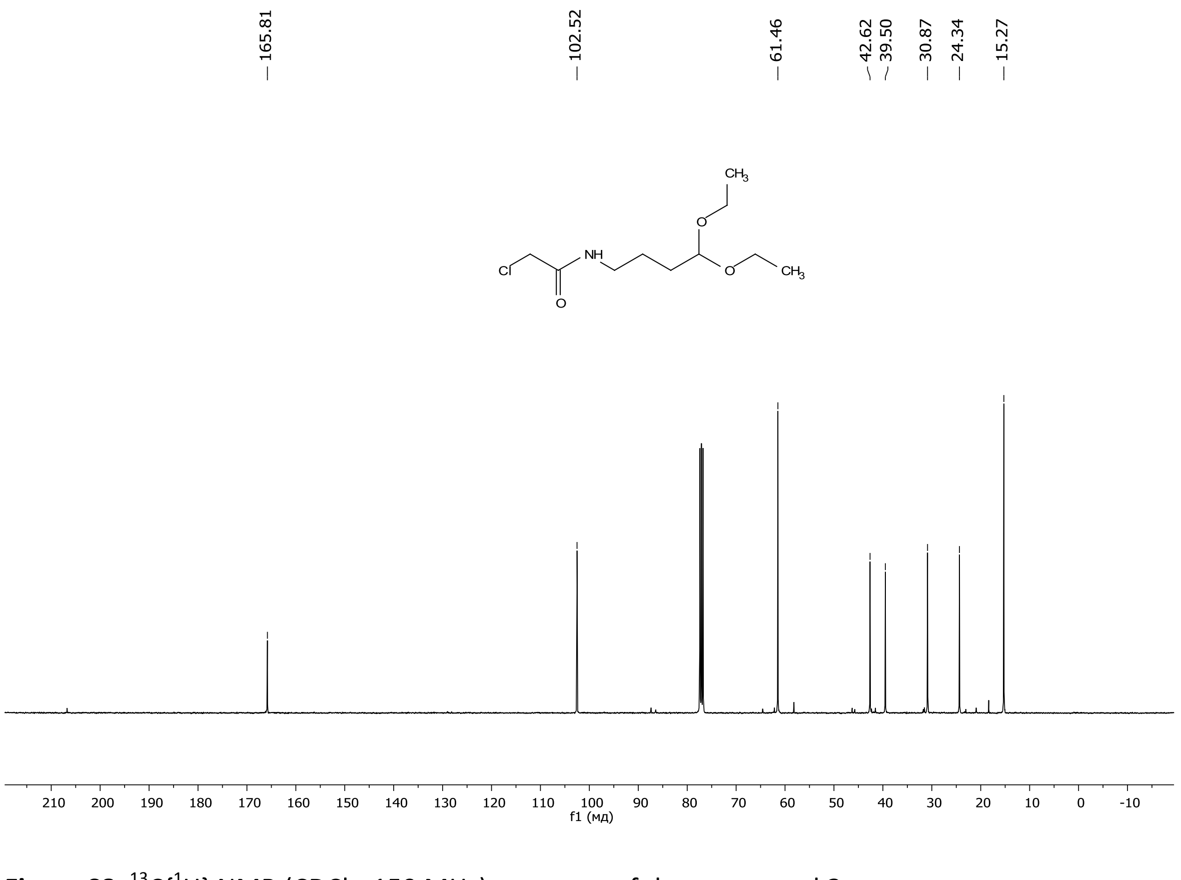

Figure S8. $^{13}C\{^{1}H\}$ NMR ($CDCl_3$, 150 MHz) spectrum of the compound **2c**

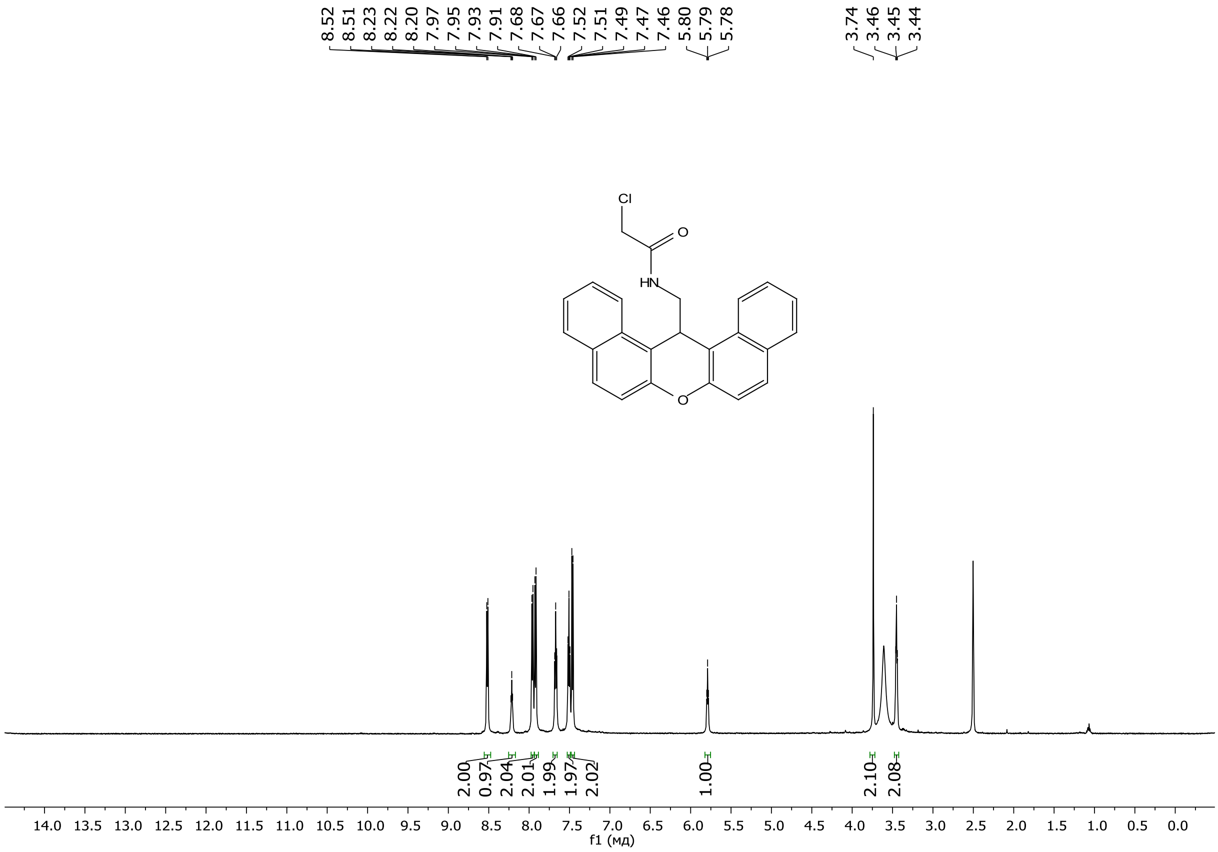

Figure S9. ^{1}H NMR (DMSO-d_6, 600 MHz) spectrum of the compound **3a**

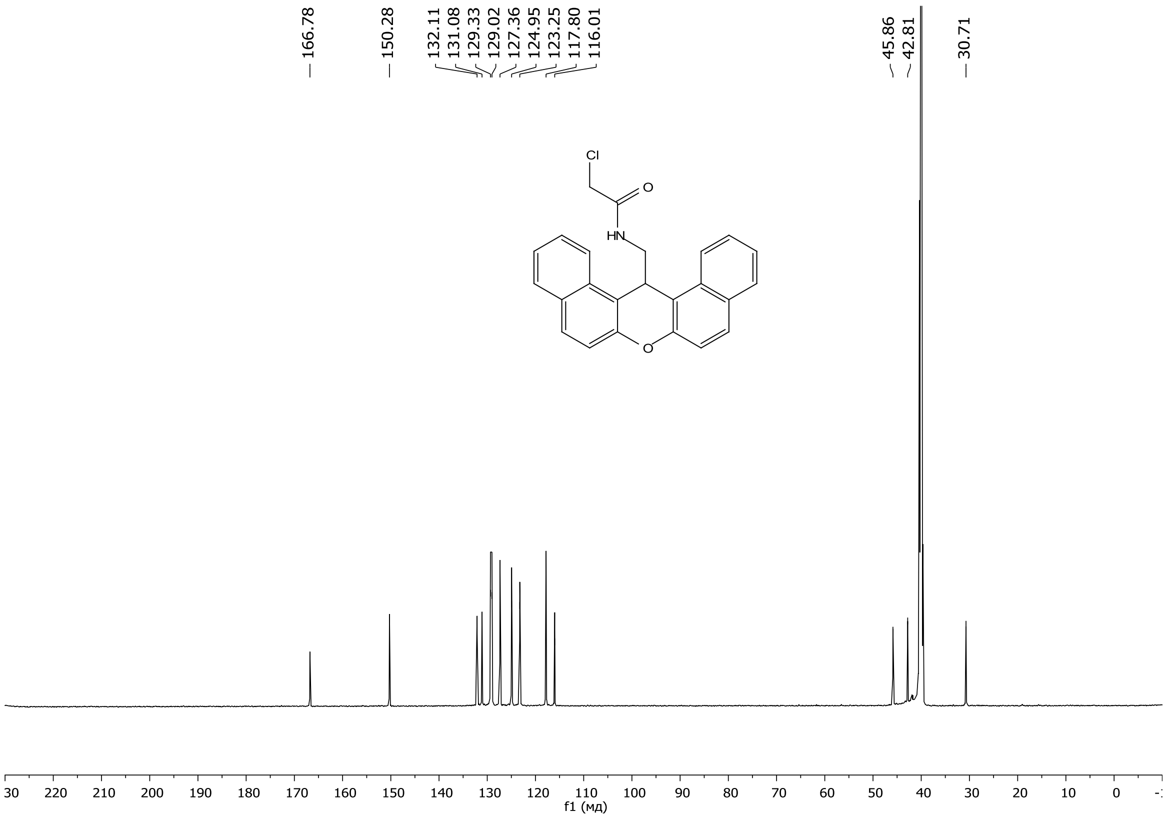

Figure S10. ^{13}C{^{1}H} NMR (DMSO-d_6, 150 MHz) spectrum of the compound **3a**

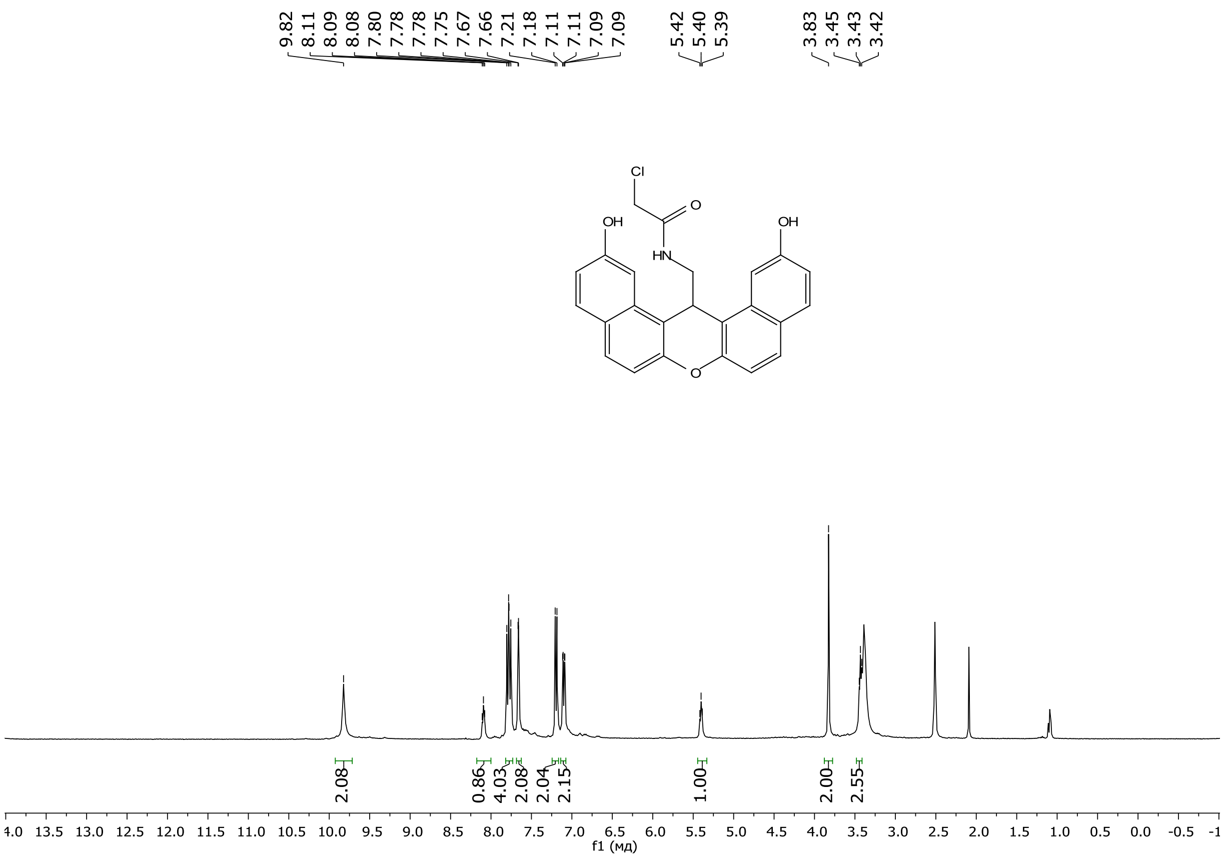

Figure S11. ^{1}H NMR (DMSO-d_6, 600 MHz) spectrum of the compound **3b**

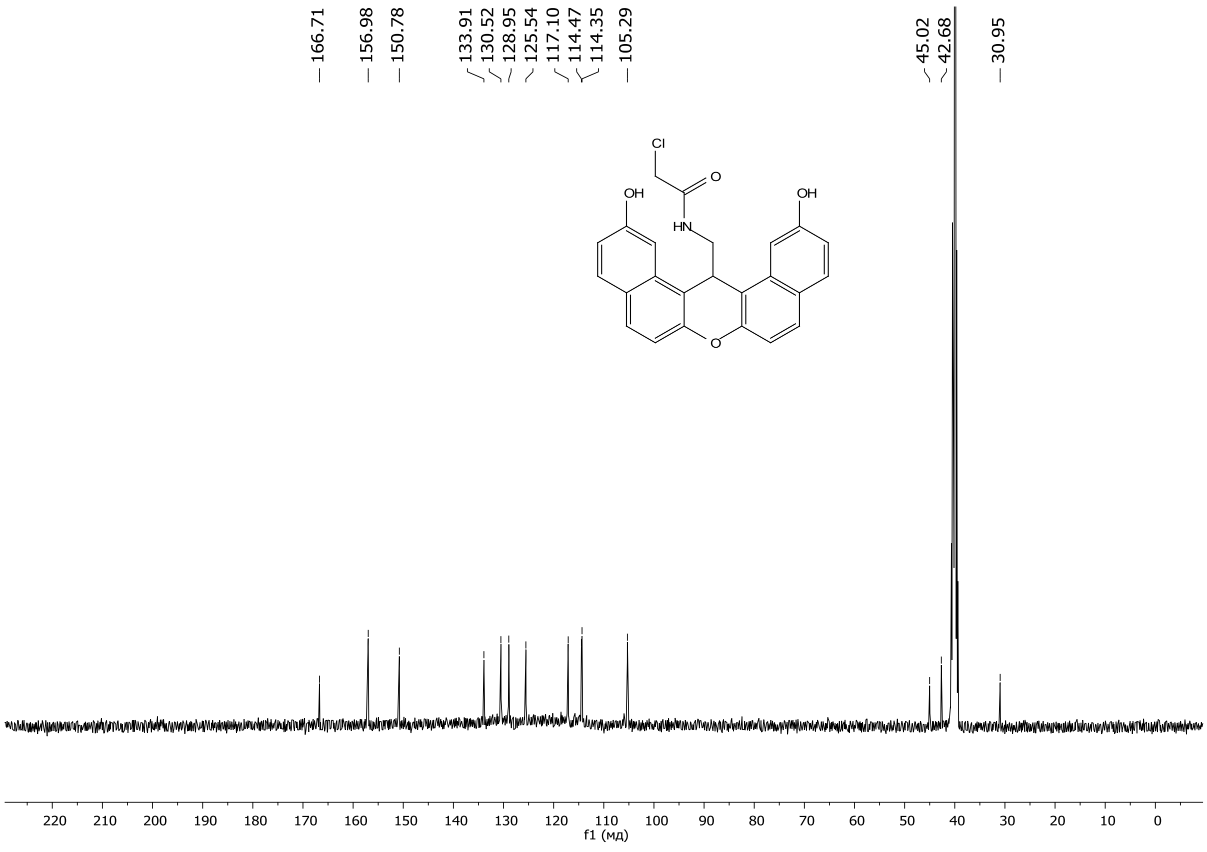

Figure S12. $^{13}C\{^{1}H\}$ NMR (DMSO-d_6, 150 MHz) spectrum of the compound **3b**

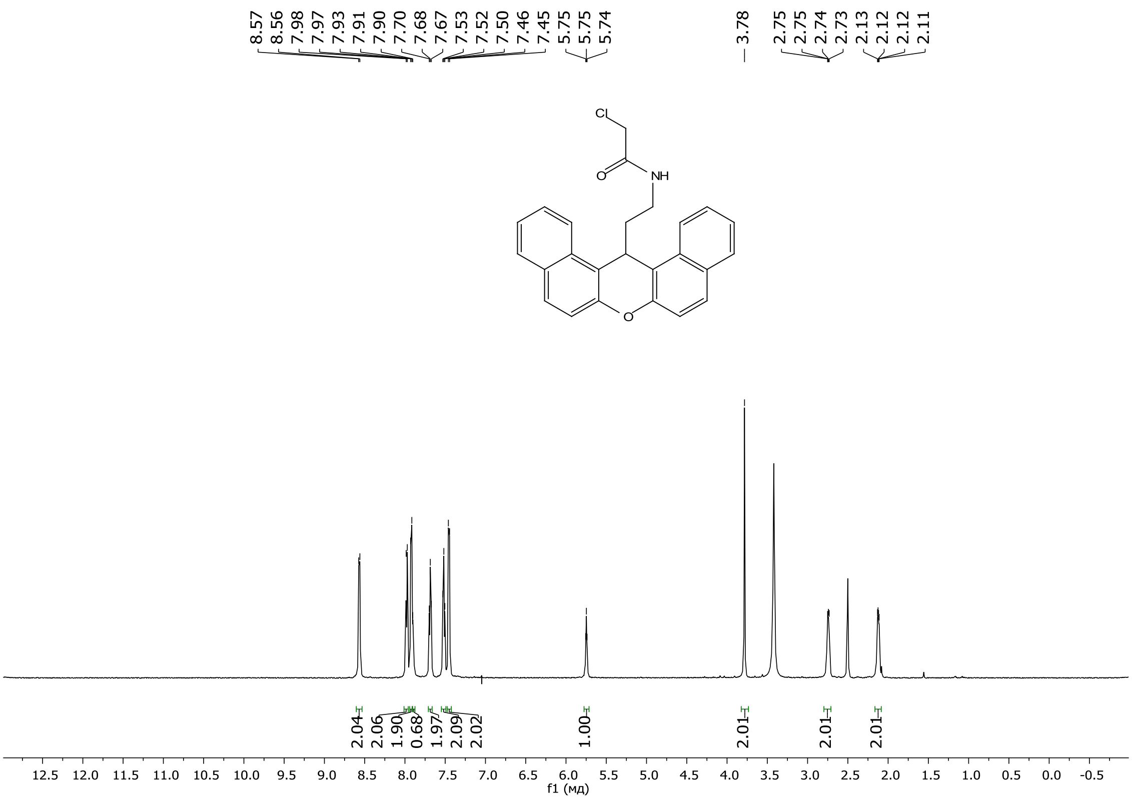

Figure S13. ^{1}H NMR (DMSO-d_6, 600 MHz) spectrum of the compound **3c**

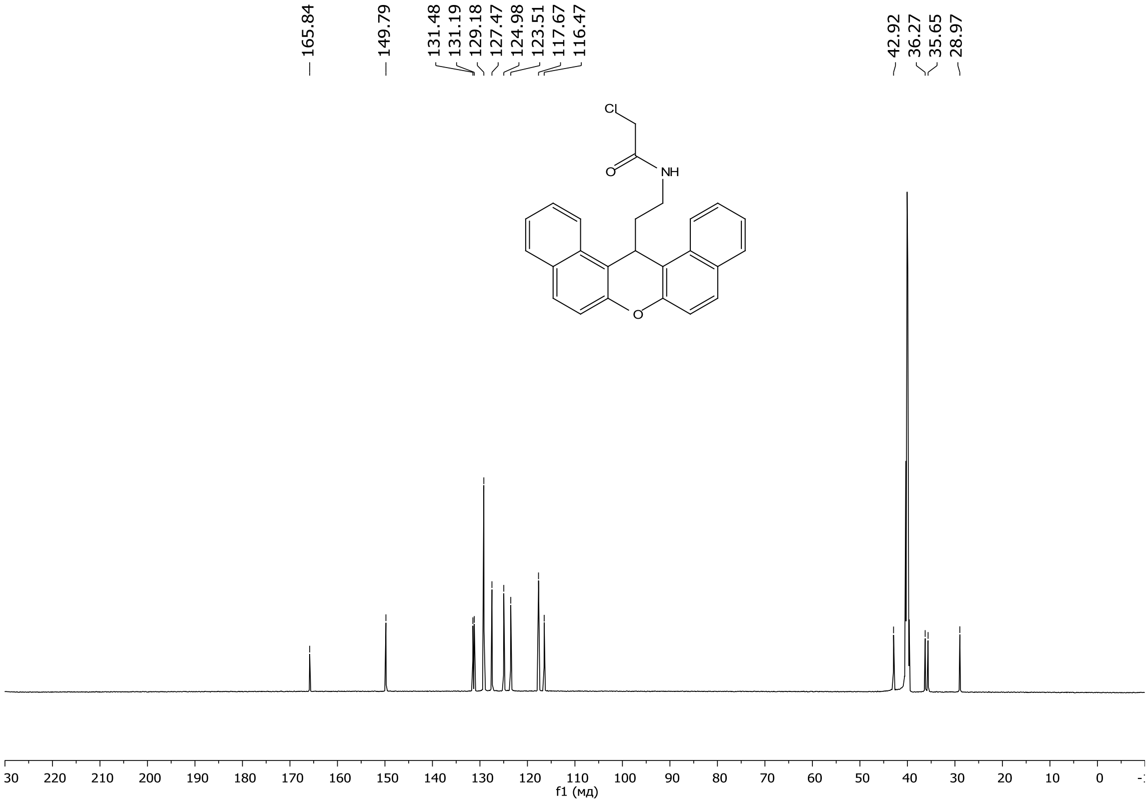

Figure S14. $^{13}C\{^{1}H\}$ NMR (DMSO-d_6, 150 MHz) spectrum of the compound **3c**

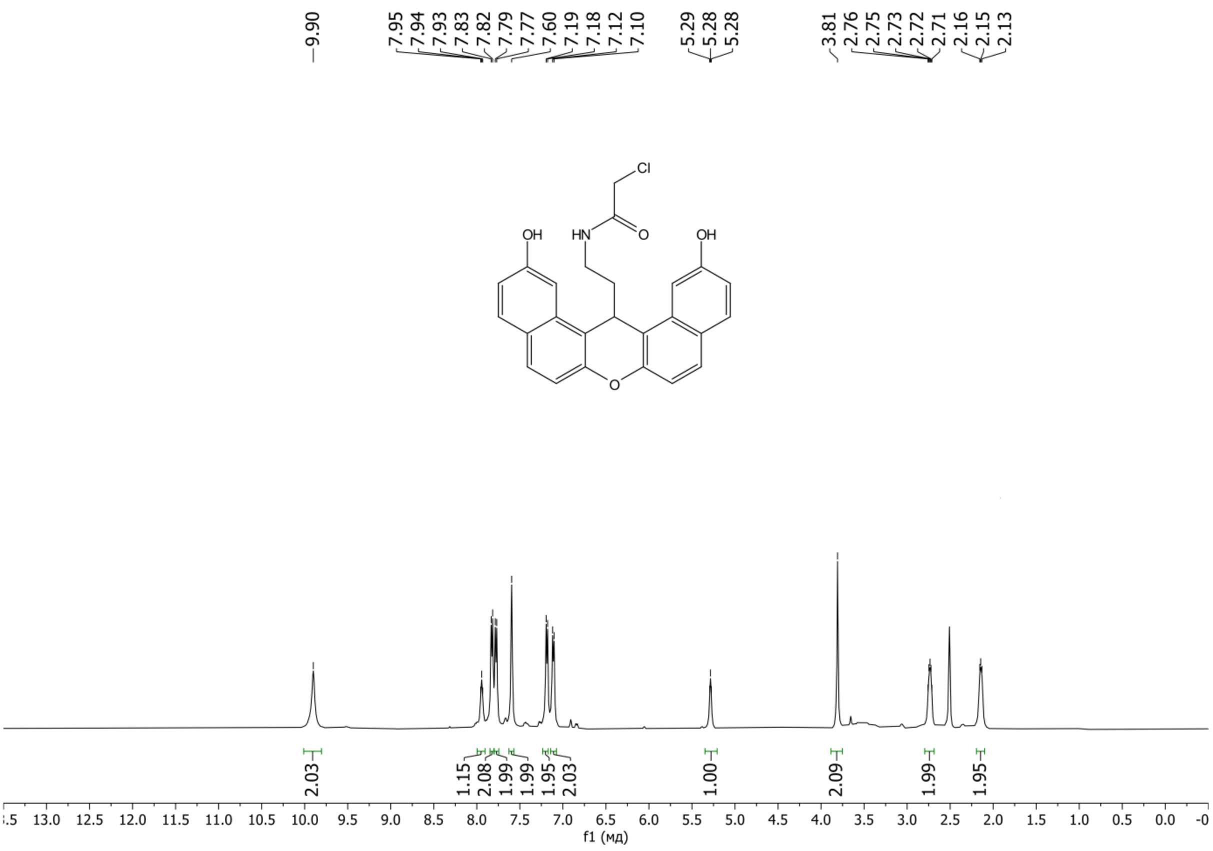

Figure S15. ^{1}H NMR (DMSO-d_6, 600 MHz) spectrum of the compound **3d**

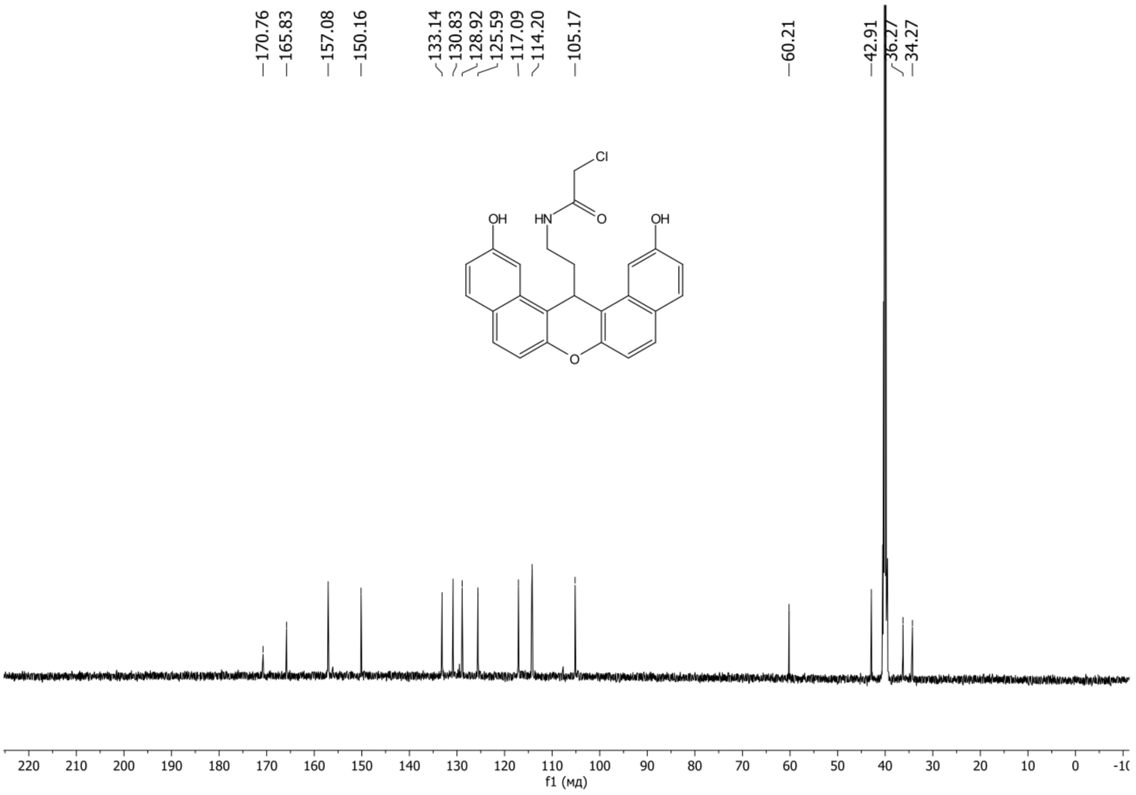

Figure S16. ^{13}C{^{1}H} NMR (DMSO-d_6, 150 MHz) spectrum of the compound **3d**

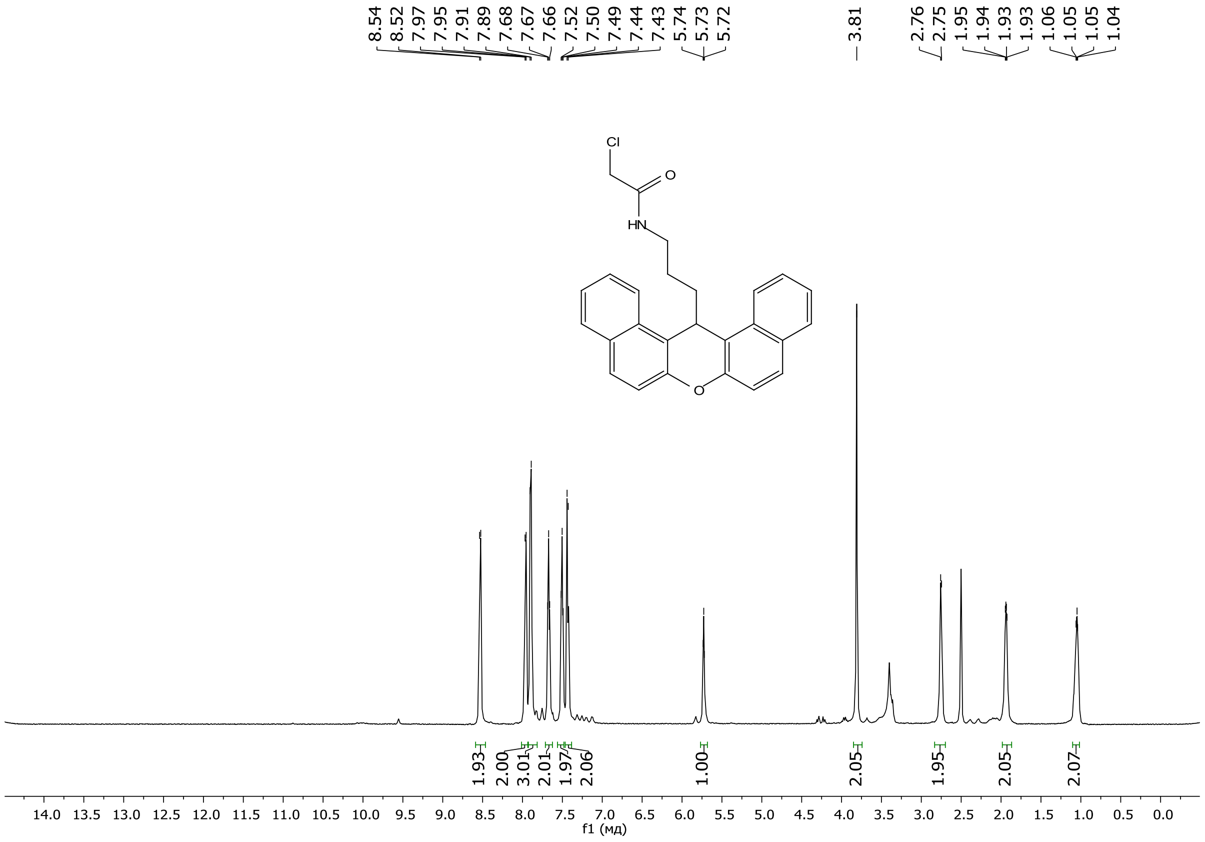

Figure S17. ^{1}H NMR (DMSO-d_6, 600 MHz) spectrum of the compound **3e**

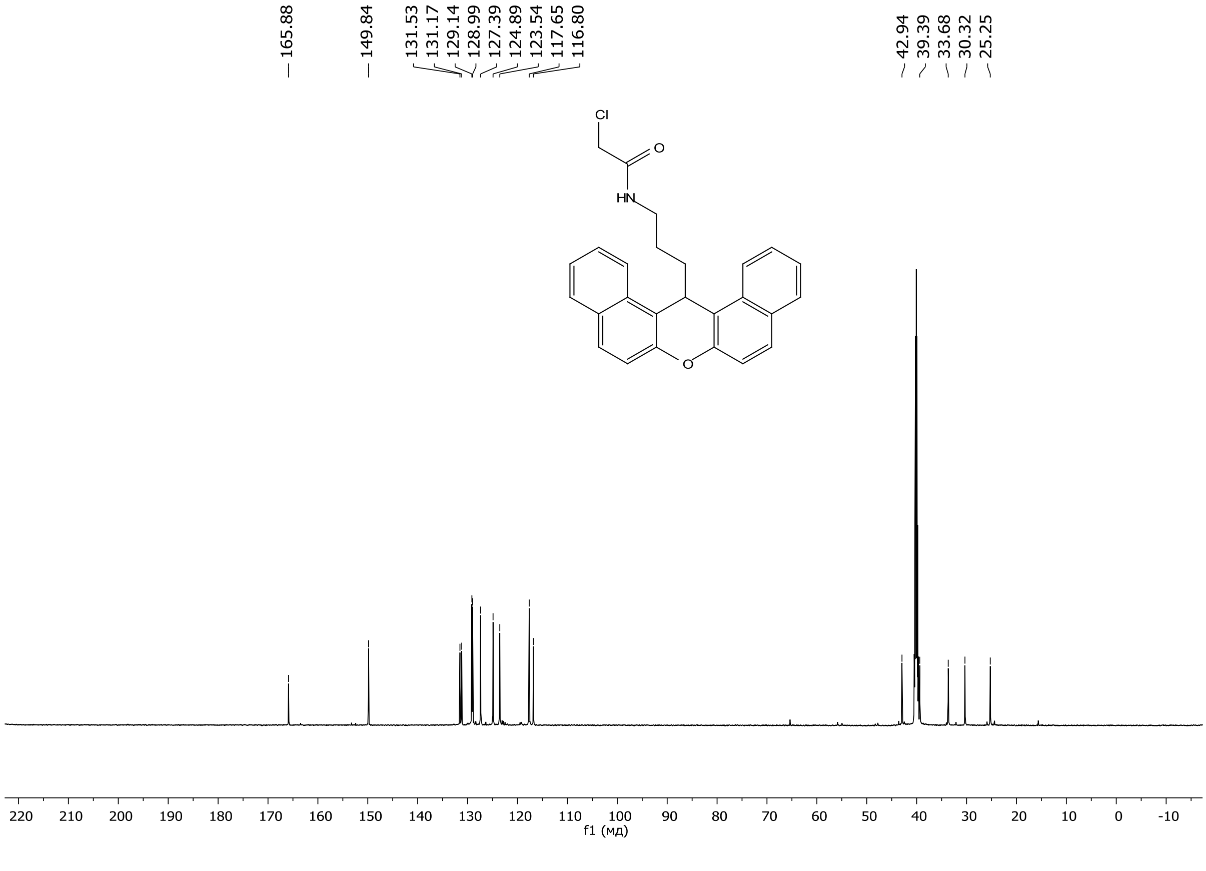

Figure S18. $^{13}C\{^1H\}$ NMR (DMSO-d_6, 150 MHz) spectrum of the compound **3e**

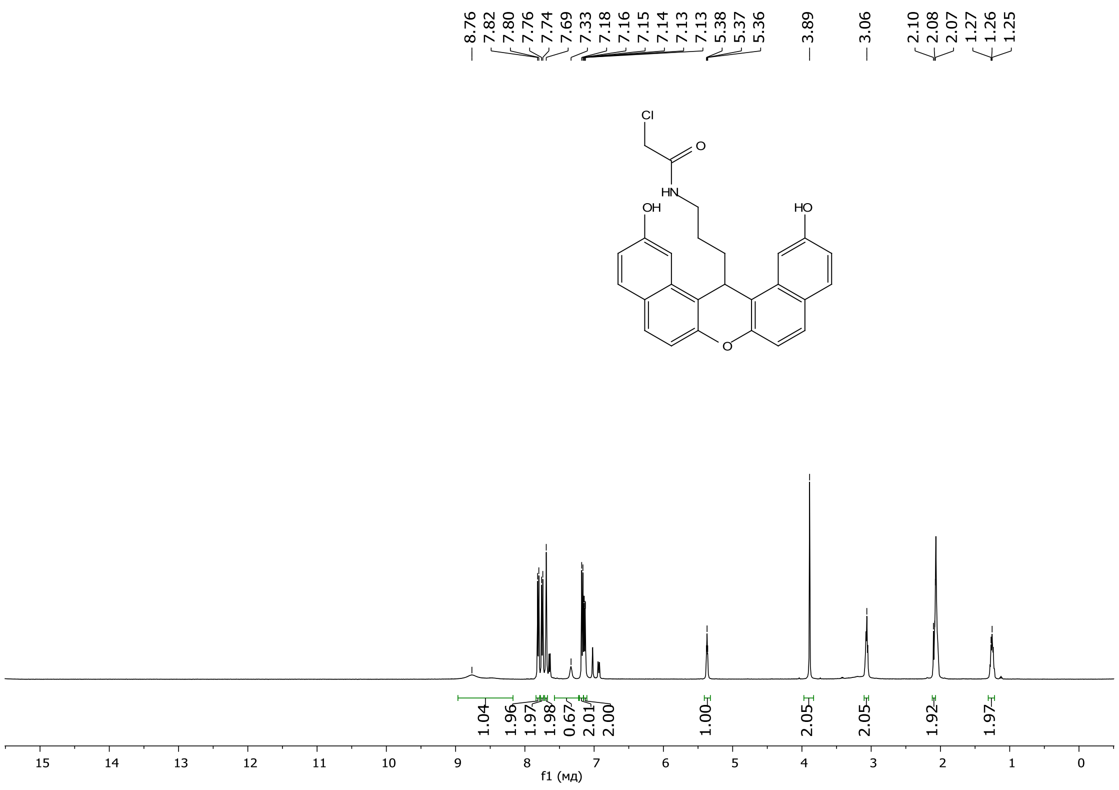

Figure S19. ^{1}H NMR (Acetone-d_6, 600 MHz) spectrum of the compound **3f**

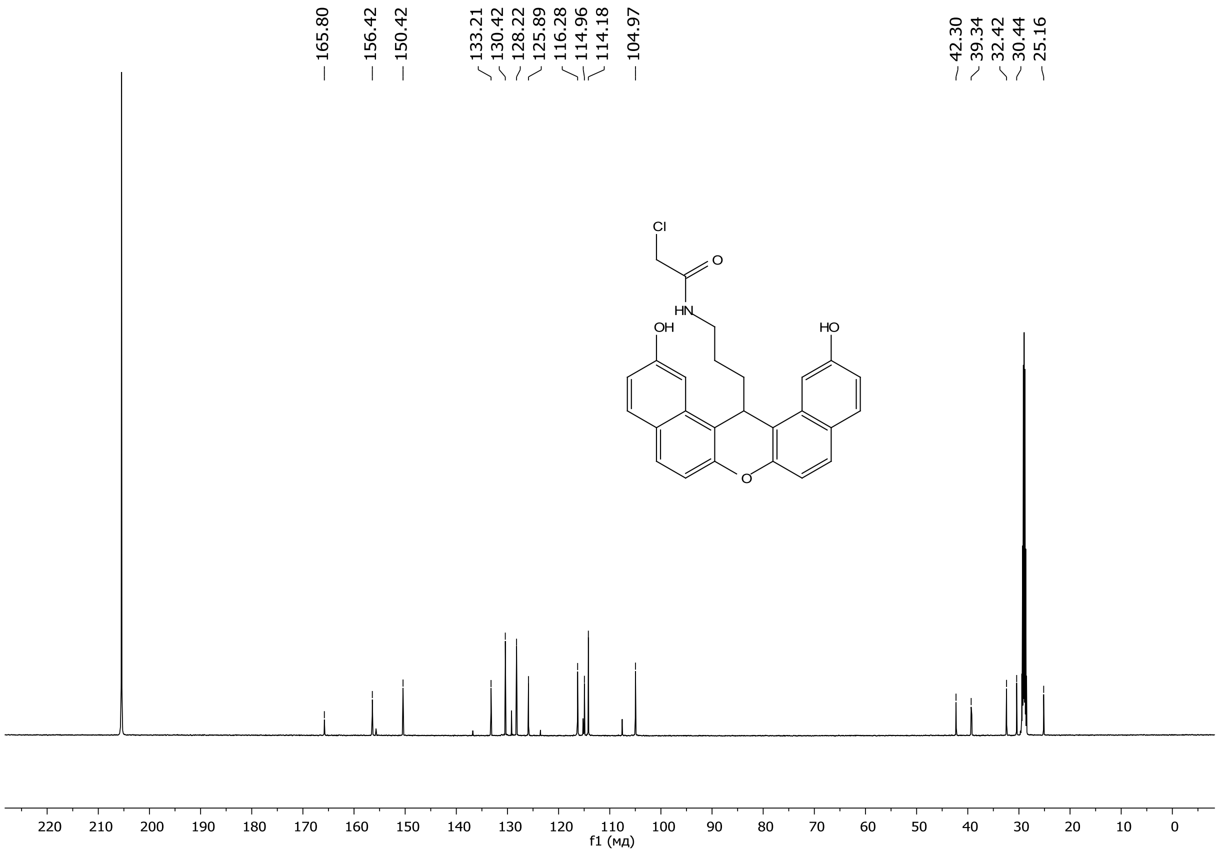

Figure S20. ^{13}C{^{1}H} NMR (Acetone-d_6, 150 MHz) spectrum of the compound **3f**

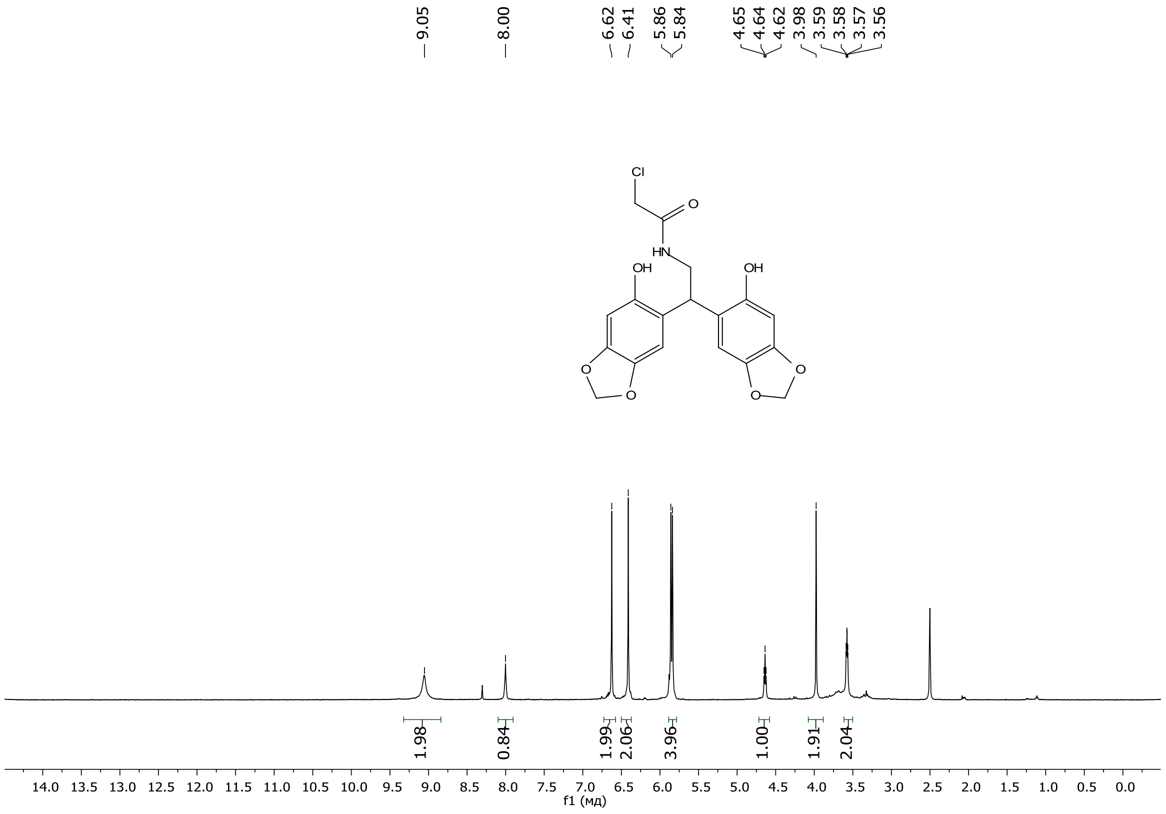

Figure S21. ^{1}H NMR (DMSO-d_6, 600 MHz) spectrum of the compound **4a**

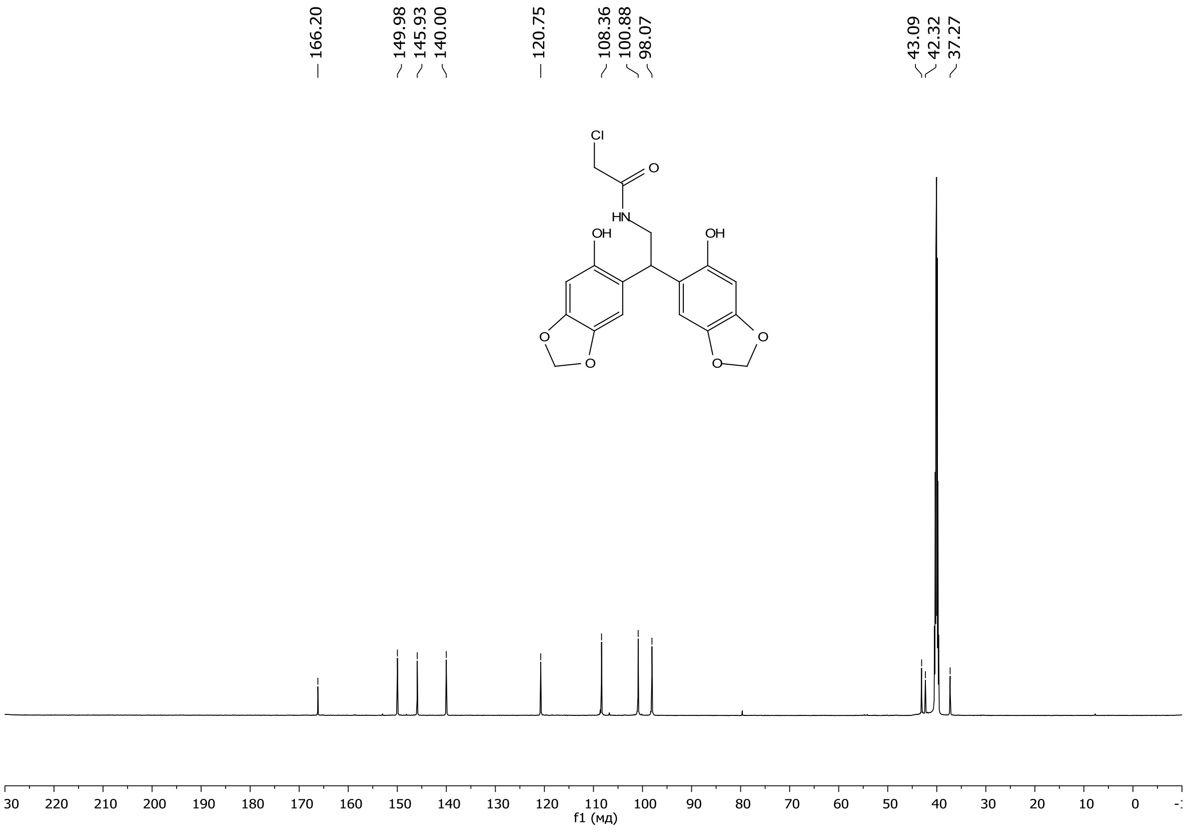

Figure S22. ^{13}C{^{1}H} NMR (DMSO-d_6, 150 MHz) spectrum of the compound **4a**

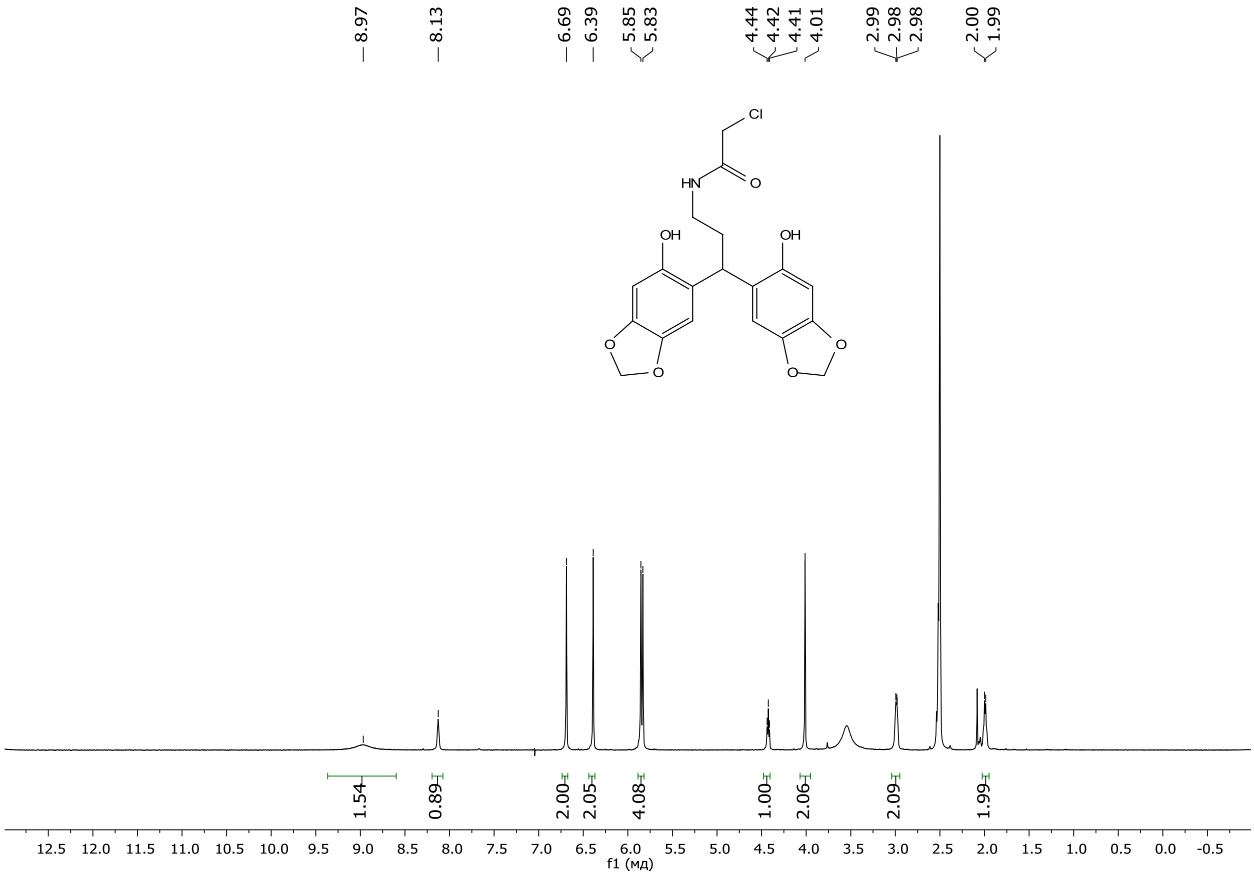

Figure S23. ^{1}H NMR (DMSO-d_6, 600 MHz) spectrum of the compound **4b**

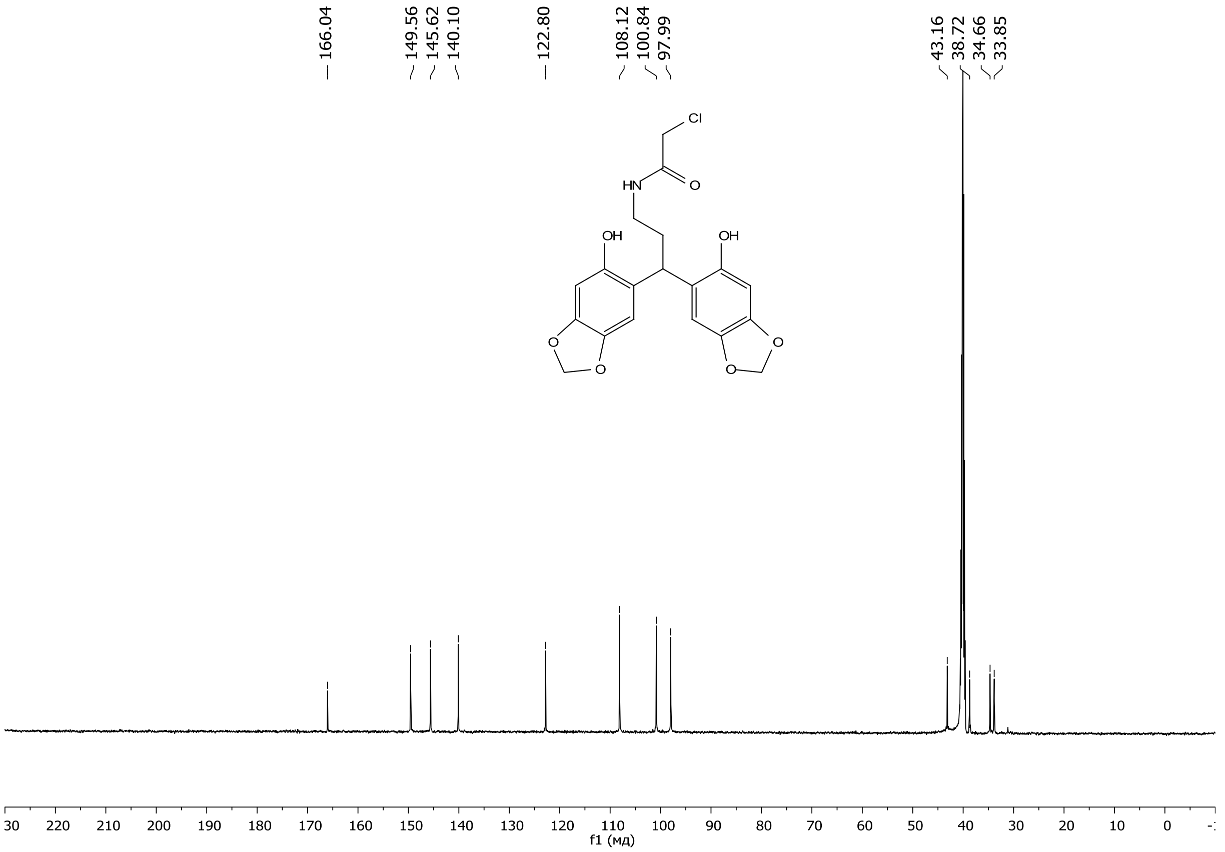

Figure S24. ^{13}C{^{1}H} NMR (DMSO-d_6, 150 MHz) spectrum of the compound **4b**

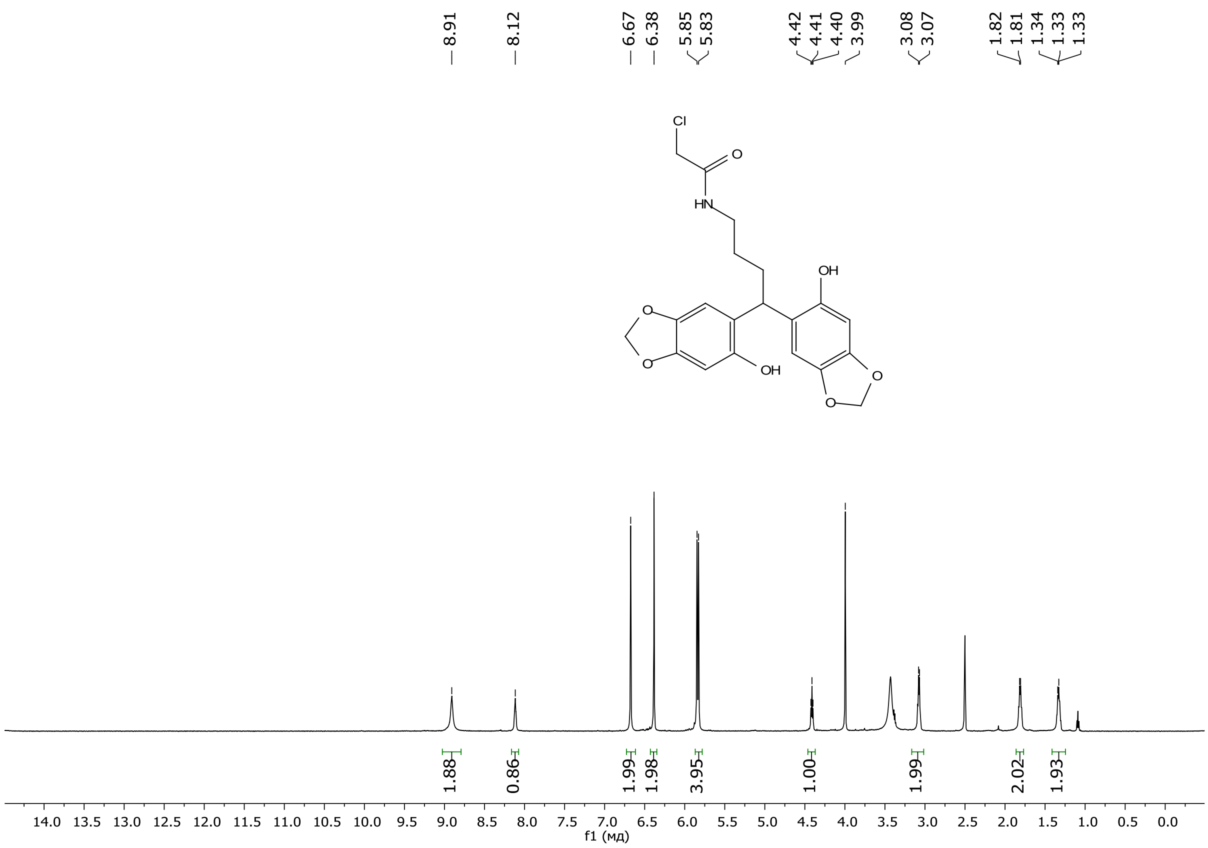

Figure S25. ^{1}H NMR (DMSO-d_6, 600 MHz) spectrum of the compound **4c**

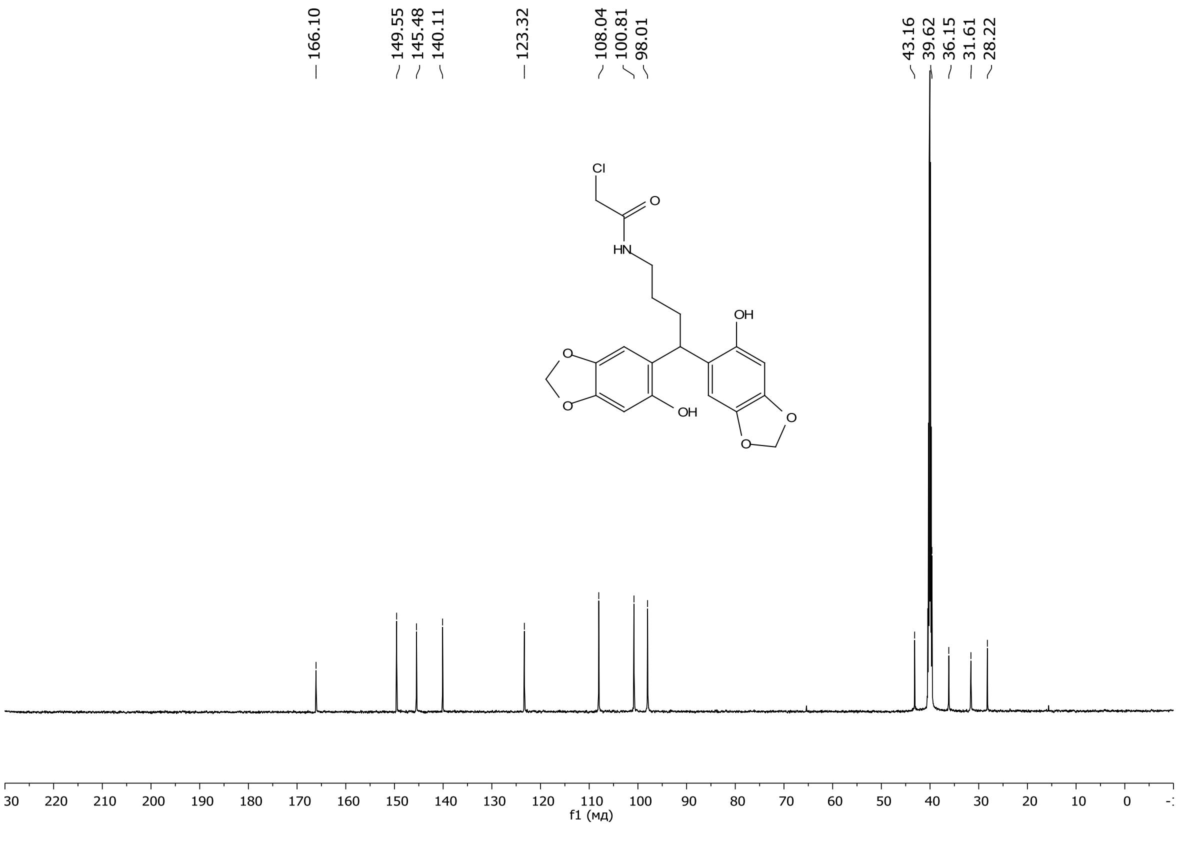

Figure S26. $^{13}C\{^{1}H\}$ NMR (DMSO-d_6, 150 MHz) spectrum of the compound **4c**

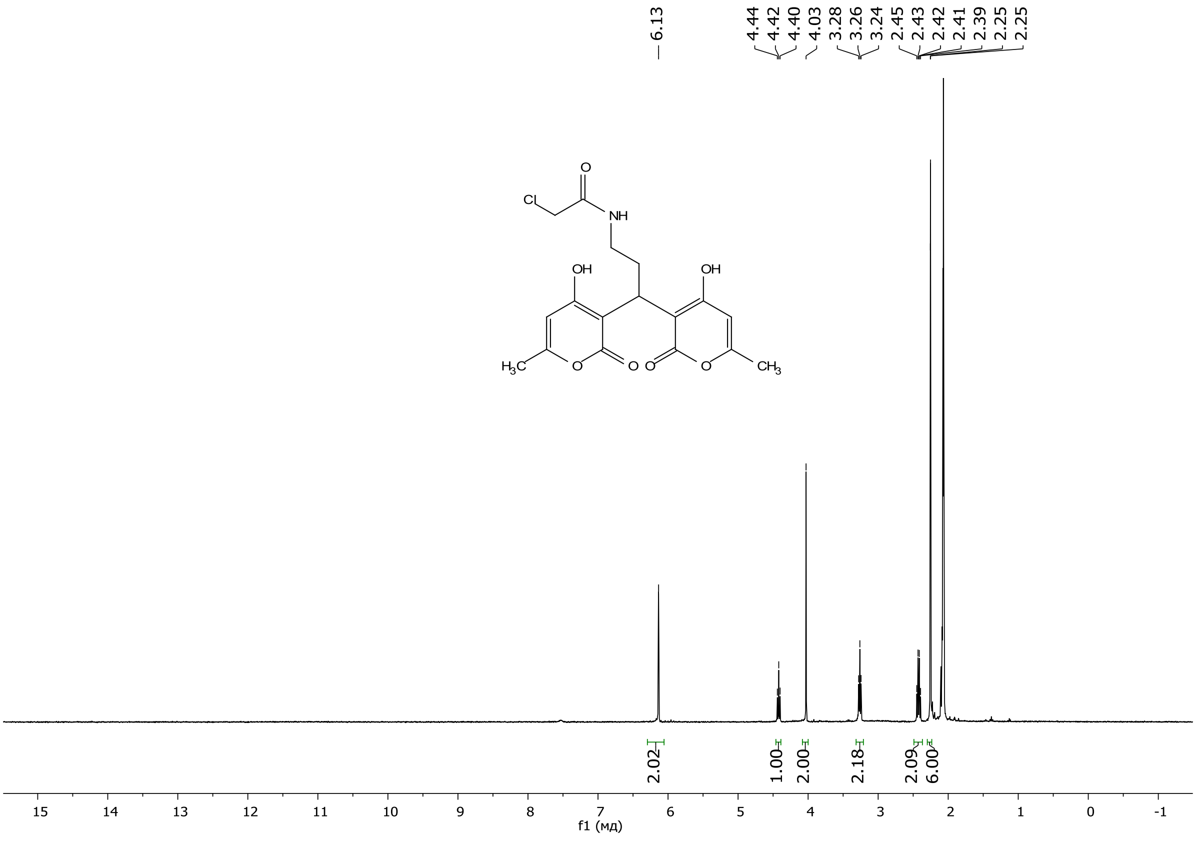

Figure S27. ^{1}H NMR (Acetone-d_6, 600 MHz) spectrum of the compound **5a**

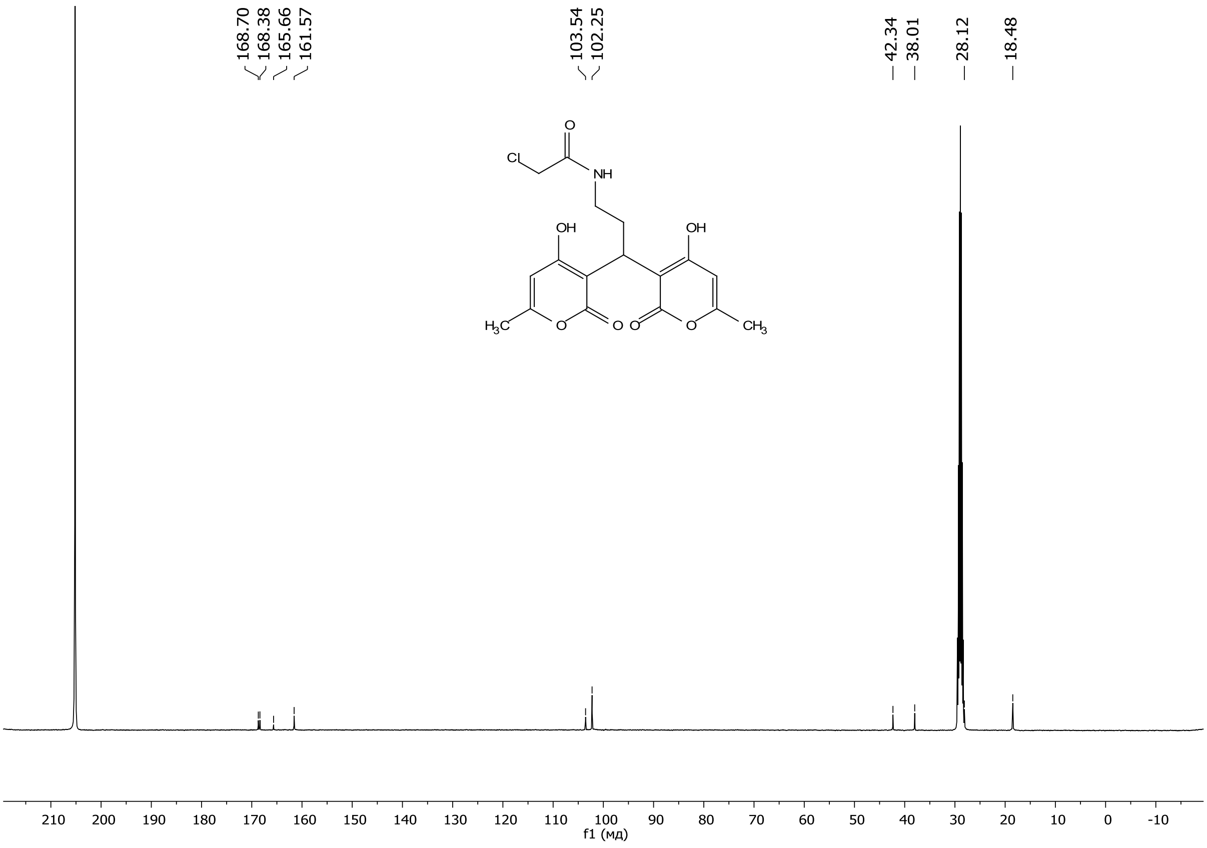

Figure S28. $^{13}C\{^{1}H\}$ NMR (Acetone-d_6, 150 MHz) spectrum of the compound **5a**

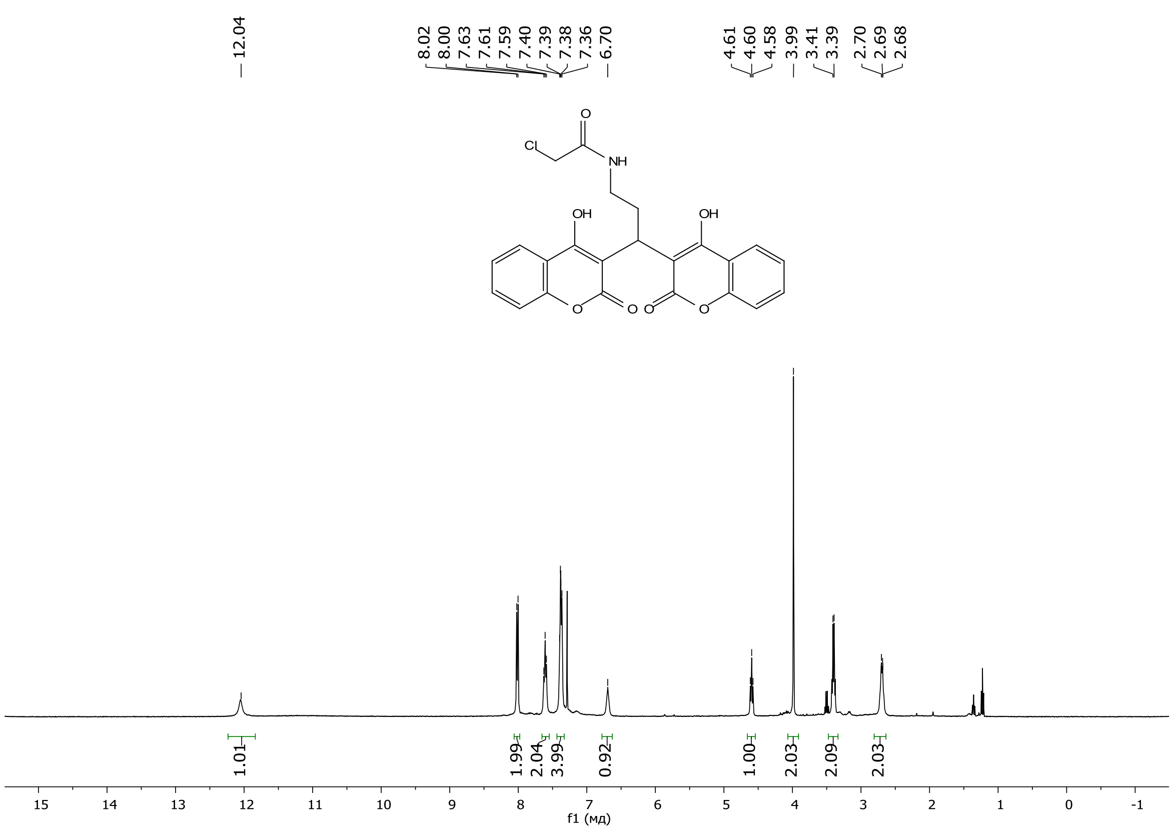

Figure S29. ^{1}H NMR ($CDCl_3$, 600 MHz) spectrum of the compound **5b**

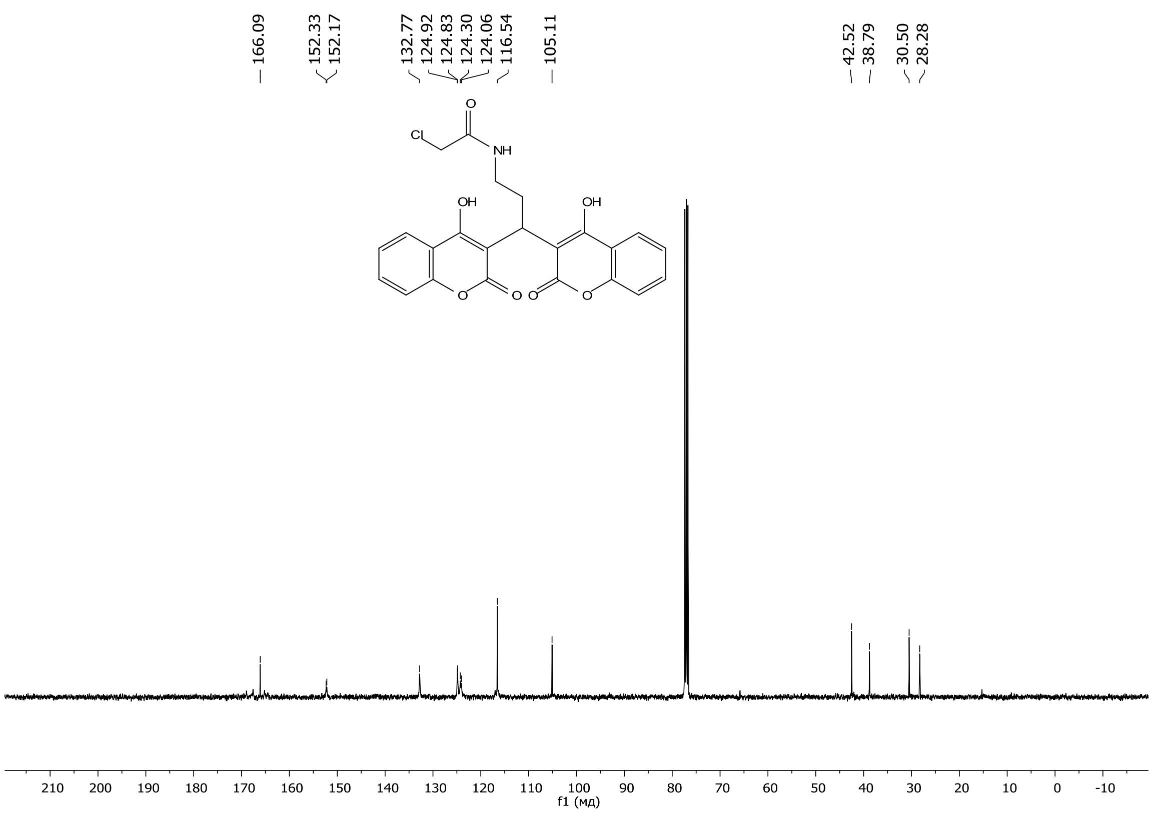

Figure S30. $^{13}C\{^{1}H\}$ NMR ($CDCl_3$, 150 MHz) spectrum of the compound **5b**

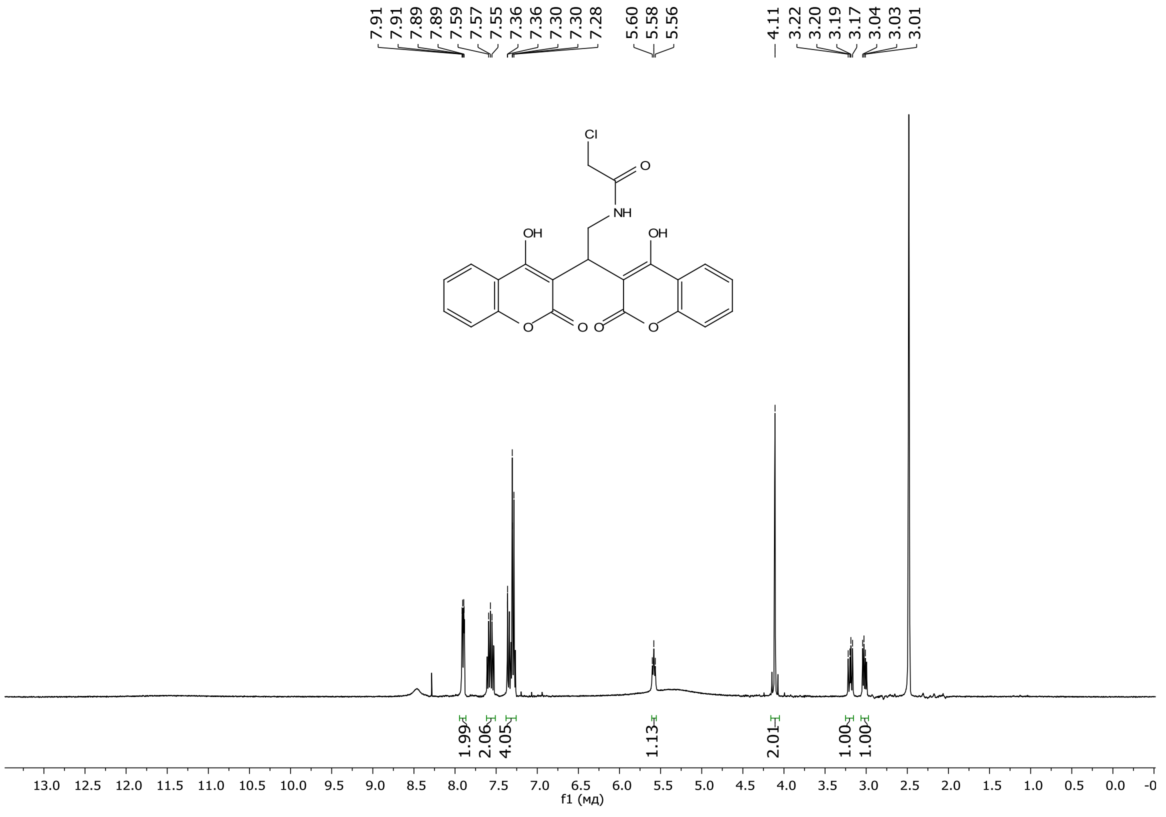

Figure S31. ^{1}H NMR (DMSO-d_6, 600 MHz) spectrum of the compound **5c**

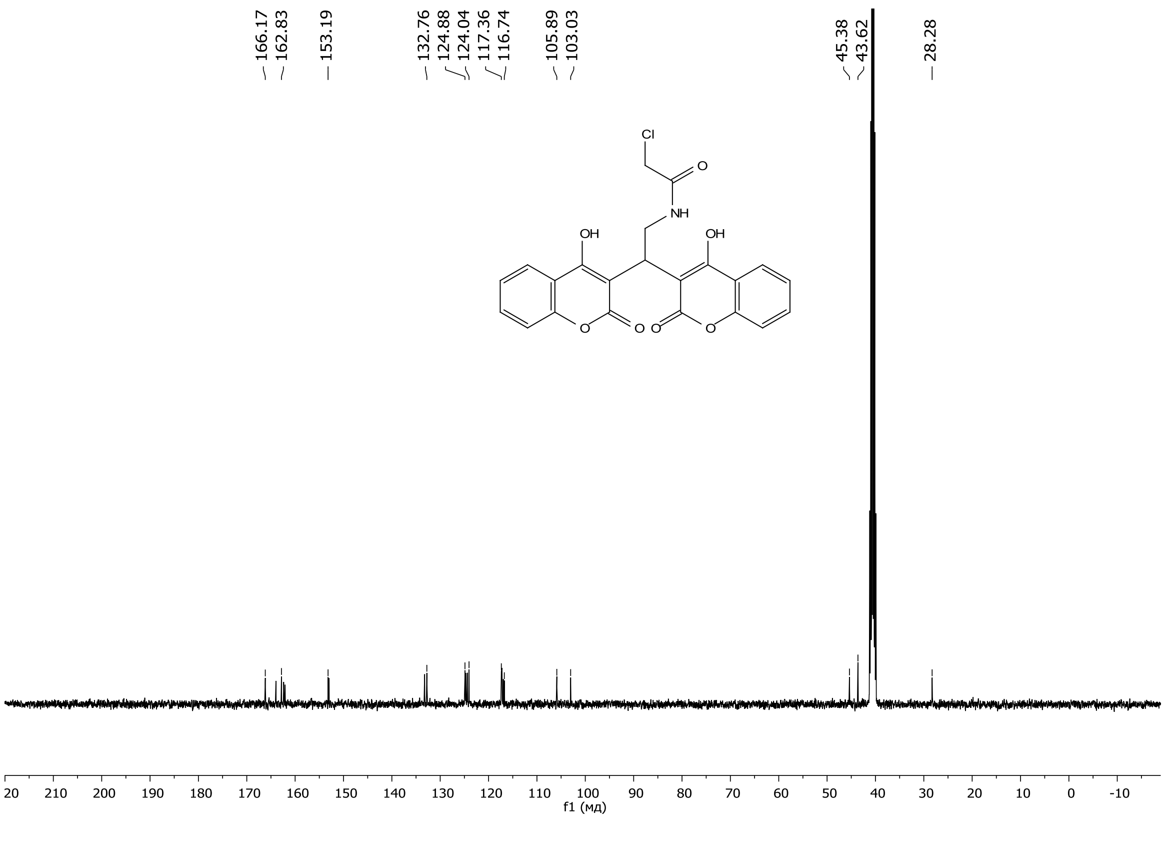

Figure S32. $^{13}C\{^{1}H\}$ NMR (DMSO-d_6, 150 MHz) spectrum of the compound **5c**

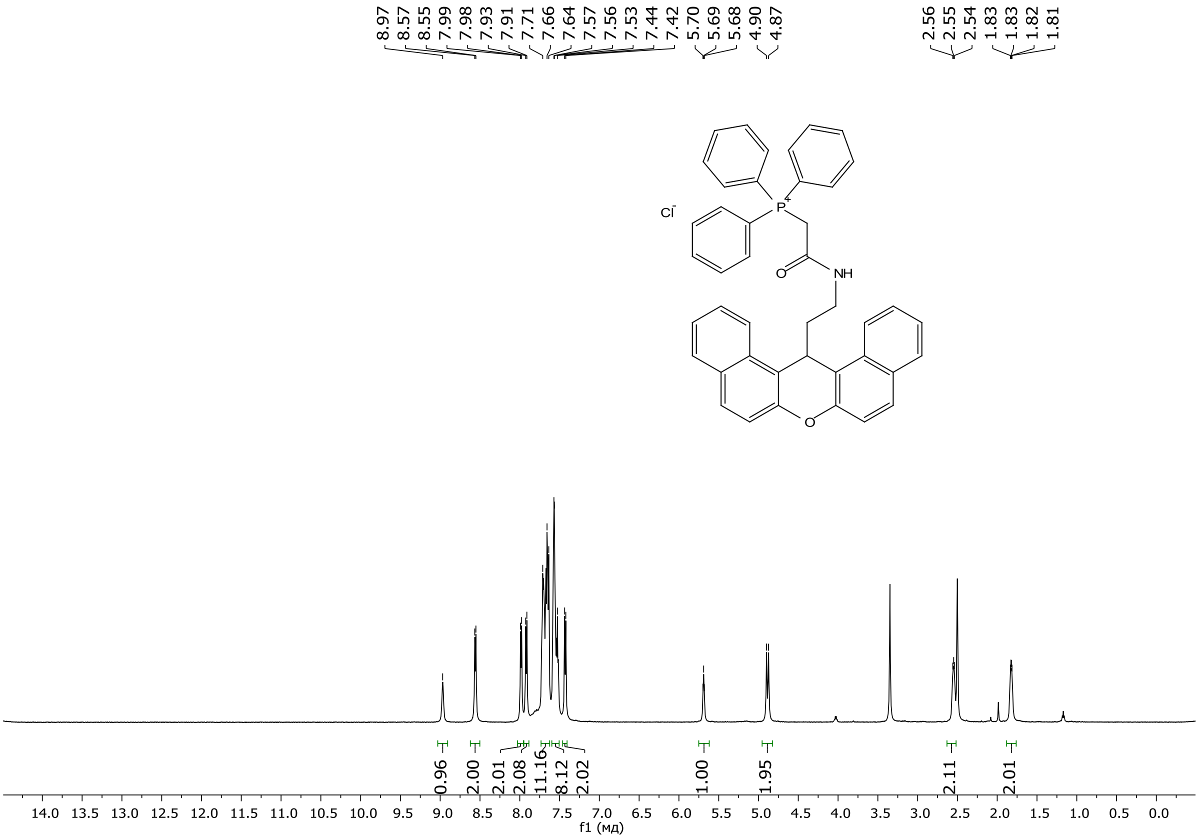

Figure S33. ^{1}H NMR (DMSO-d_6, 600 MHz) spectrum of the compound **6a**

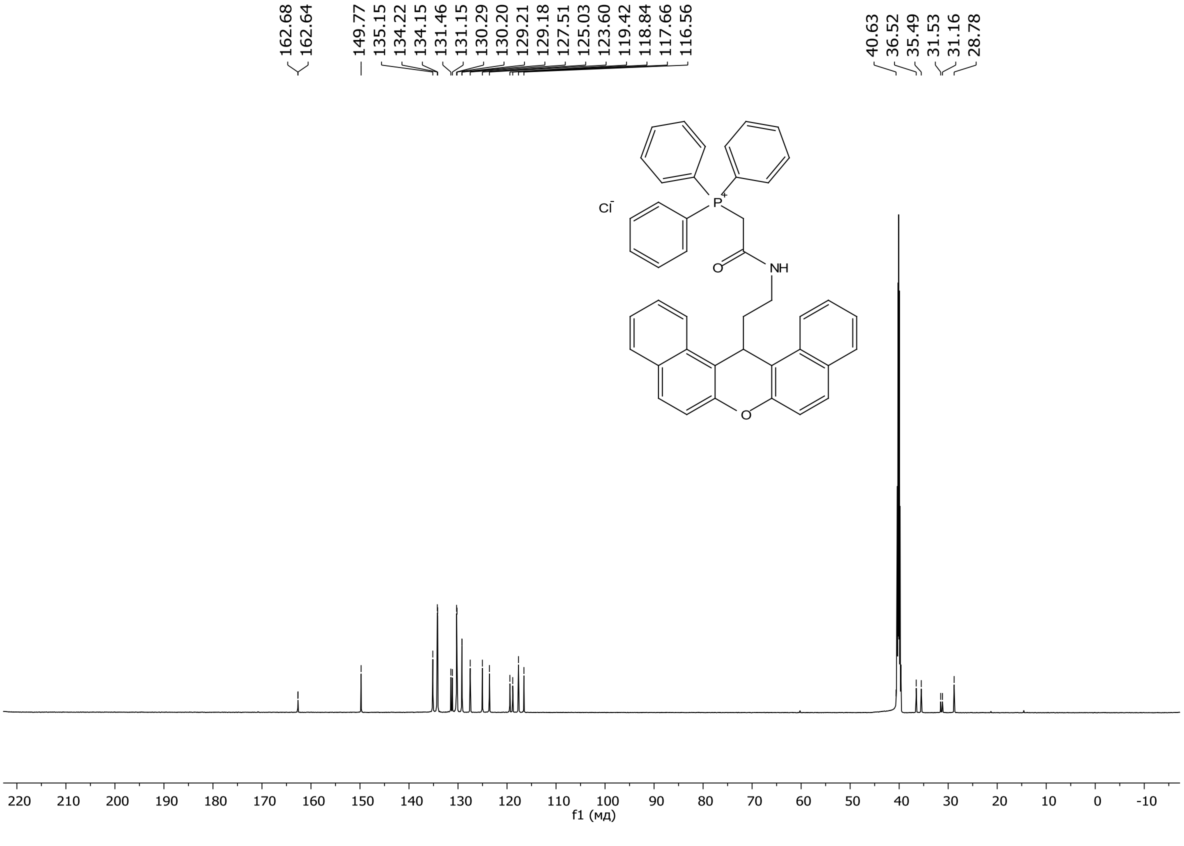

Figure S34. ^{13}C{^{1}H} NMR (DMSO-d_6, 150 MHz) spectrum of the compound **6a**

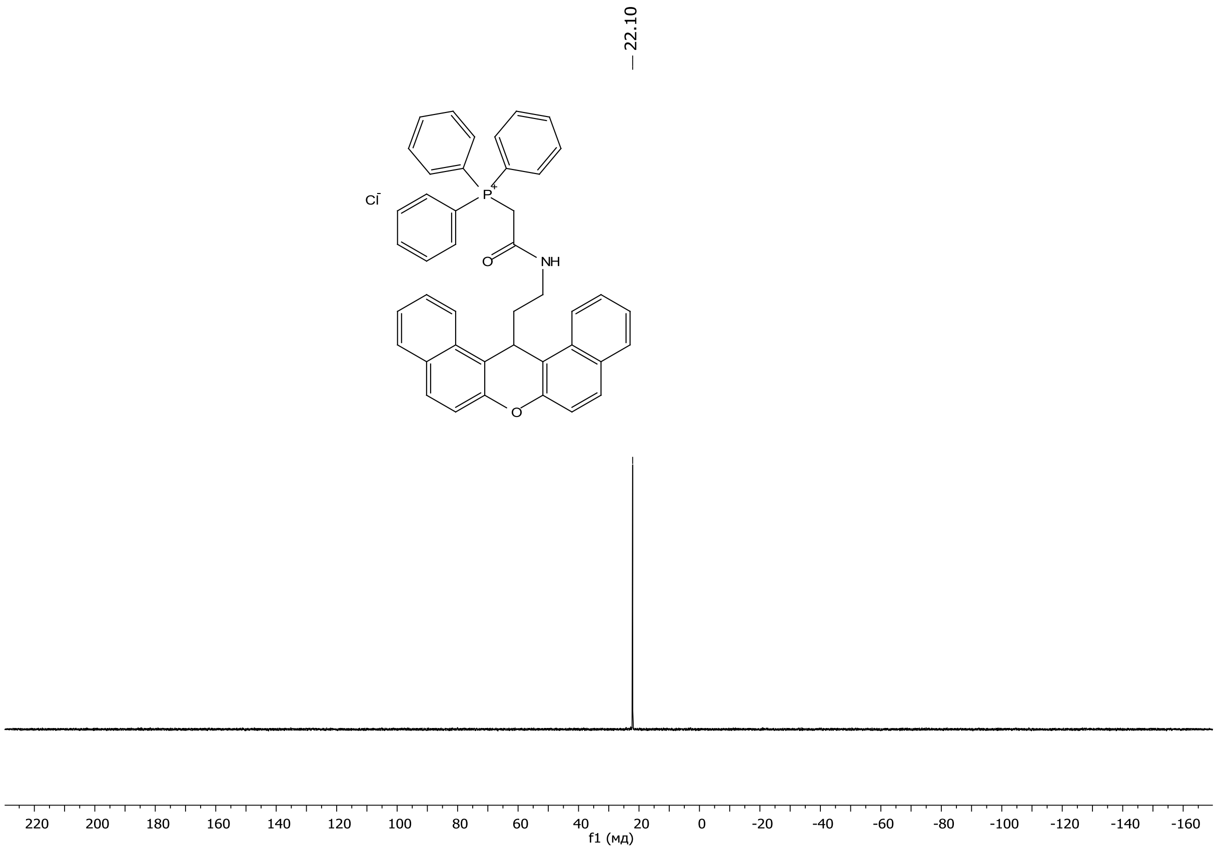

Figure S35. $^{31}P\{^{1}H\}$ NMR (DMSO-d_6, 161 MHz) spectrum of the compound **6a**

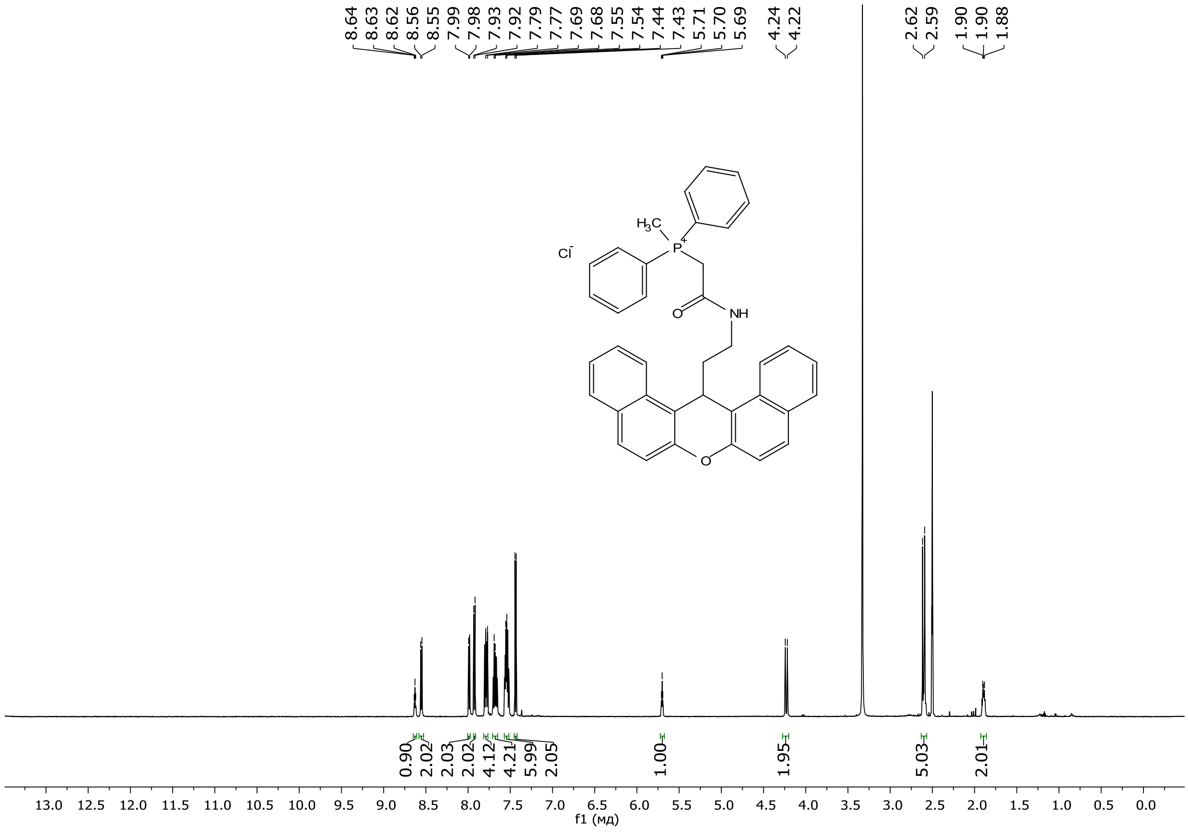

Figure S36. ^{1}H NMR (DMSO-d_6, 600 MHz) spectrum of the compound **6b**

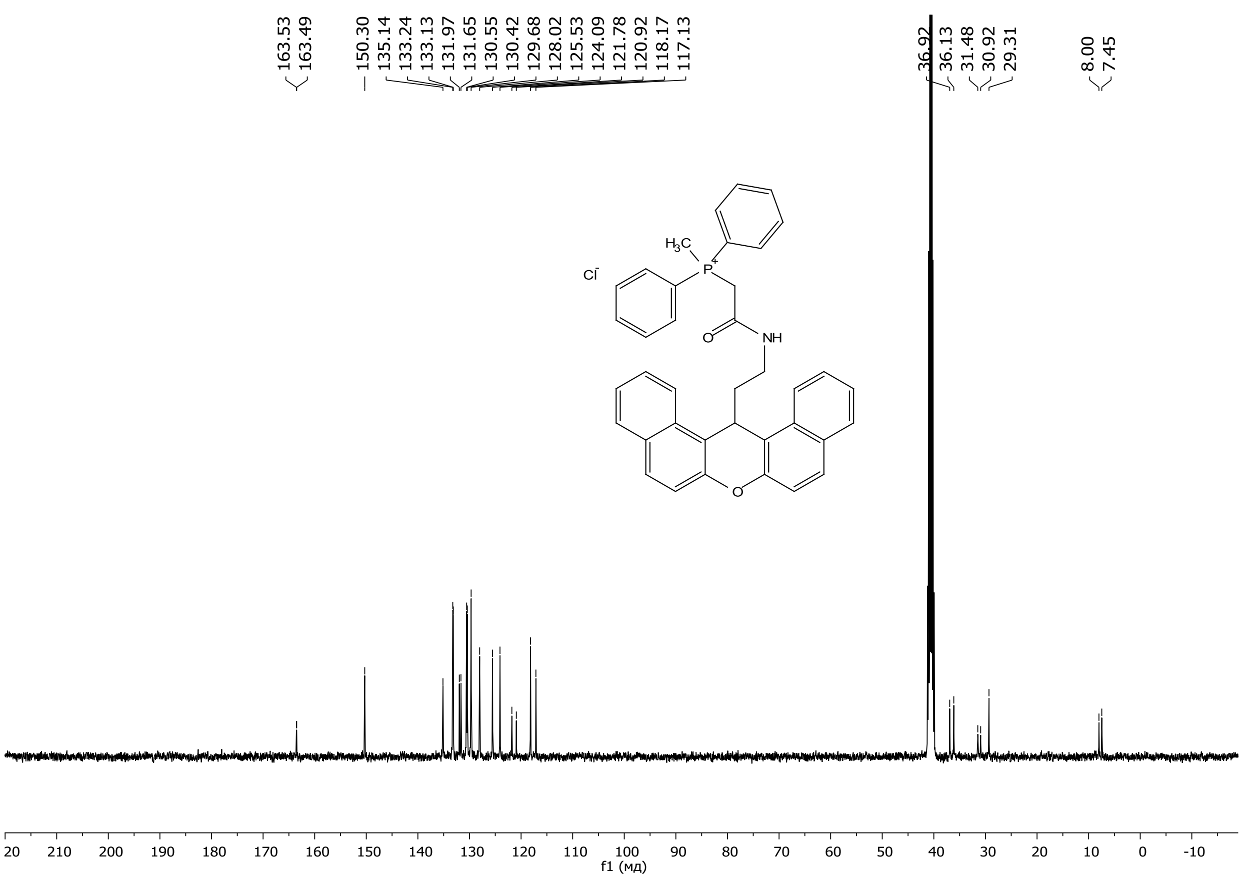

Figure S37. $^{13}C\{^{1}H\}$ NMR (DMSO-d_6, 150 MHz) spectrum of the compound **6b**

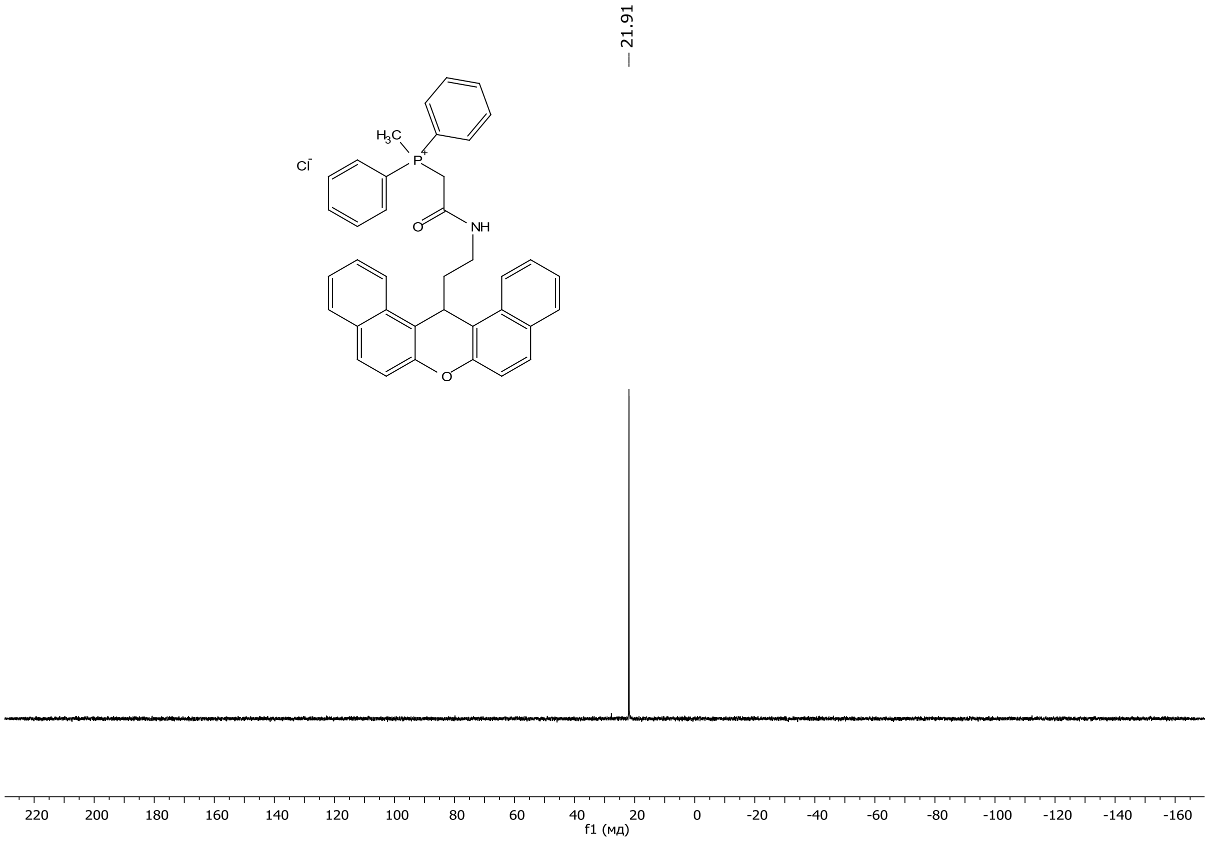

Figure S38. $^{31}P\{^{1}H\}$ NMR (DMSO-d_6, 161 MHz) spectrum of the compound **6b**

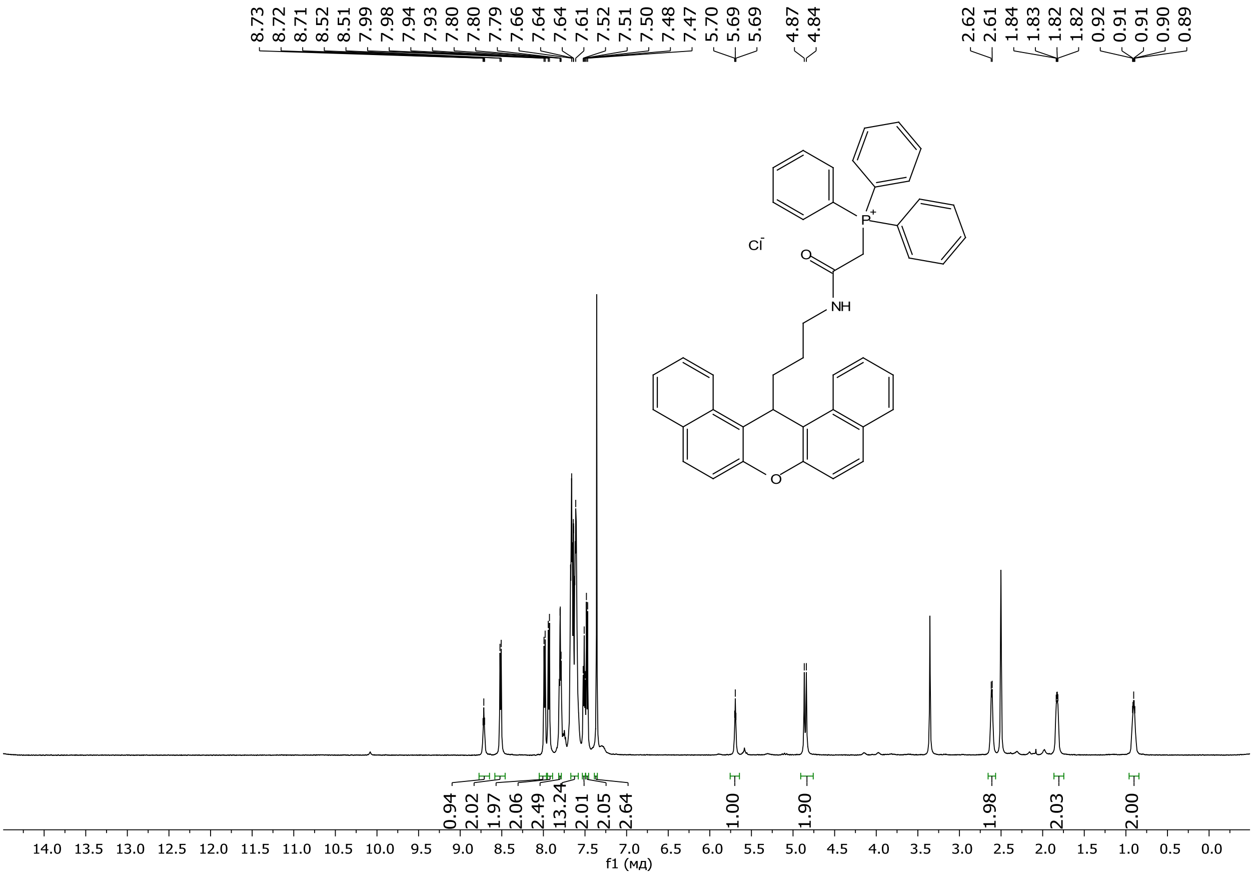

Figure S39. ^{1}H NMR (DMSO-d_6, 600 MHz) spectrum of the compound **6c**

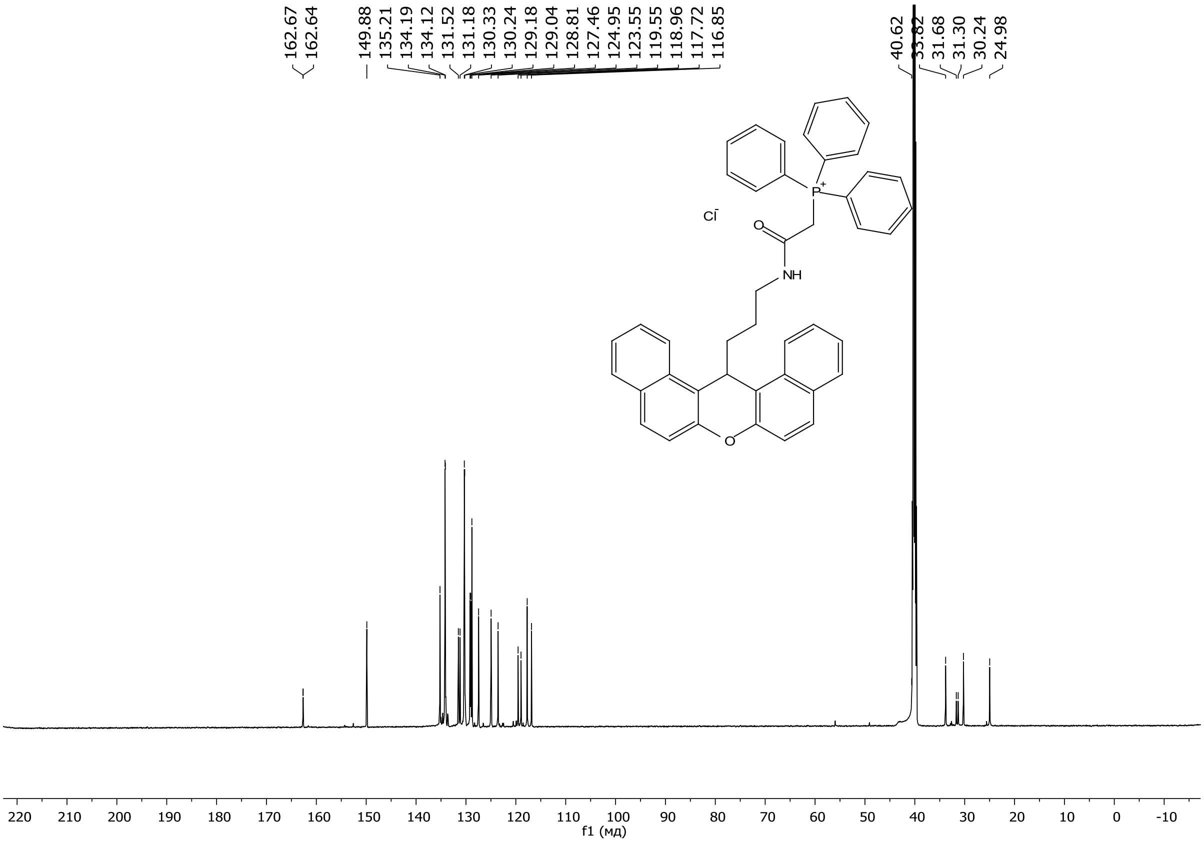

Figure S40. $^{13}C\{^{1}H\}$ NMR (DMSO-d_6, 150 MHz) spectrum of the compound **6c**

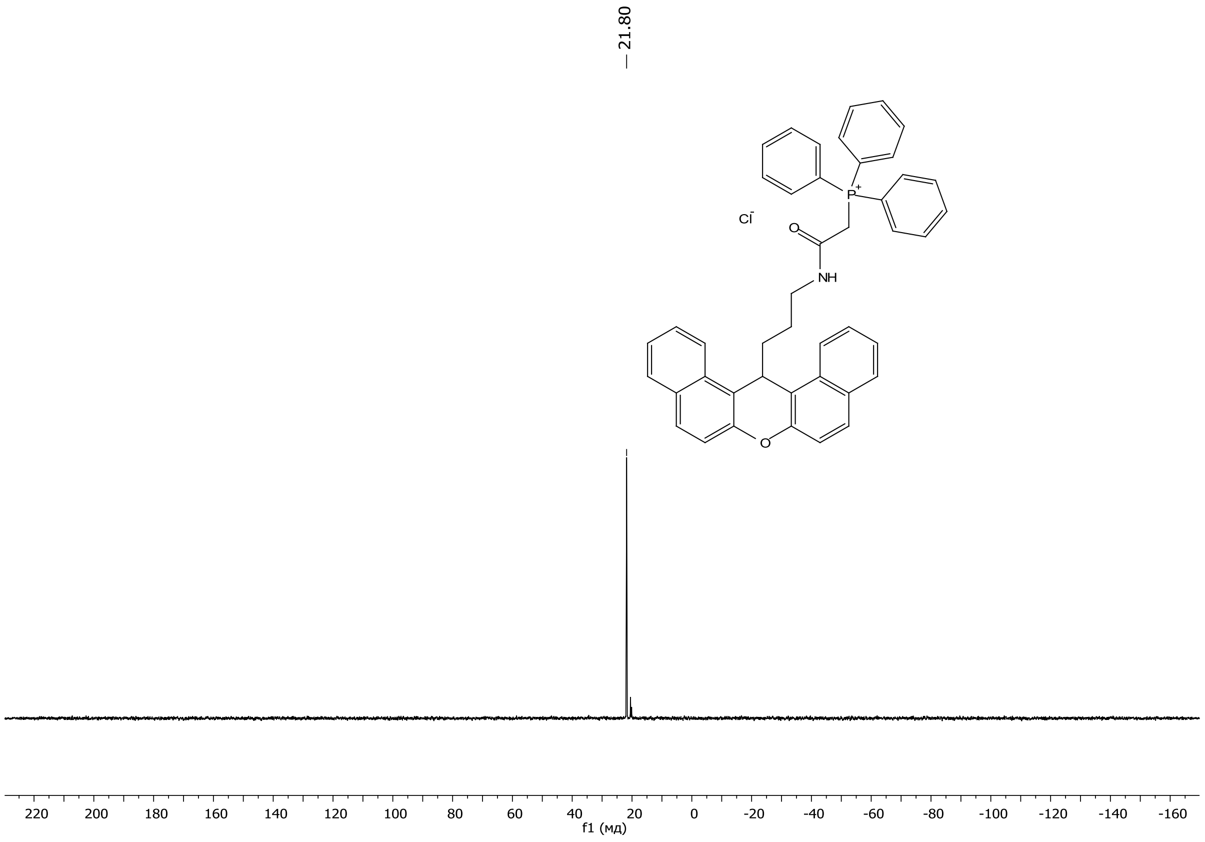

Figure S41. $^{31}P\{^1H\}$ NMR (DMSO-d_6, 161 MHz) spectrum of the compound **6c**

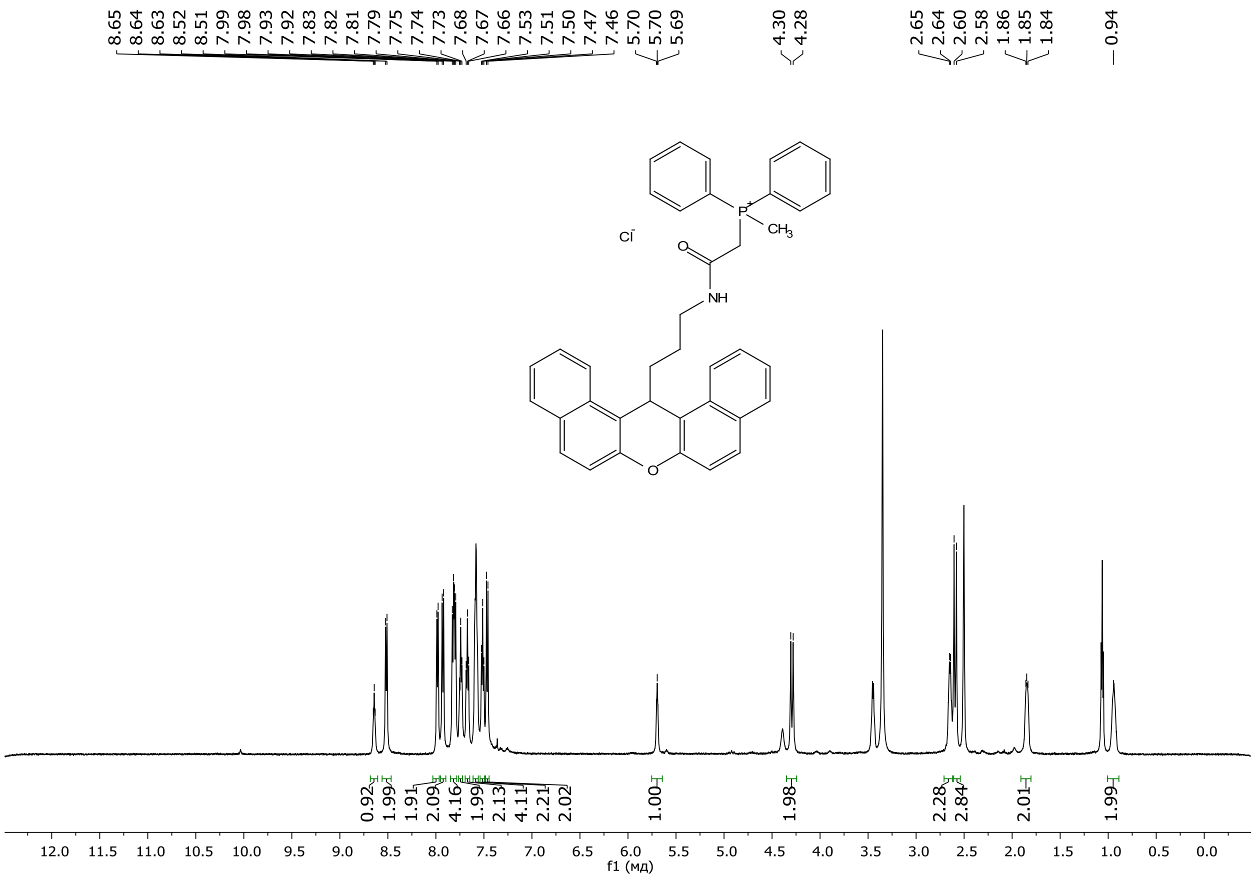

Figure S42. ^1H NMR (DMSO-d_6, 600 MHz) spectrum of the compound **6d**

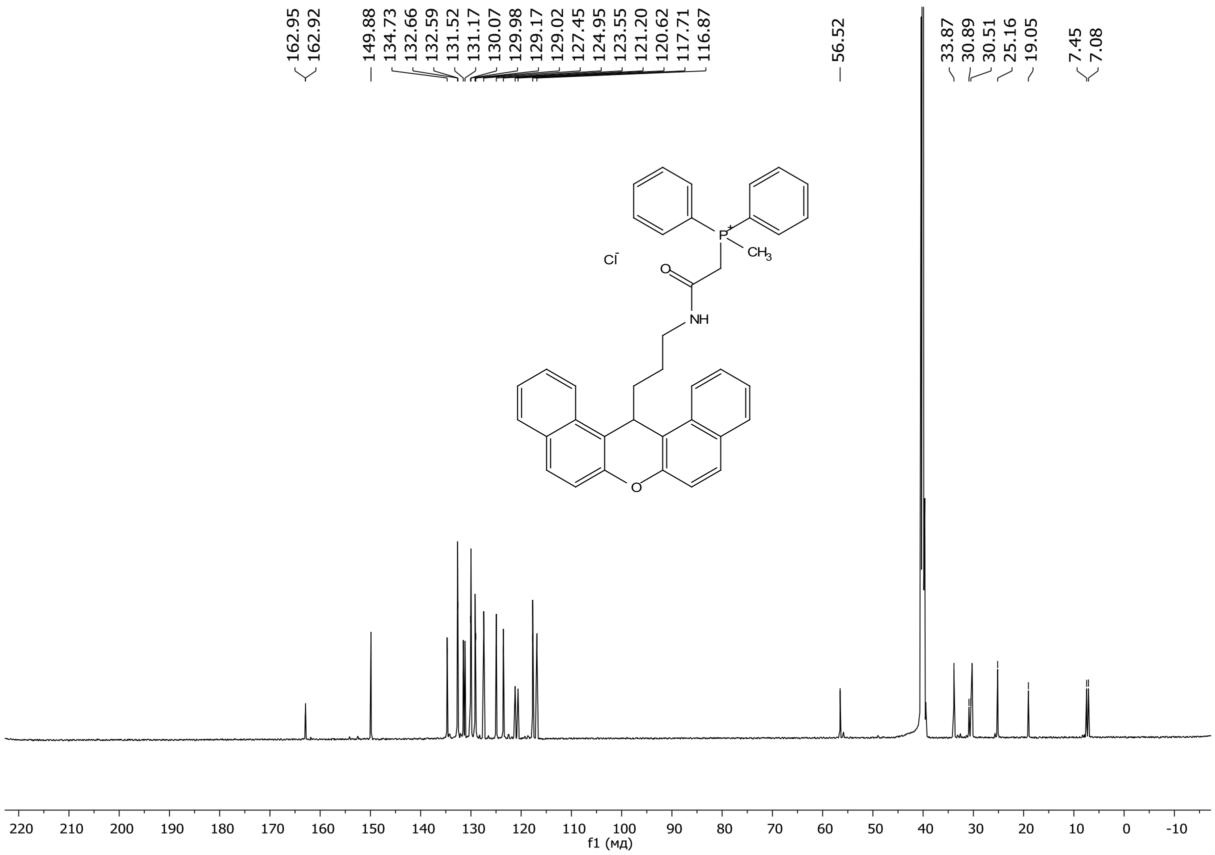

Figure S43. $^{13}C\{^{1}H\}$ NMR (DMSO-d_6, 150 MHz) spectrum of the compound **6d**

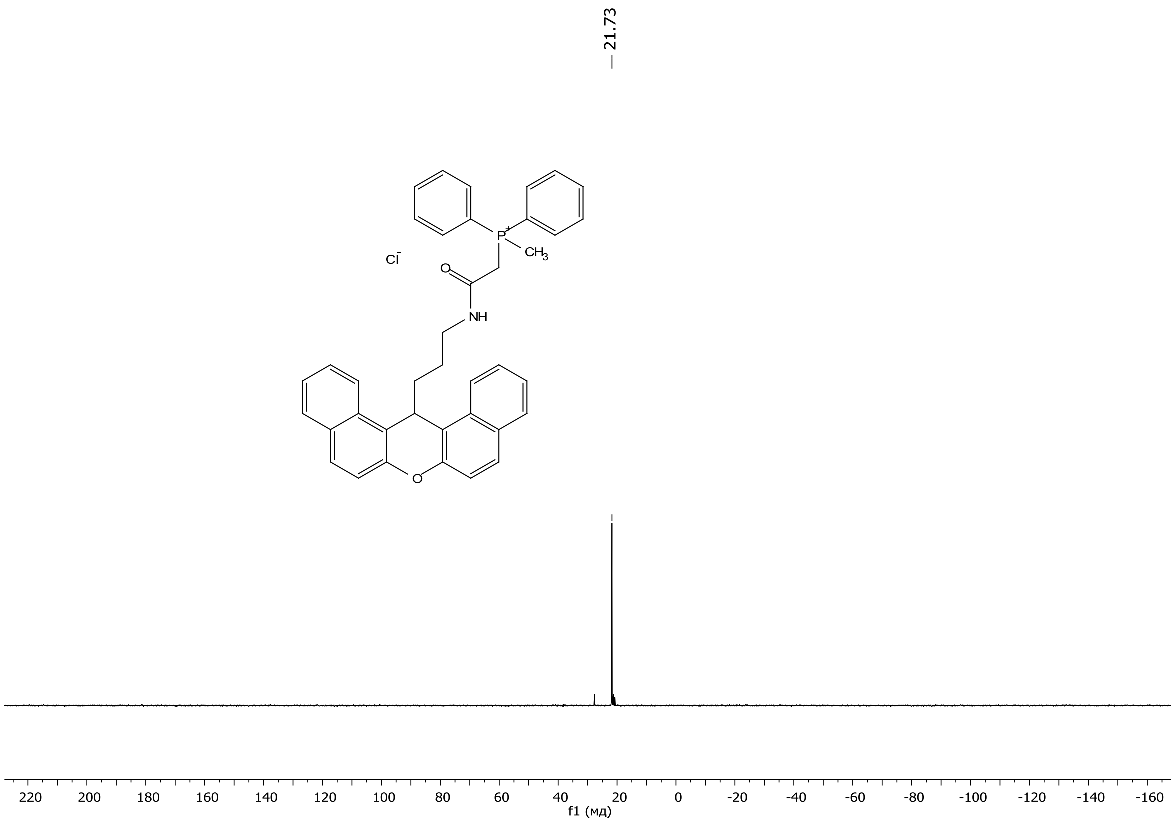

Figure S44. $^{31}P\{^{1}H\}$ NMR (DMSO-d_6, 161 MHz) spectrum of the compound **6d**

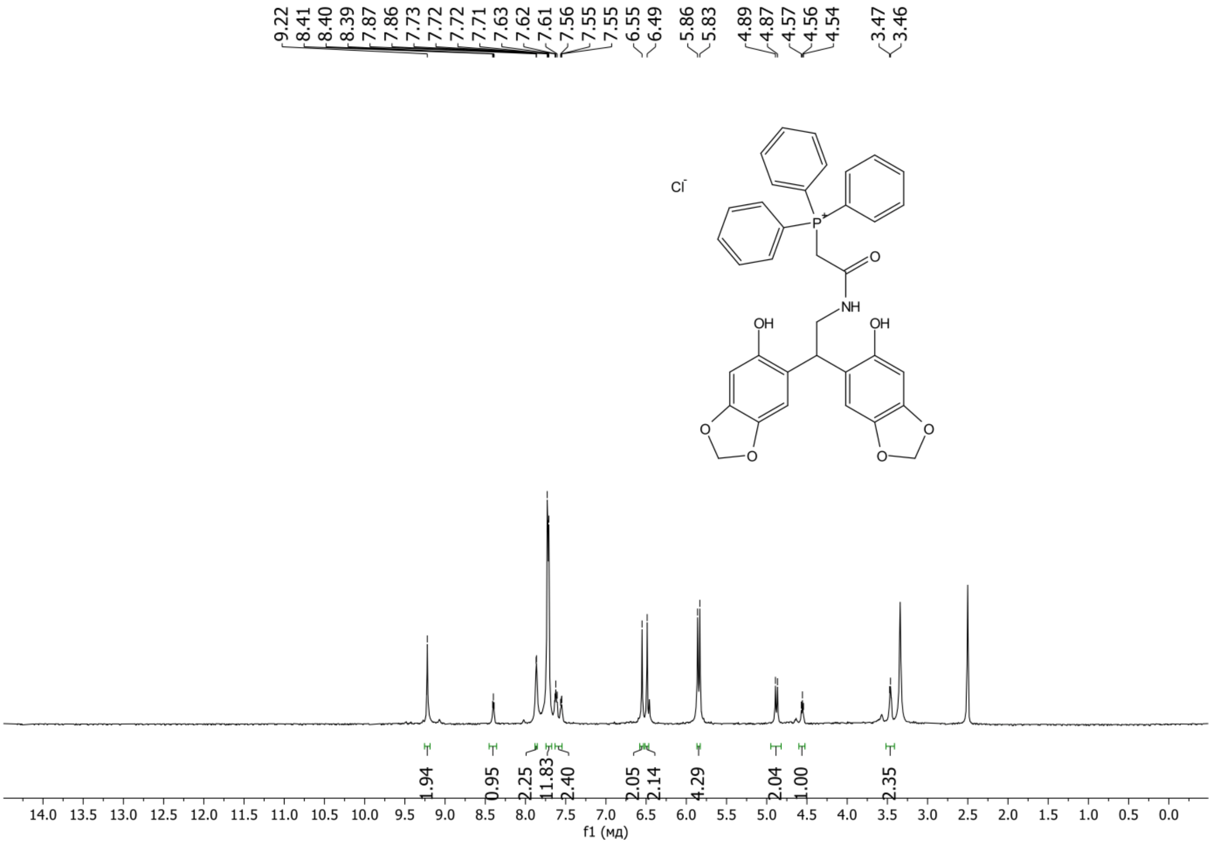

Figure S45. ^{1}H NMR (DMSO-d_6, 600 MHz) spectrum of the compound **7a**

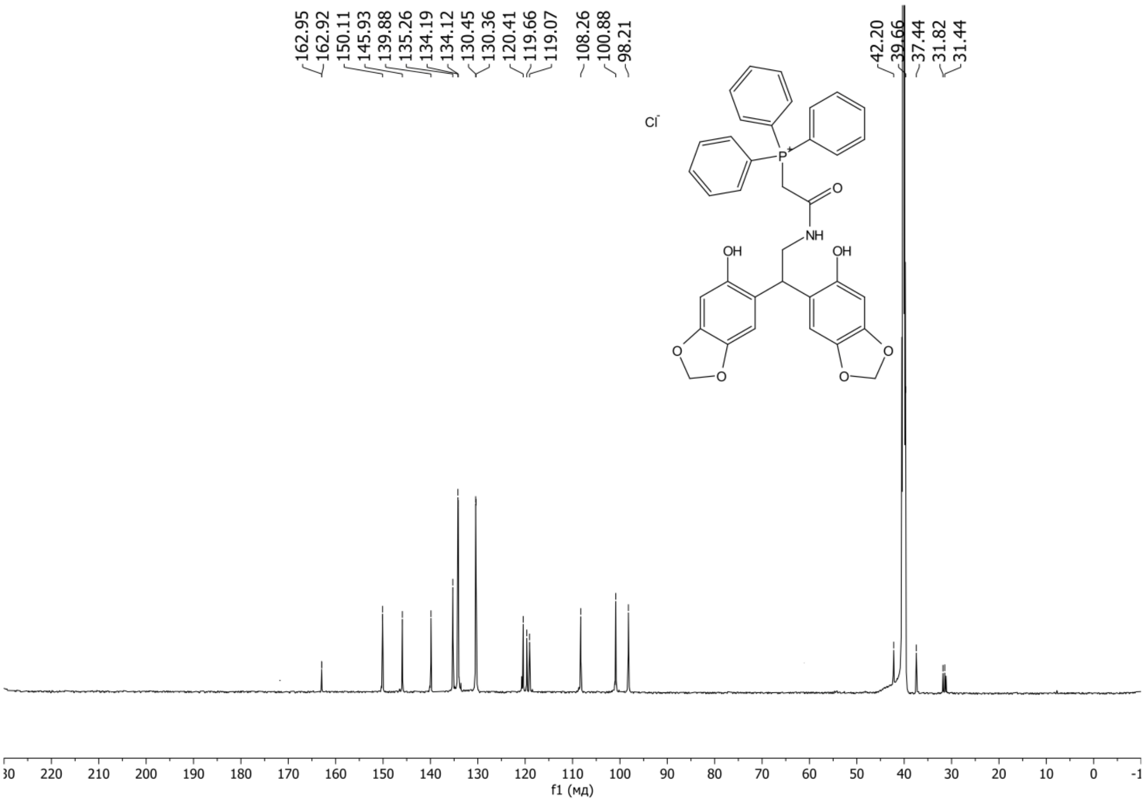

Figure S46. $^{13}C\{^{1}H\}$ NMR (DMSO-d_6, 150 MHz) spectrum of the compound **7a**

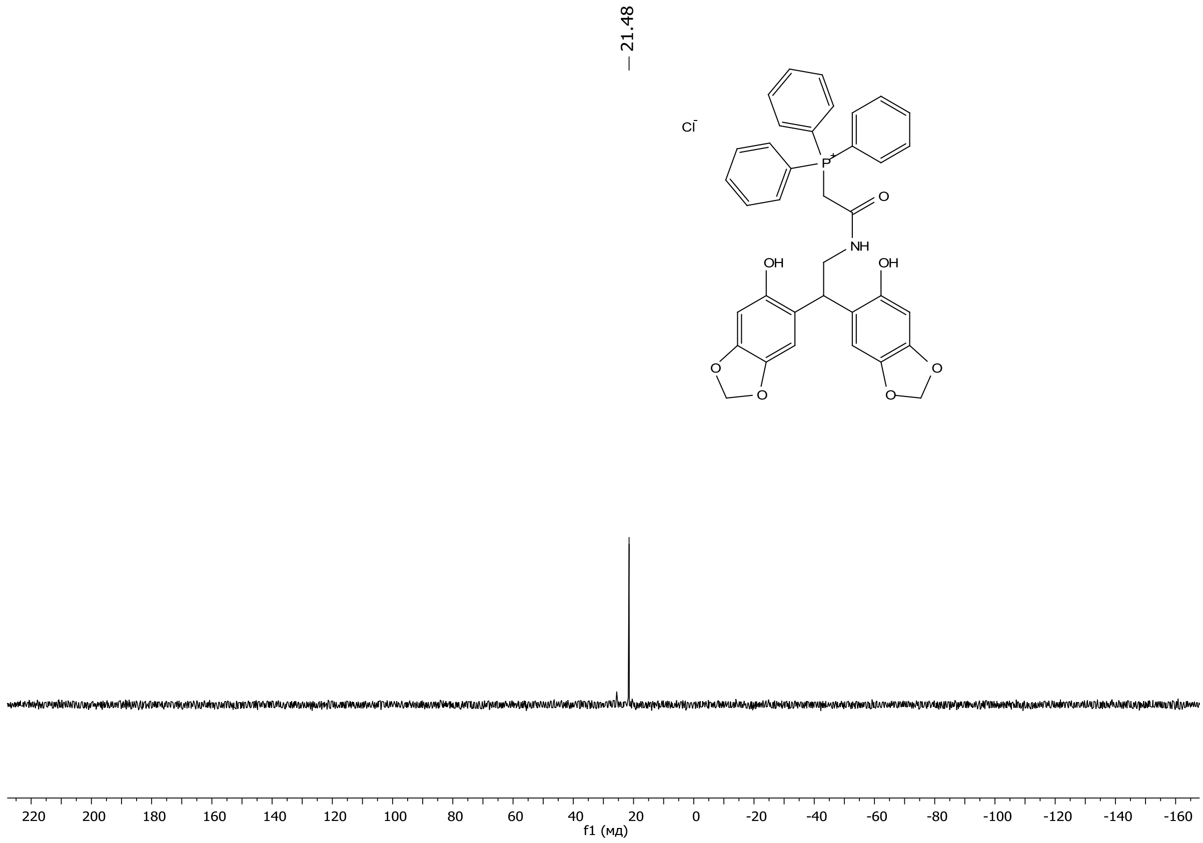

Figure S47. $^{31}P\{^{1}H\}$ NMR (DMSO-d_6, 161 MHz) spectrum of the compound **7a**

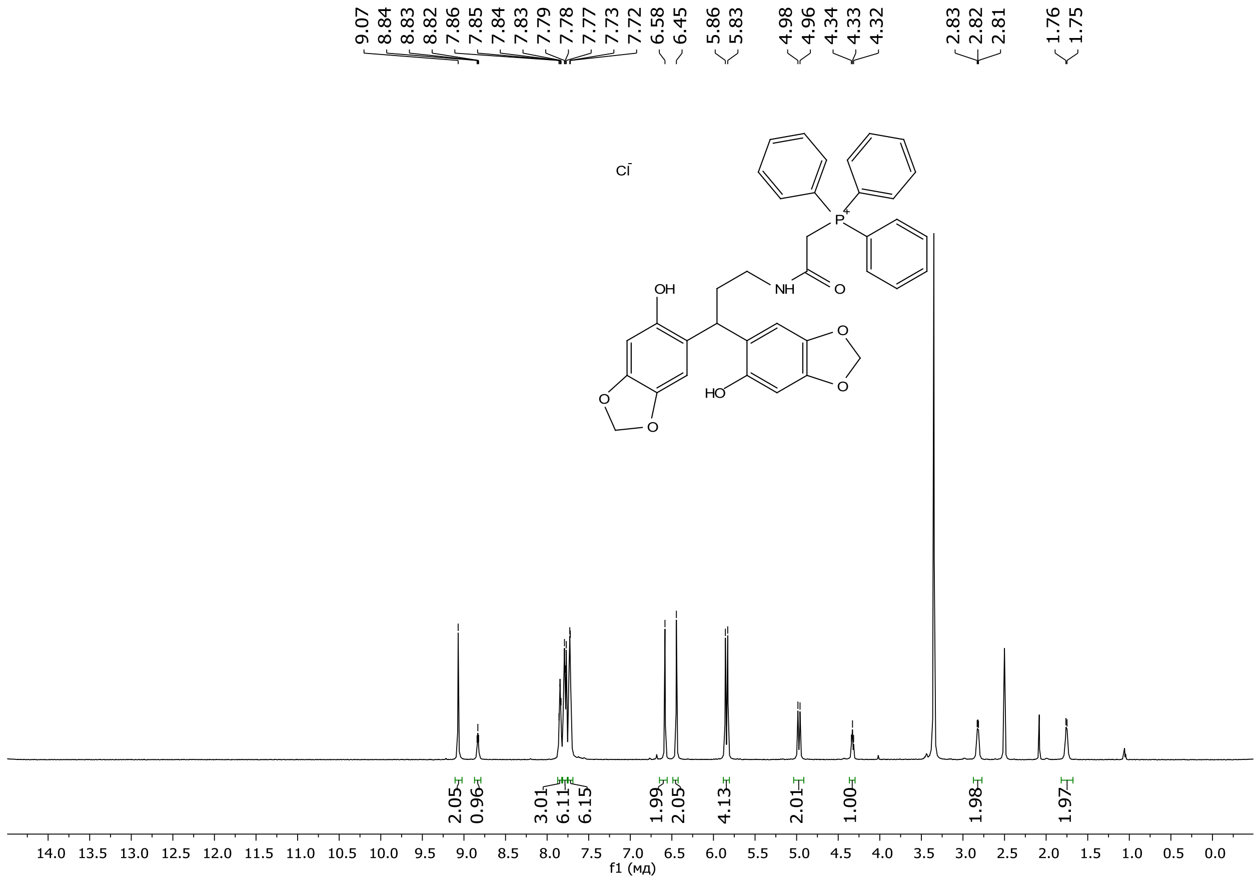

Figure S48. ^{1}H NMR (DMSO-d_6, 600 MHz) spectrum of the compound **7b**

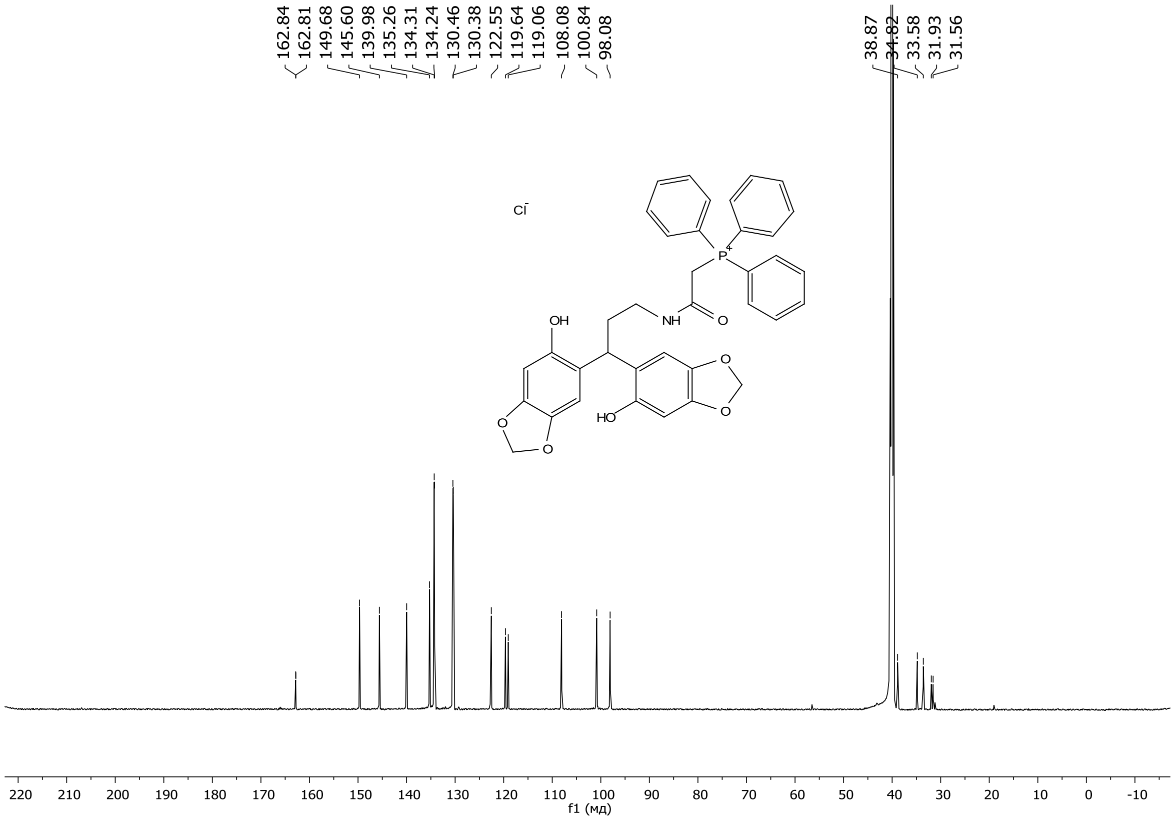

Figure S49. $^{13}C\{^1H\}$ NMR (DMSO-d_6, 150 MHz) spectrum of the compound **7b**

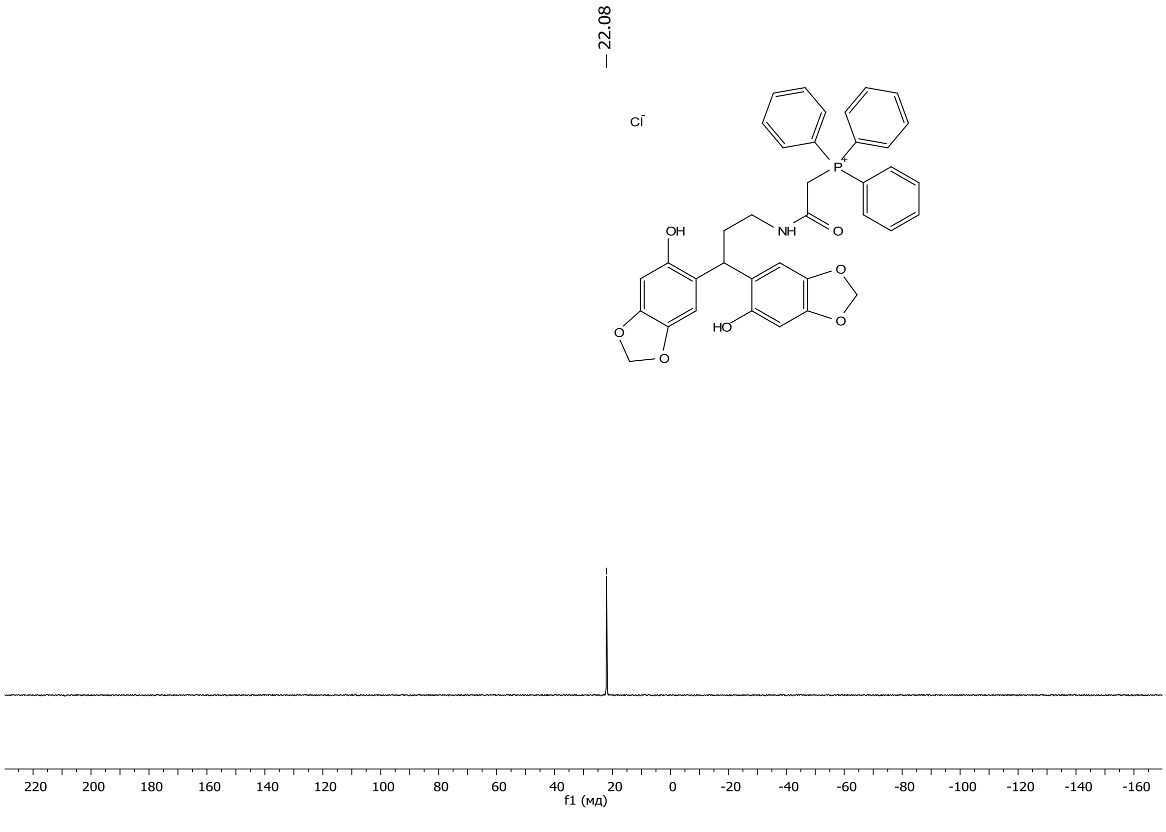

Figure S50. $^{31}P\{^1H\}$ NMR (DMSO-d_6, 161 MHz) spectrum of the compound **7b**

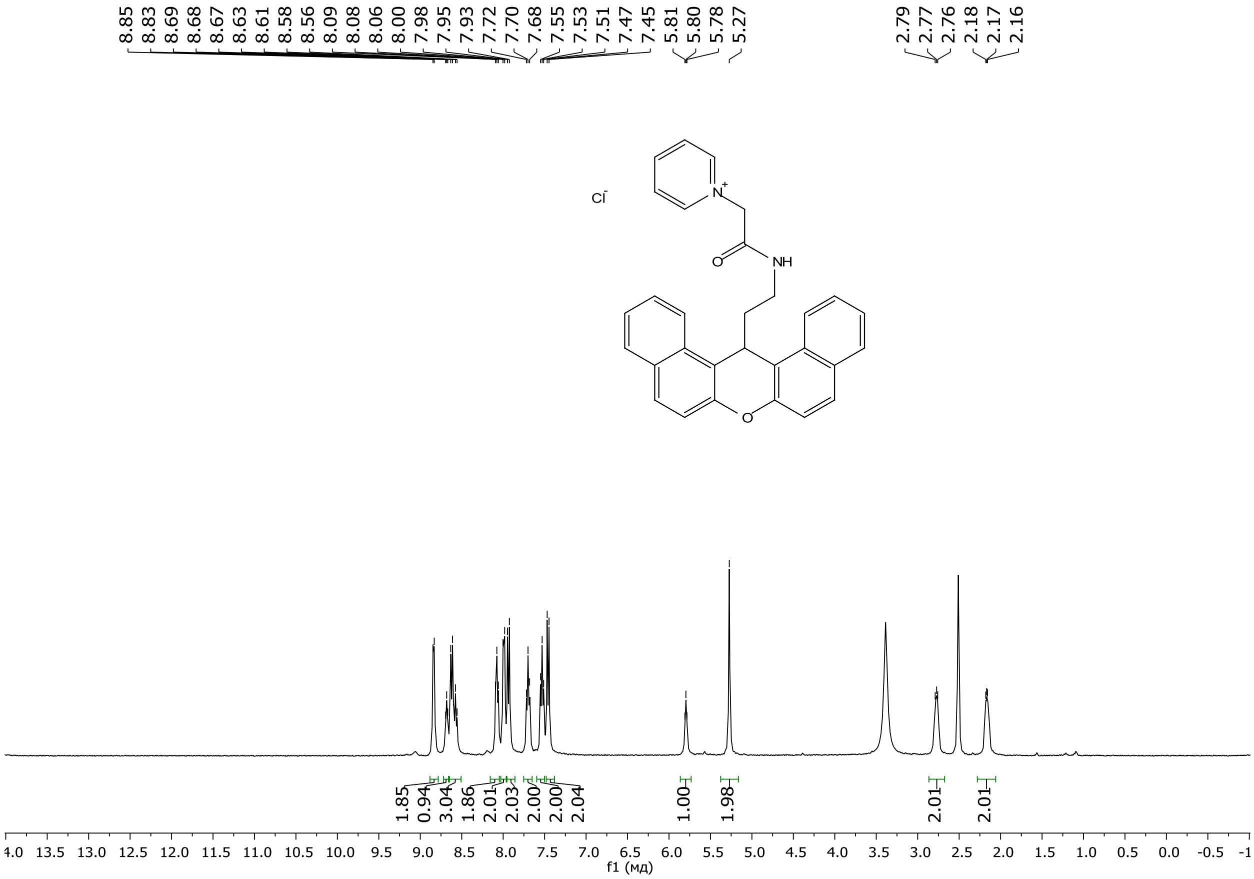

Figure S51. ^{1}H NMR (DMSO-d_6, 600 MHz) spectrum of the compound **8**

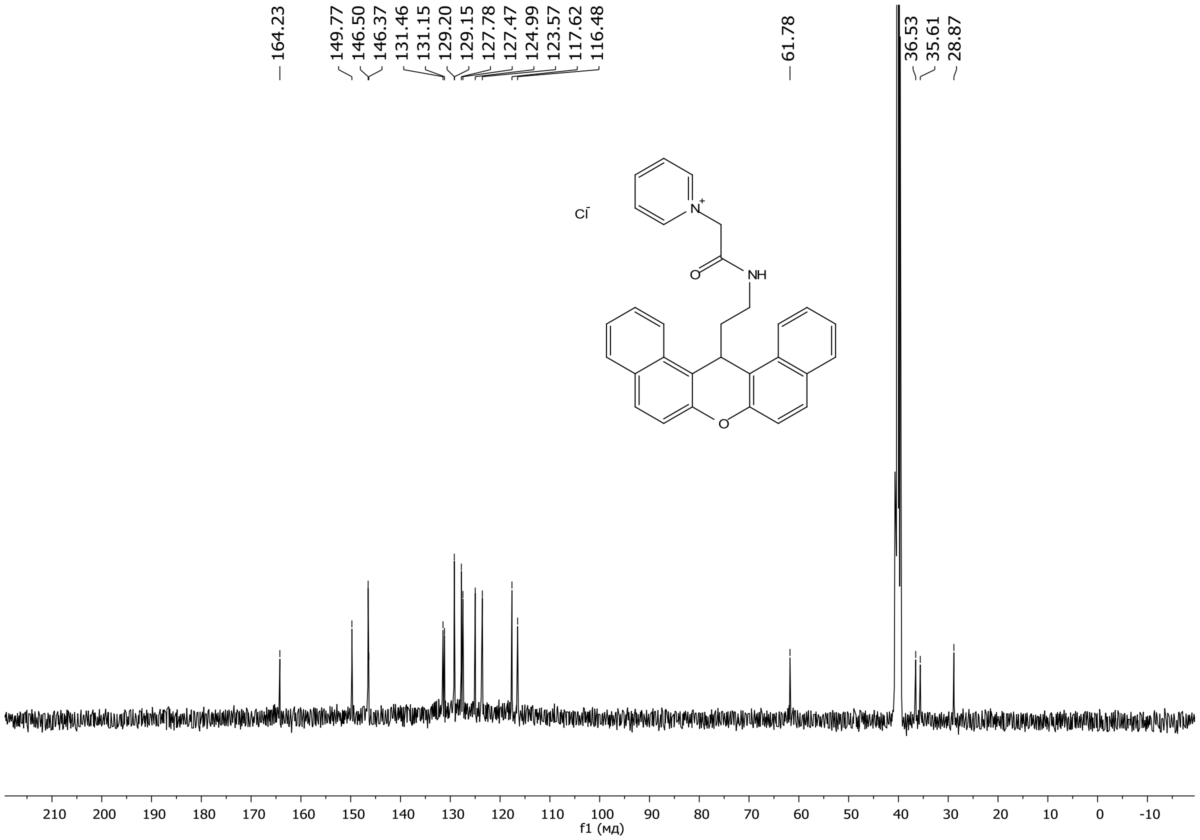

Figure S52. ^{13}C{^{1}H} NMR (DMSO-d_6, 150 MHz) spectrum of the compound **8**

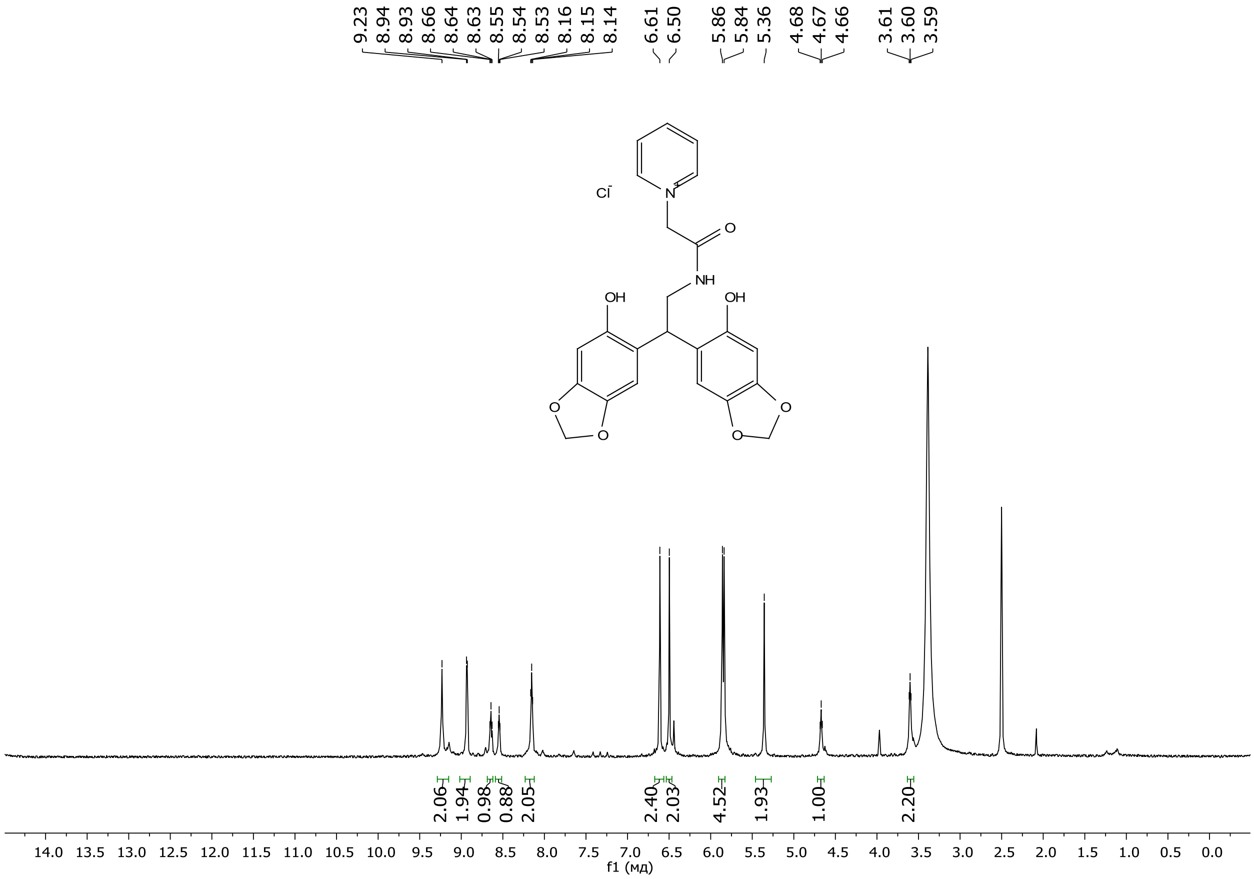

Figure S53. ^{1}H NMR (DMSO-d_6, 600 MHz) spectrum of the compound **9a**

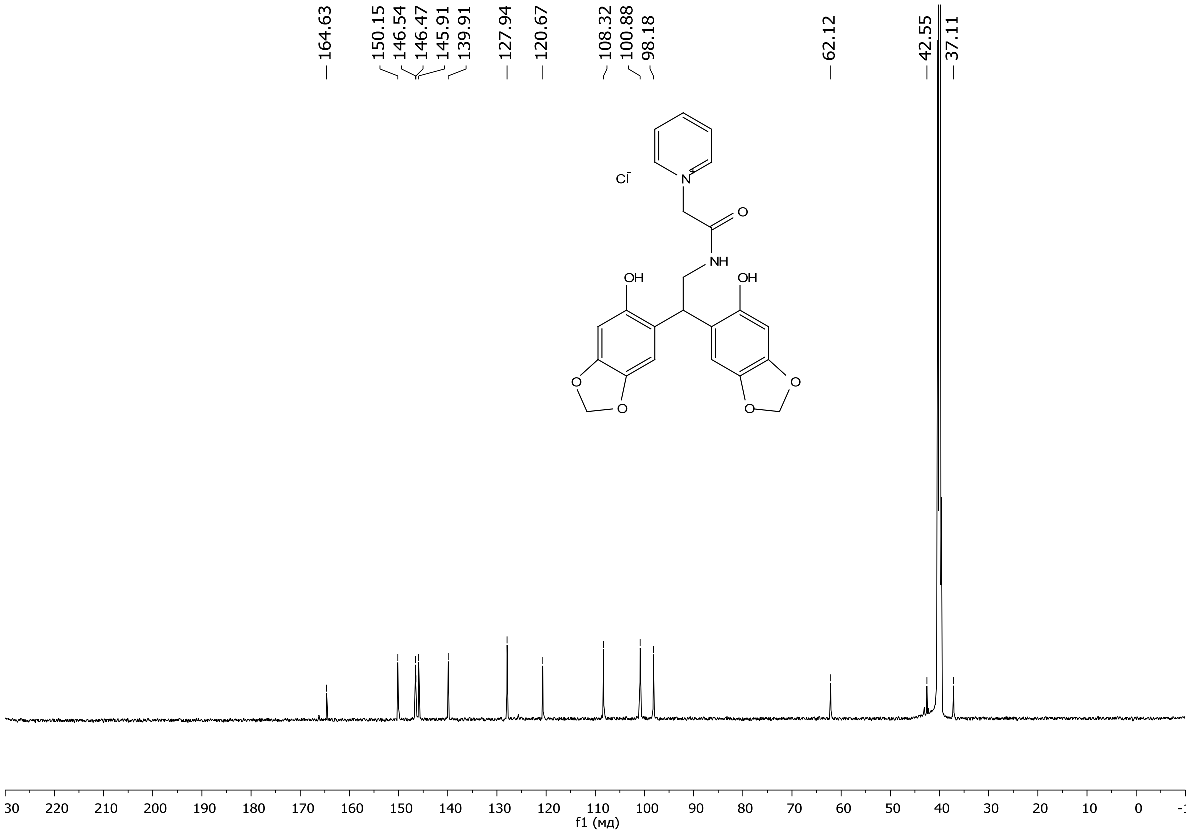

Figure S54. $^{13}C\{^{1}H\}$ NMR (DMSO-d_6, 150 MHz) spectrum of the compound **9a**

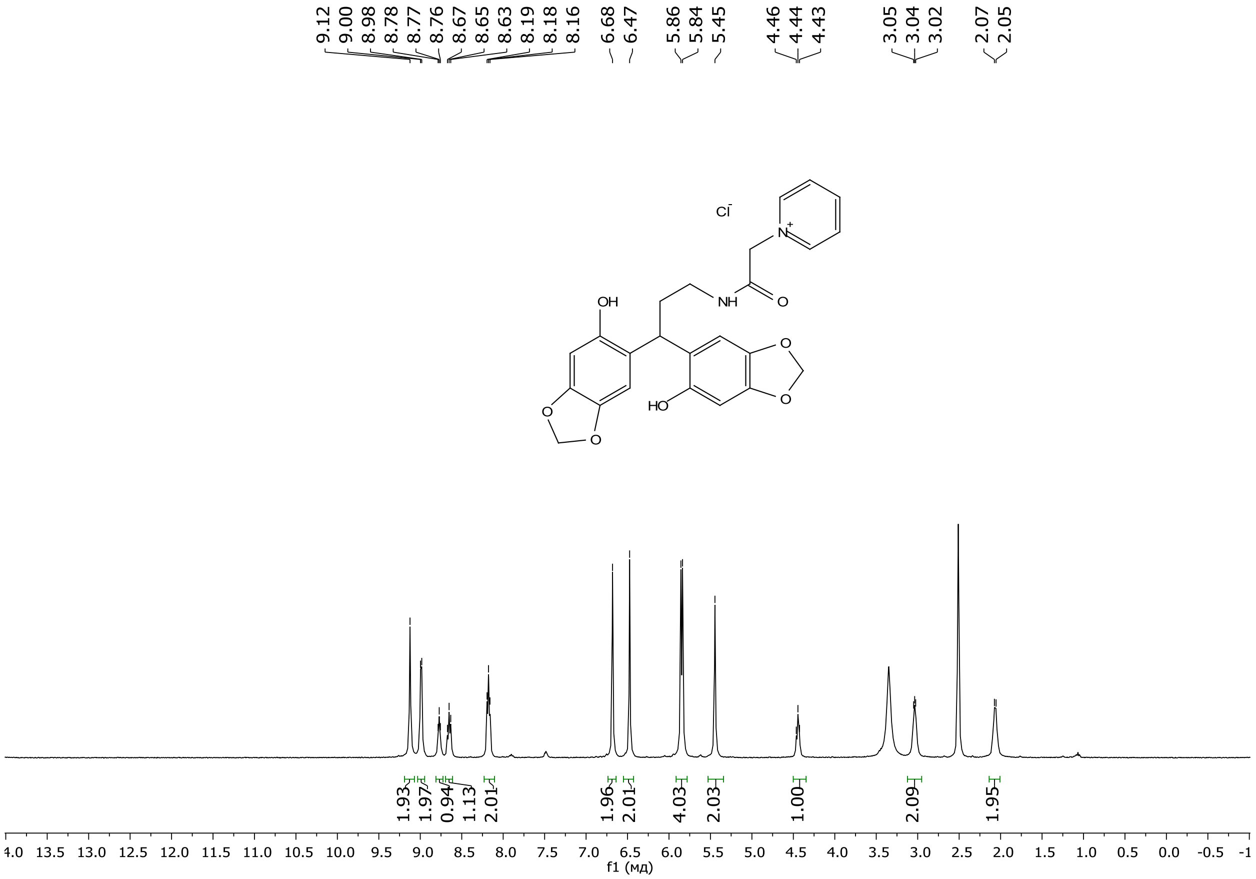

Figure S55. ^{1}H NMR (DMSO-d_6, 600 MHz) spectrum of the compound **9b**

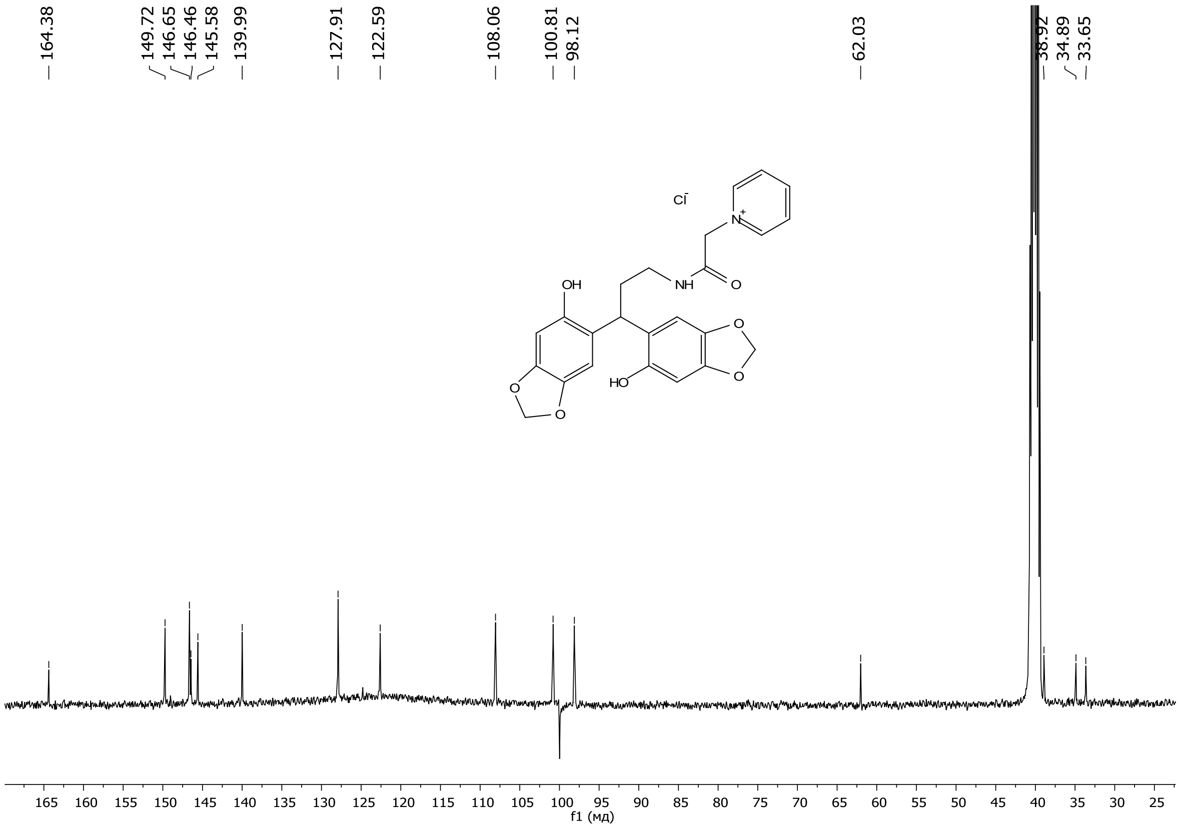

Figure S56. ^{13}C{^{1}H} NMR (DMSO-d_6, 150 MHz) spectrum of the compound **9b**

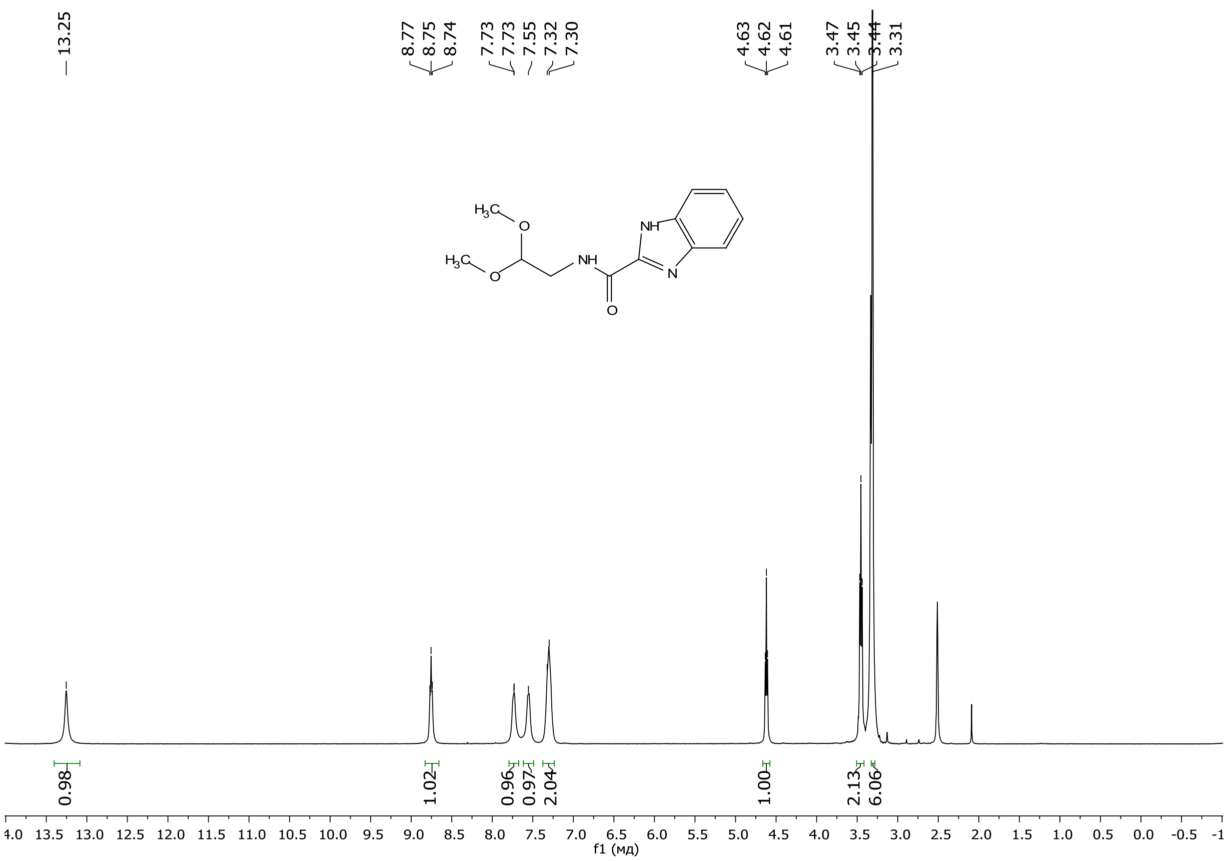

Figure S57. ^{1}H NMR (DMSO-d_6, 600 MHz) spectrum of the compound **10a**

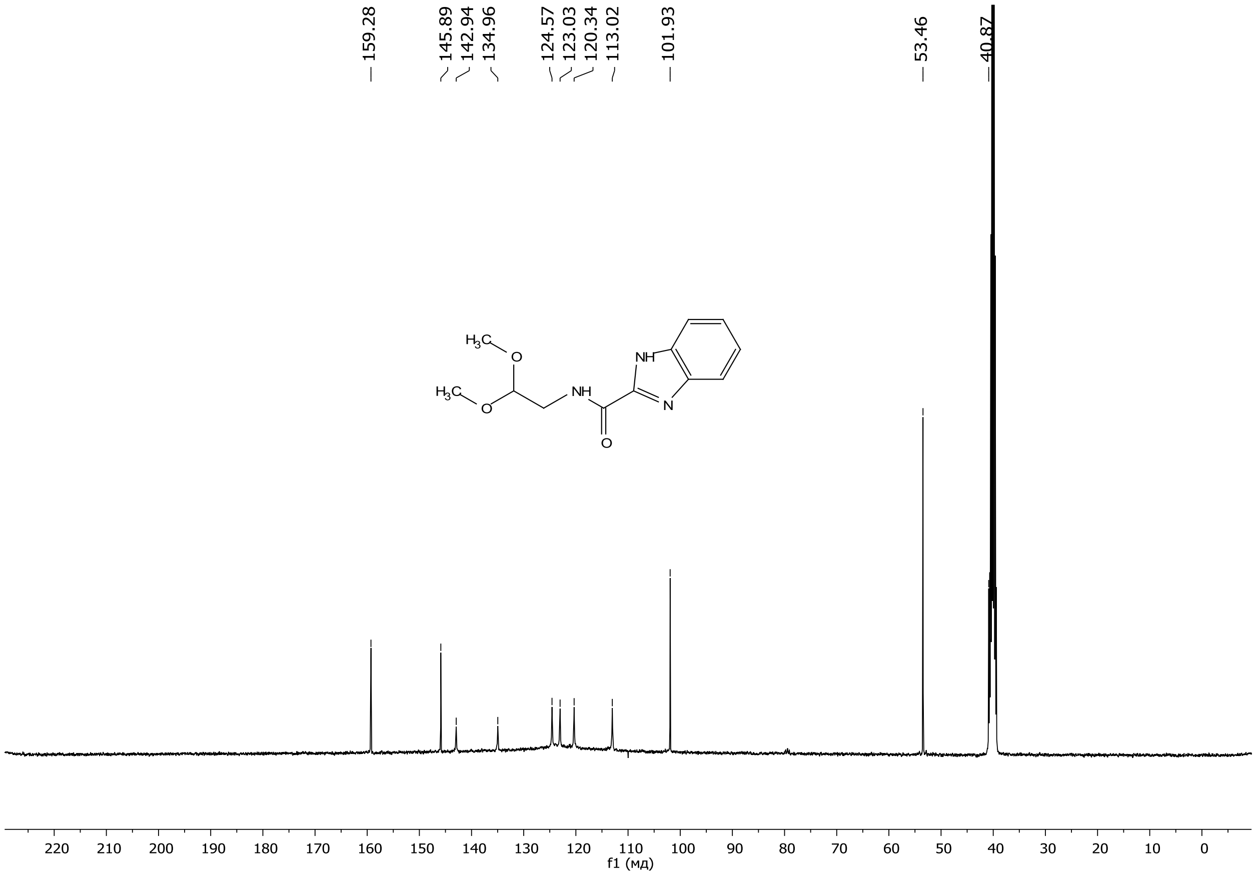

Figure S58. $^{13}C\{^{1}H\}$ NMR (DMSO-d_6, 150 MHz) spectrum of the compound **10a**

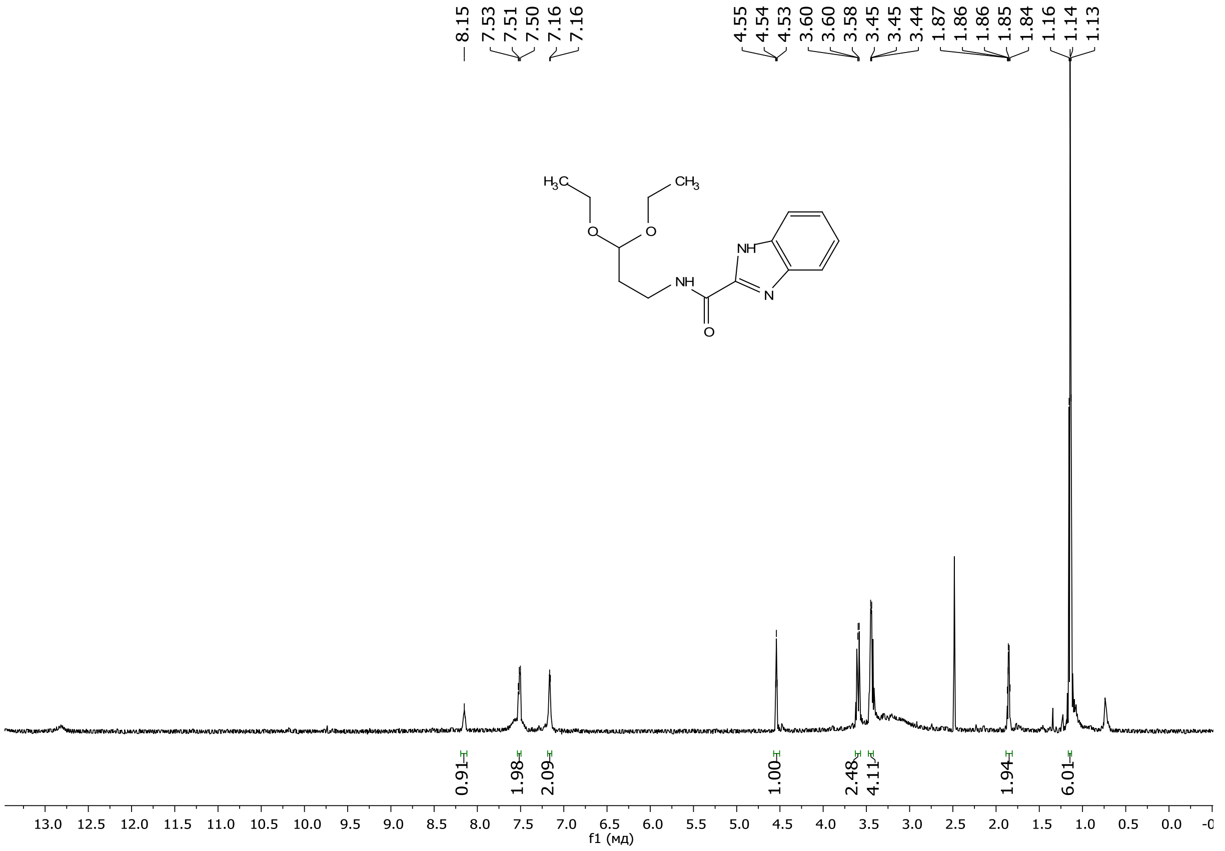

Figure S59. ^{1}H NMR (DMSO-d_6/$CDCl_3$, 600 MHz) spectrum of the compound **10b**

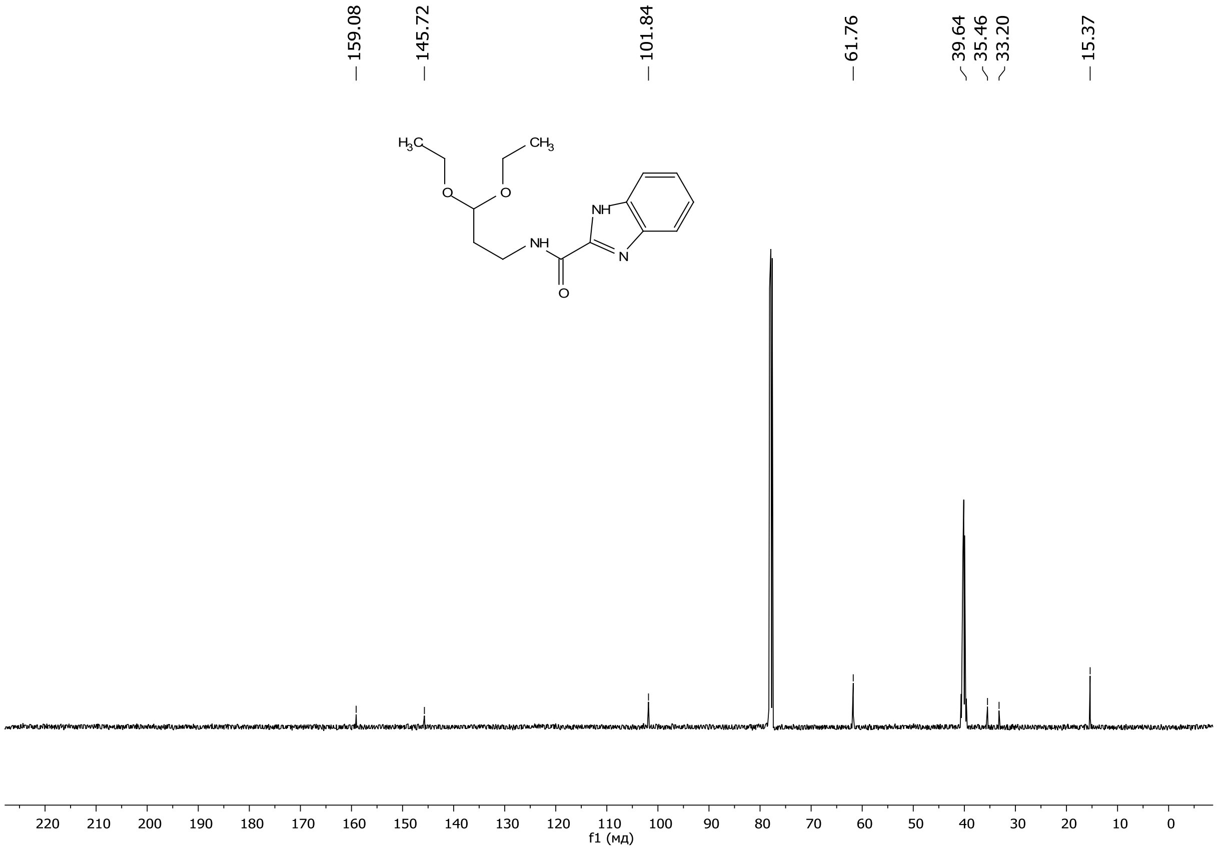

Figure S60. $^{13}C\{^{1}H\}$ NMR (DMSO-d_6/$CDCl_3$, 150 MHz) spectrum of the compound **10b**

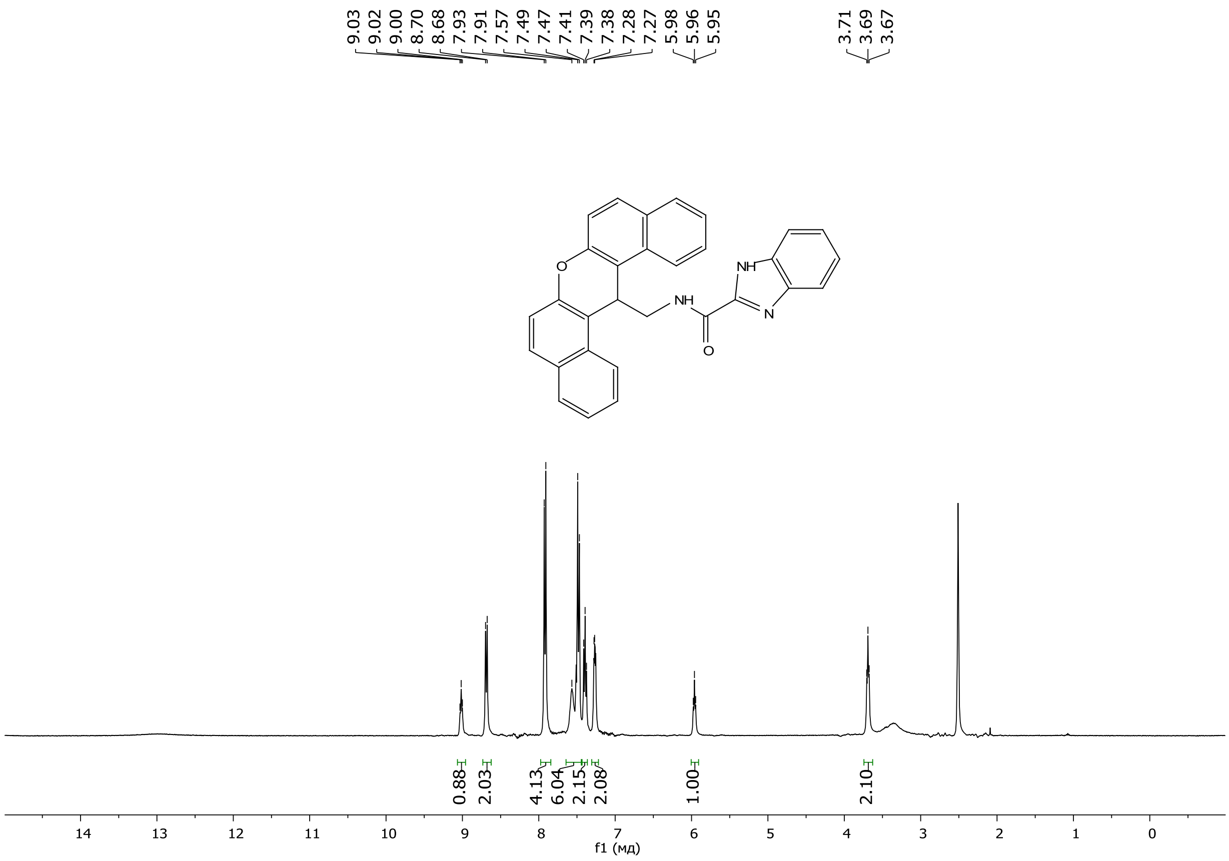

Figure S61. ^{1}H NMR (DMSO-d_6, 600 MHz) spectrum of the compound **11a**

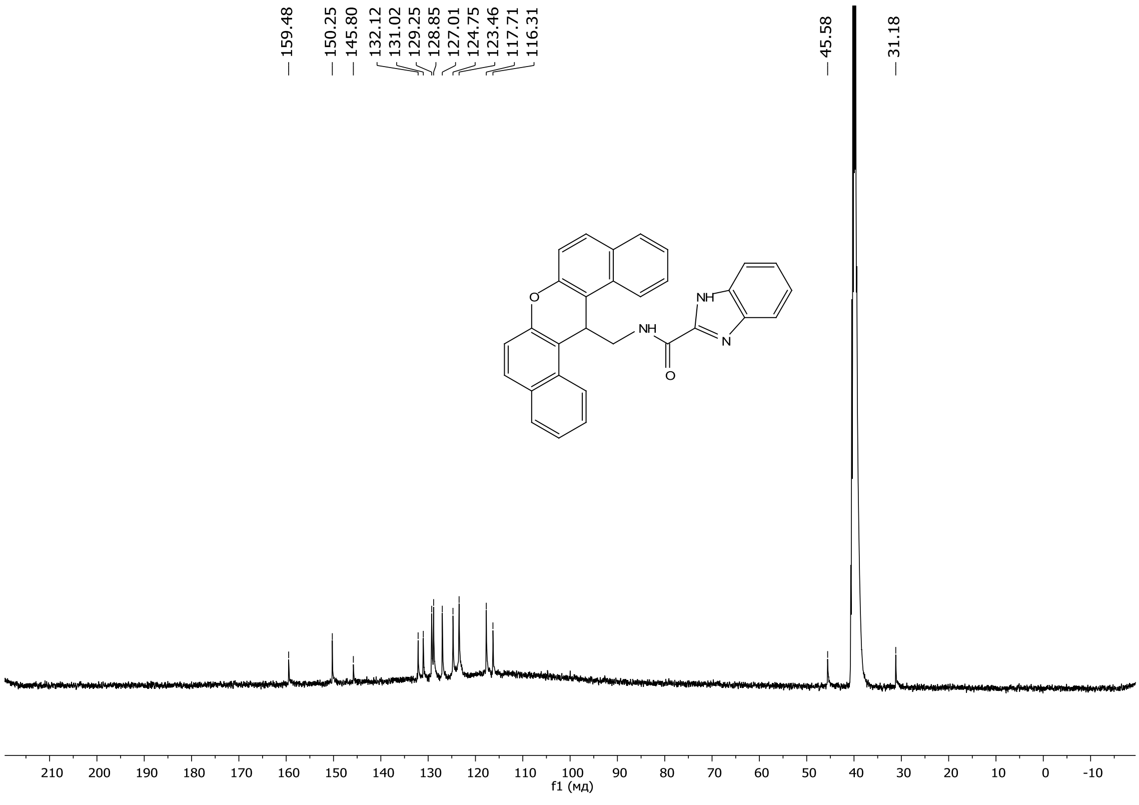

Figure S62. $^{13}C\{^1H\}$ NMR (DMSO-d_6, 150 MHz) spectrum of the compound **11a**

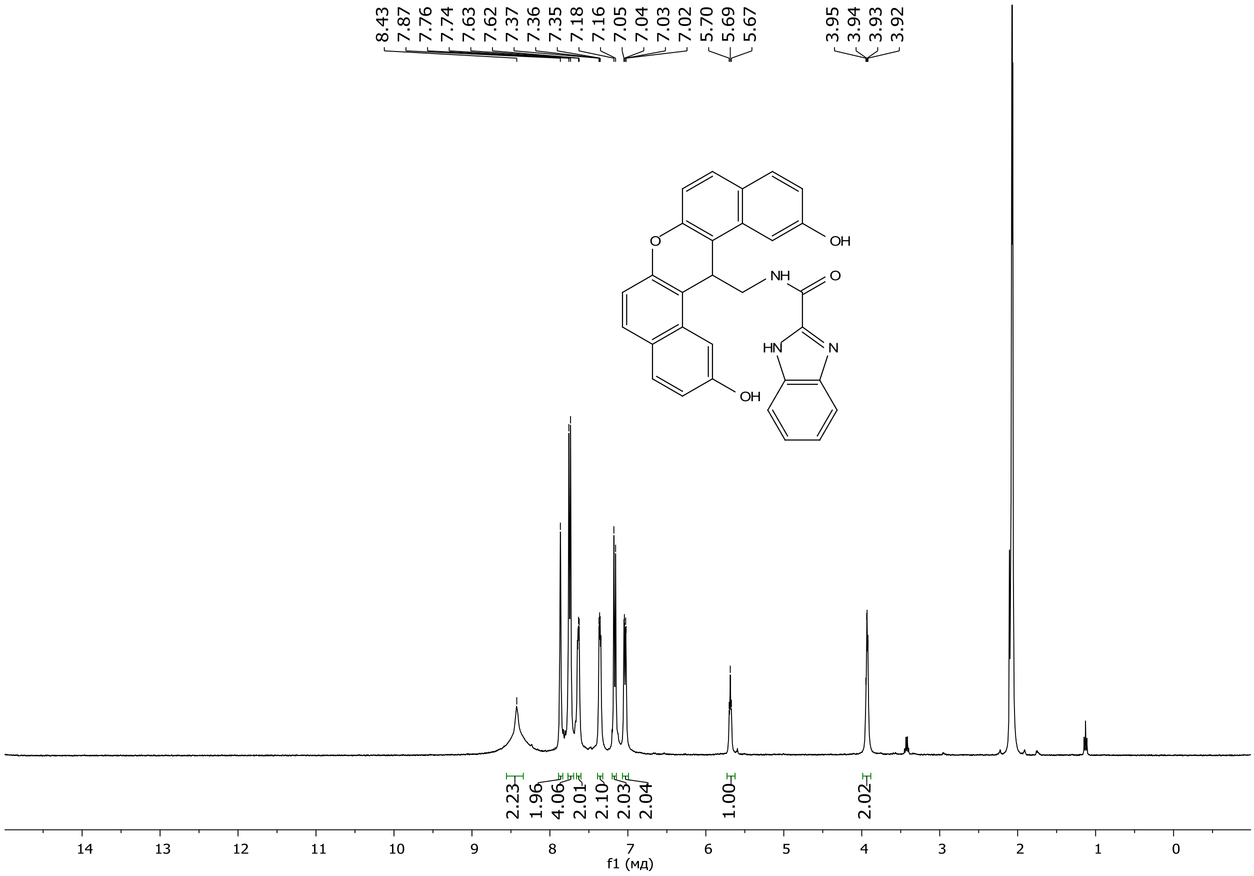

Figure S63. ^{1}H NMR (acetone-d_6, 600 MHz) spectrum of the compound **11b**

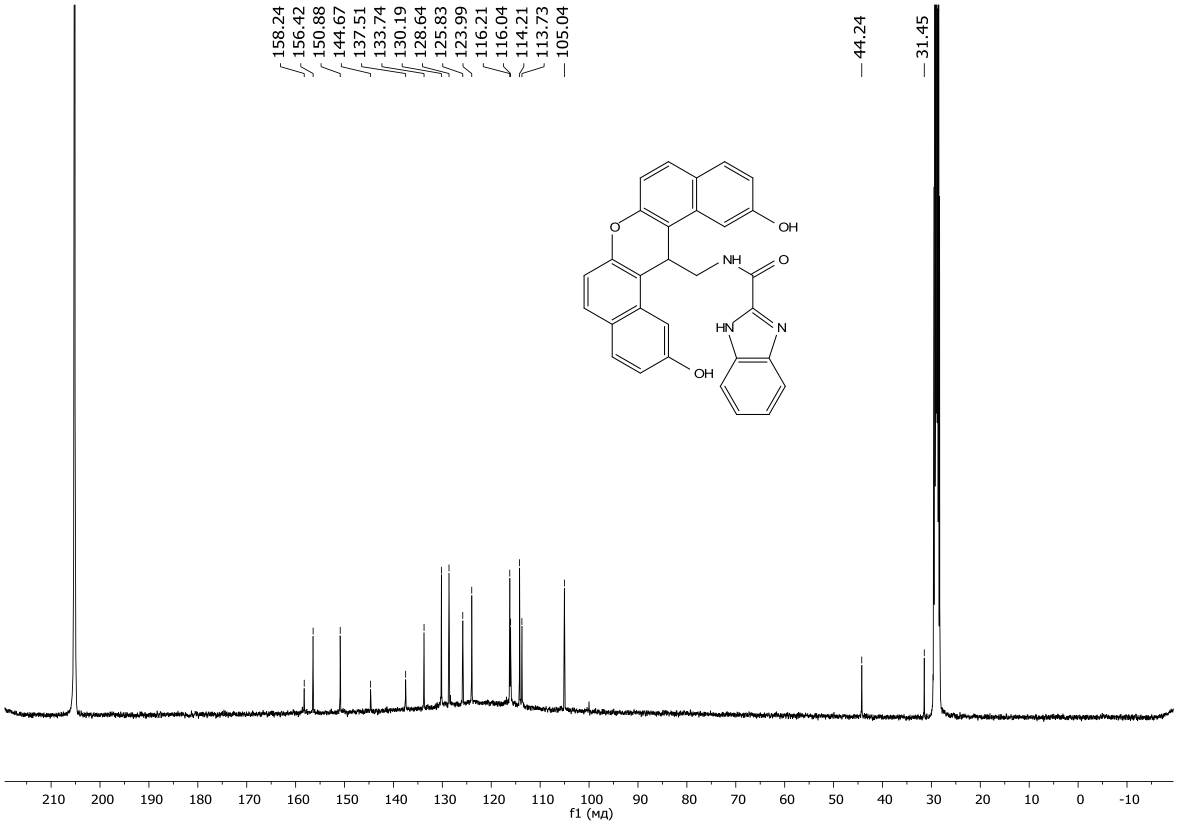

Figure S64. $^{13}C\{^{1}H\}$ NMR (acetone-d_6, 150 MHz) spectrum of the compound **11b**

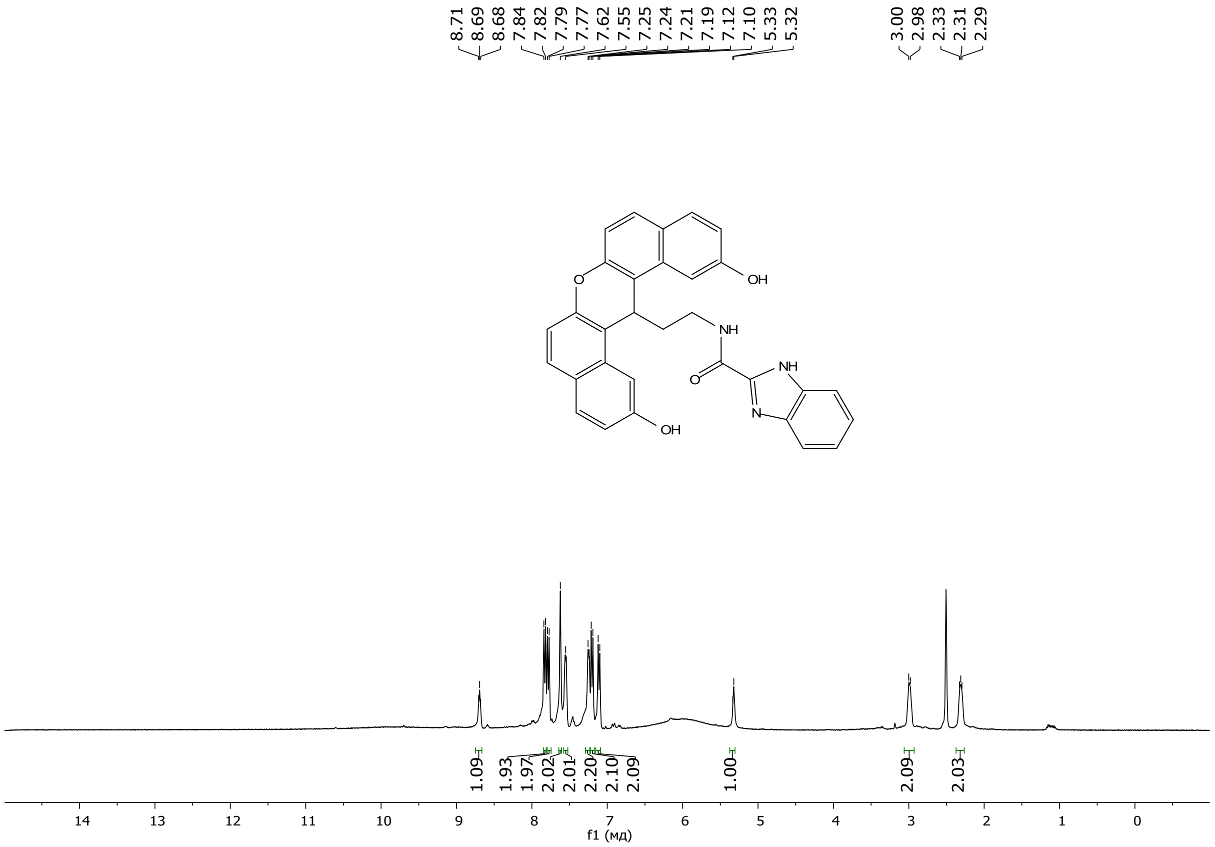

Figure S65. ^{1}H NMR (DMSO-d_6, 600 MHz) spectrum of the compound **11c**

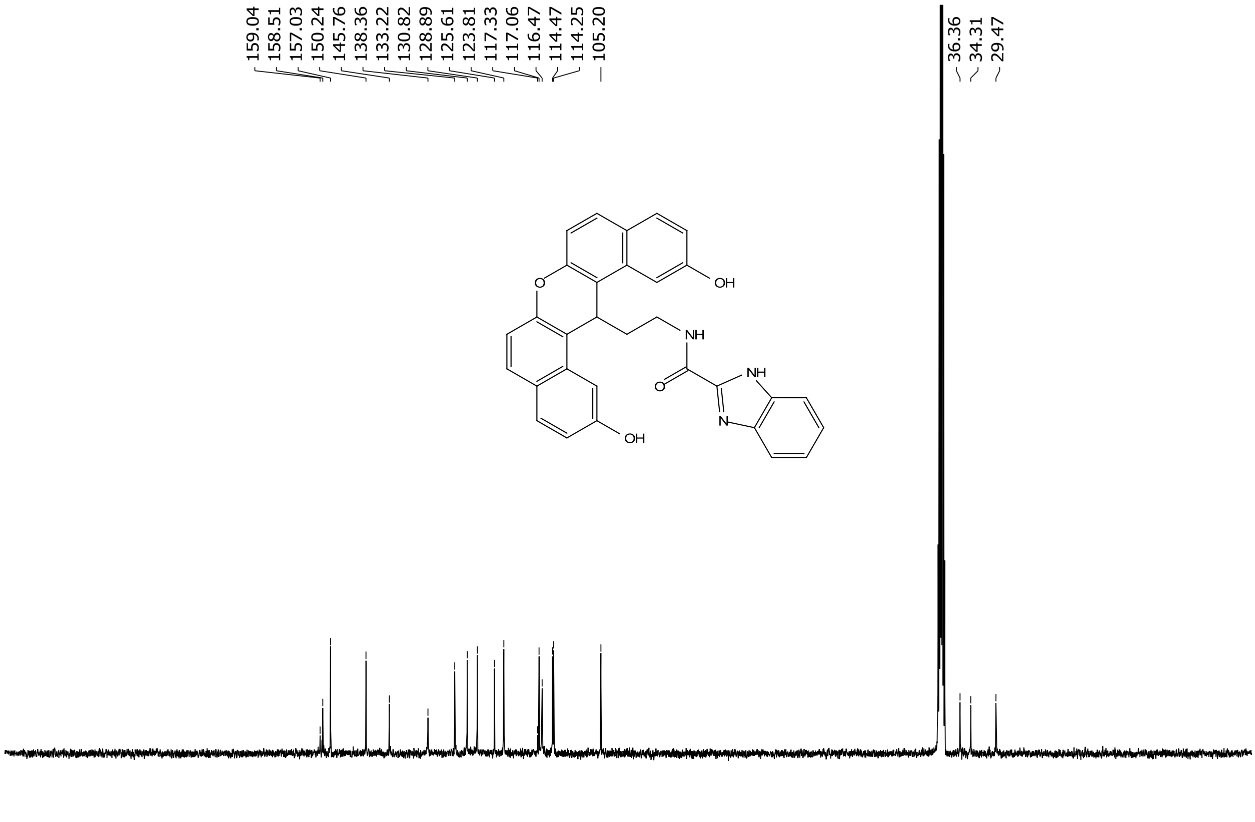

Figure S66. $^{13}C\{^{1}H\}$ NMR (DMSO-d_6, 150 MHz) spectrum of the compound **11c**

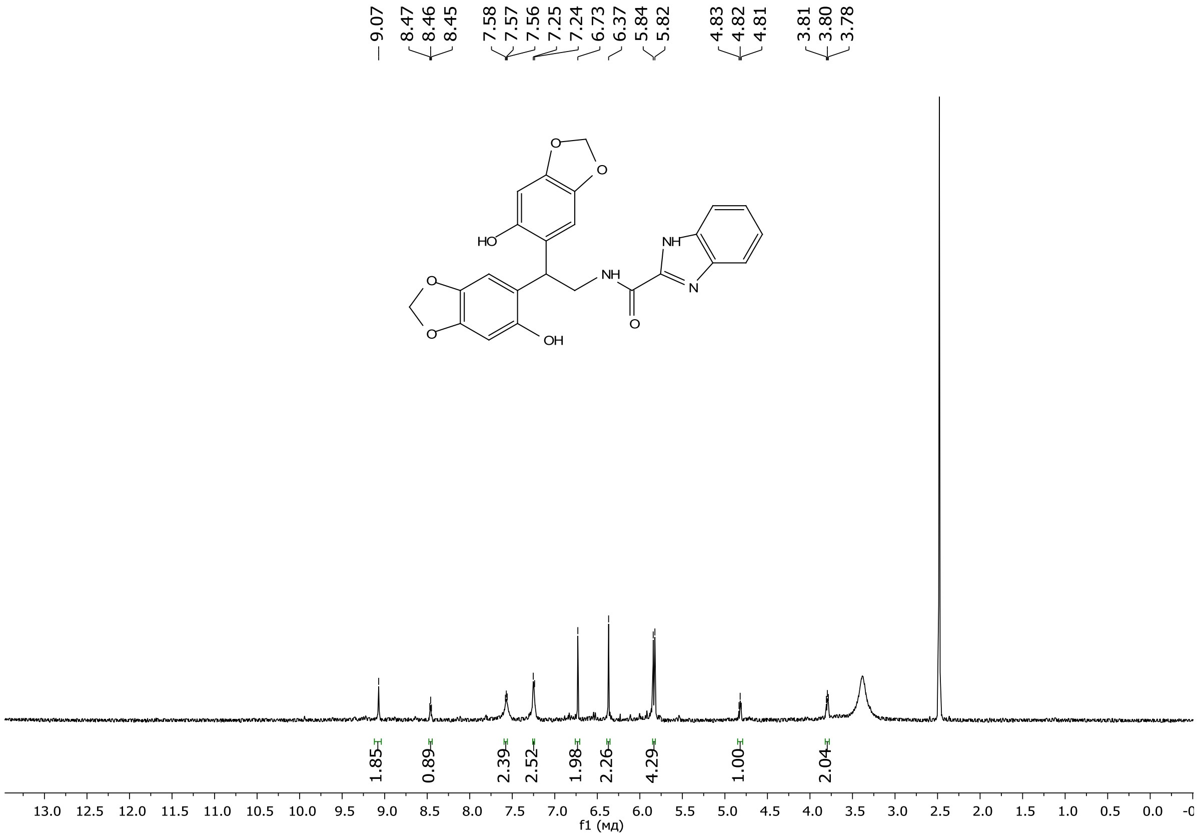

Figure S67. ^{1}H NMR (DMSO-d_6, 600 MHz) spectrum of the compound **12a**

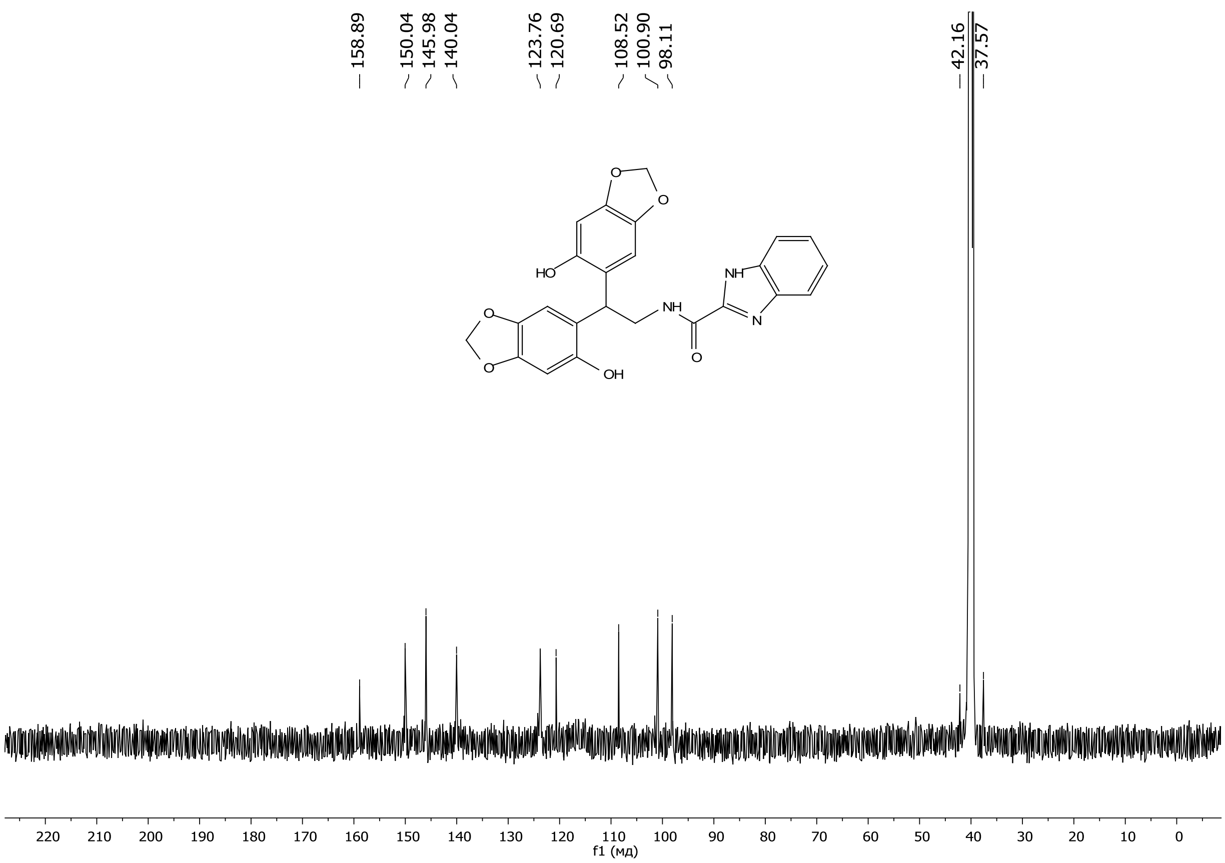

Figure S68. $^{13}C\{^{1}H\}$ NMR (DMSO-d_6, 150 MHz) spectrum of the compound **12a**

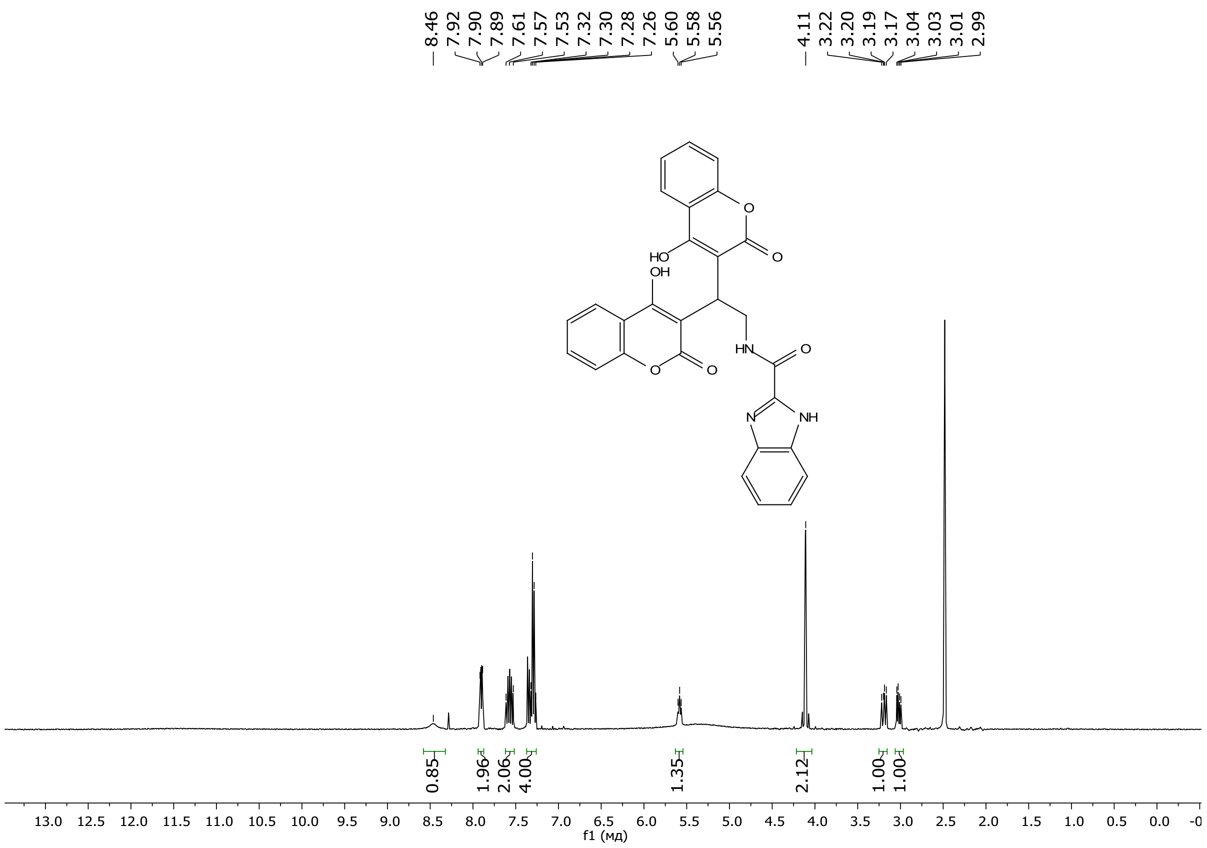

Figure S69. ^{1}H NMR (DMSO-d_6, 600 MHz) spectrum of the compound **12b**

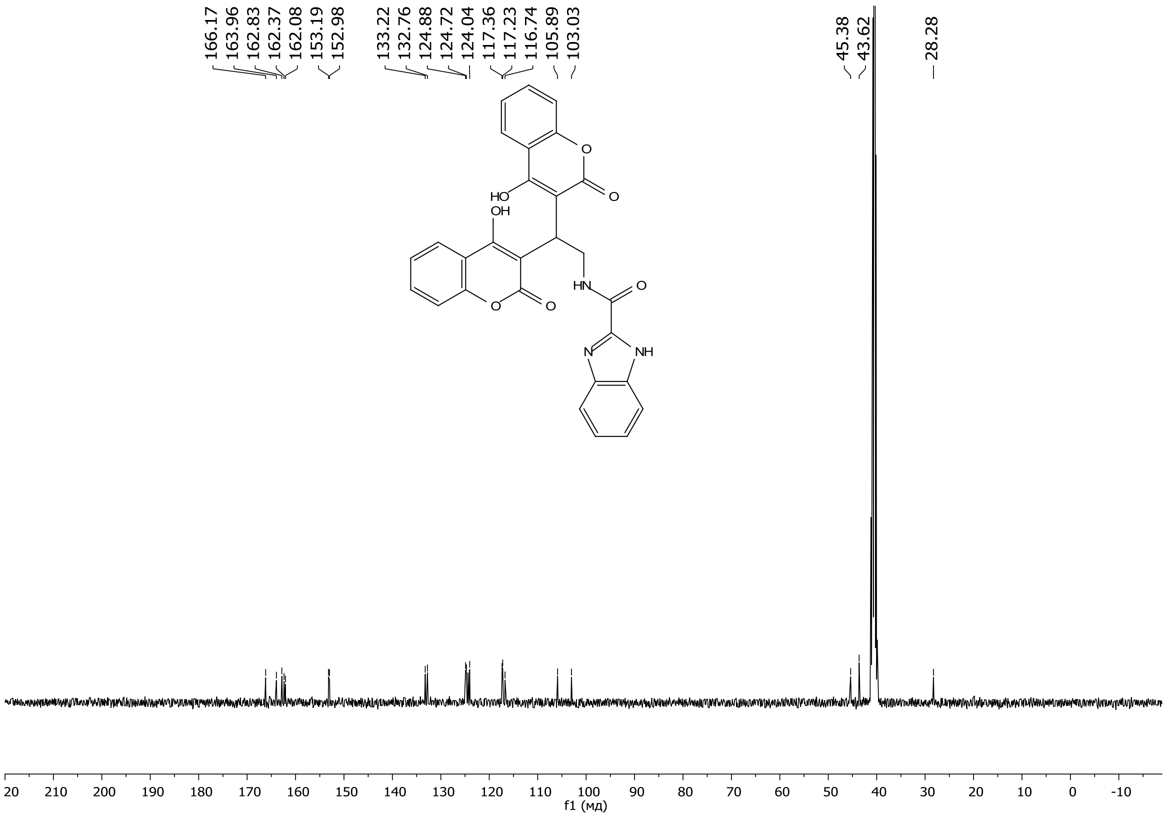

Figure S70. ^{13}C{^{1}H} NMR (DMSO-d_6, 150 MHz) spectrum of the compound **12b**

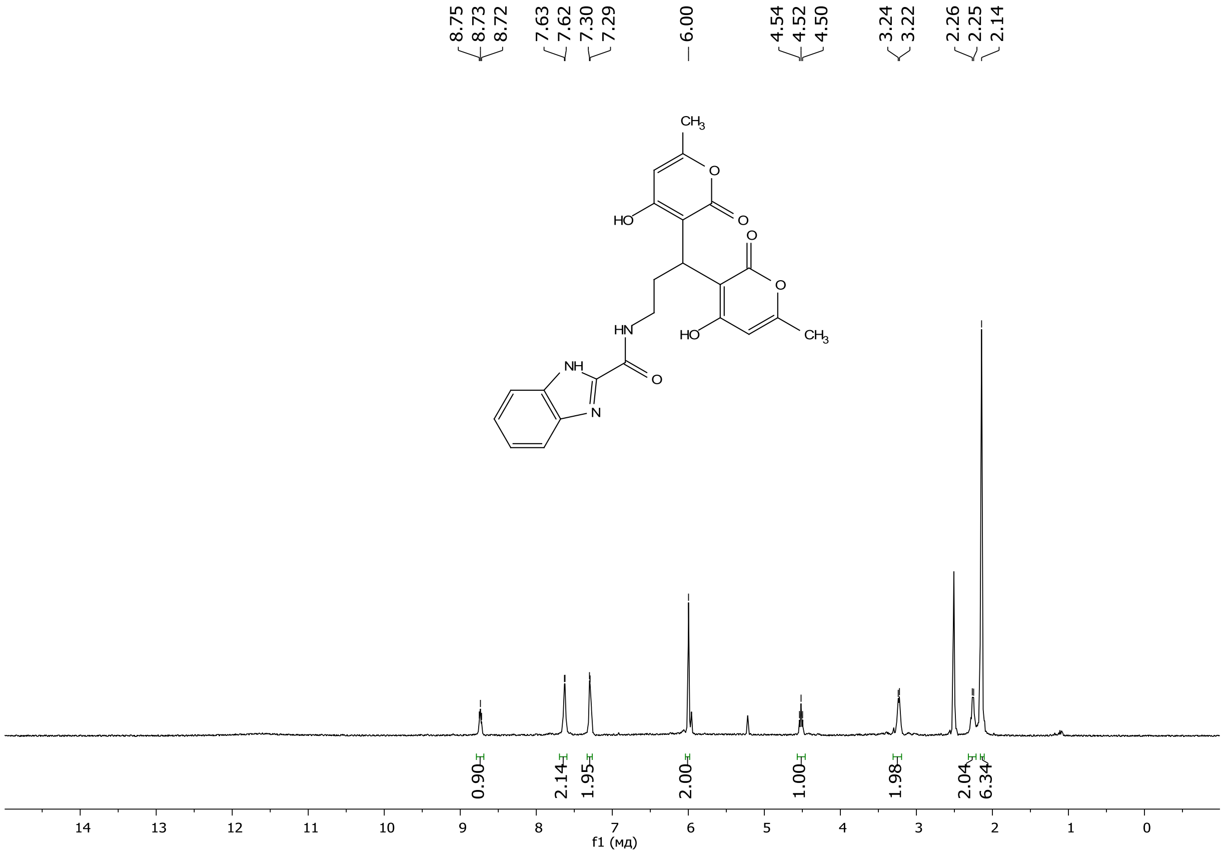

Figure S71. ^{1}H NMR (DMSO-d_6, 600 MHz) spectrum of the compound **12c**

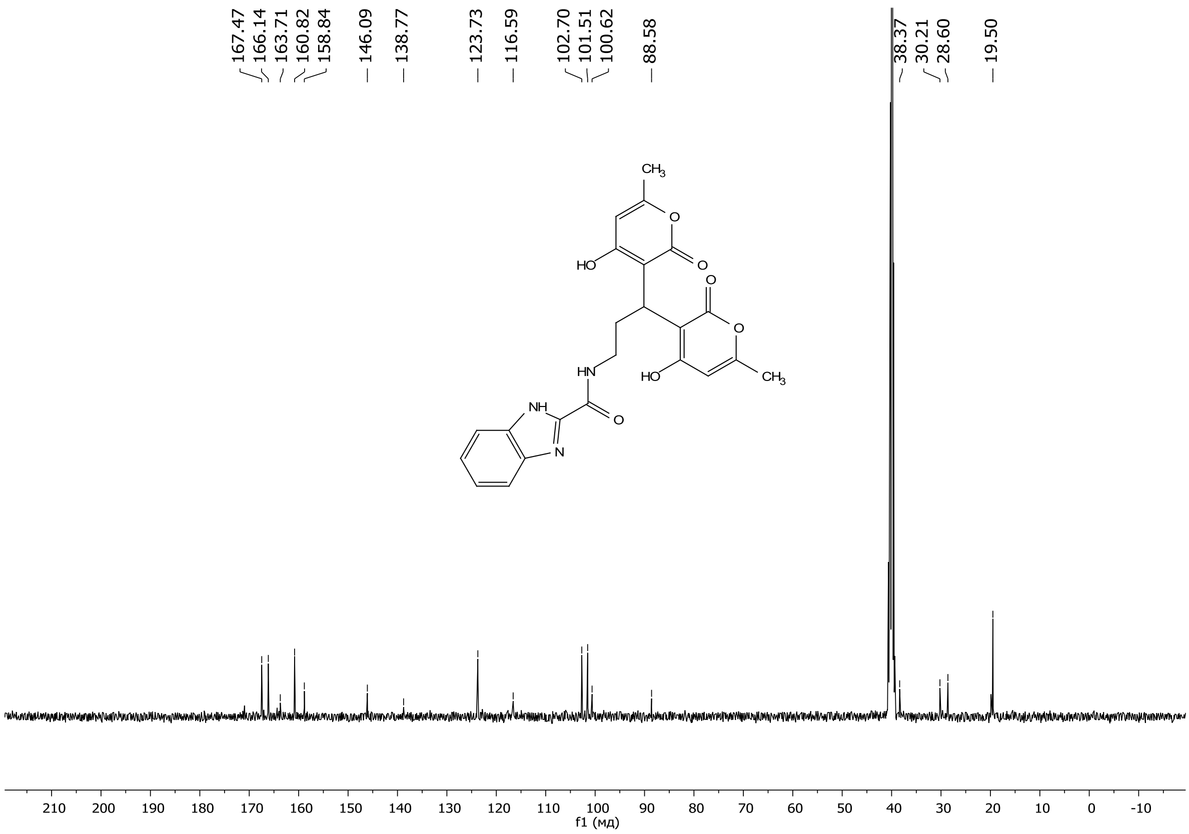

Figure S72. ^{13}C{^{1}H} NMR (DMSO-d_6, 150 MHz) spectrum of the compound **12c**

HRMS for the compounds 5a, 6a,c

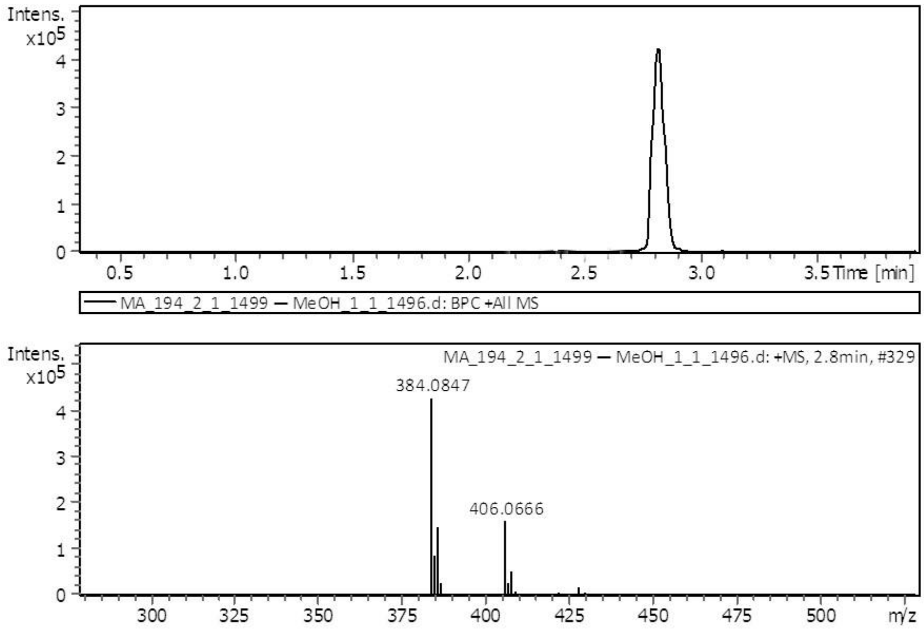

Figure S73. HPLC traces of compound **5a** and MS mass spectra

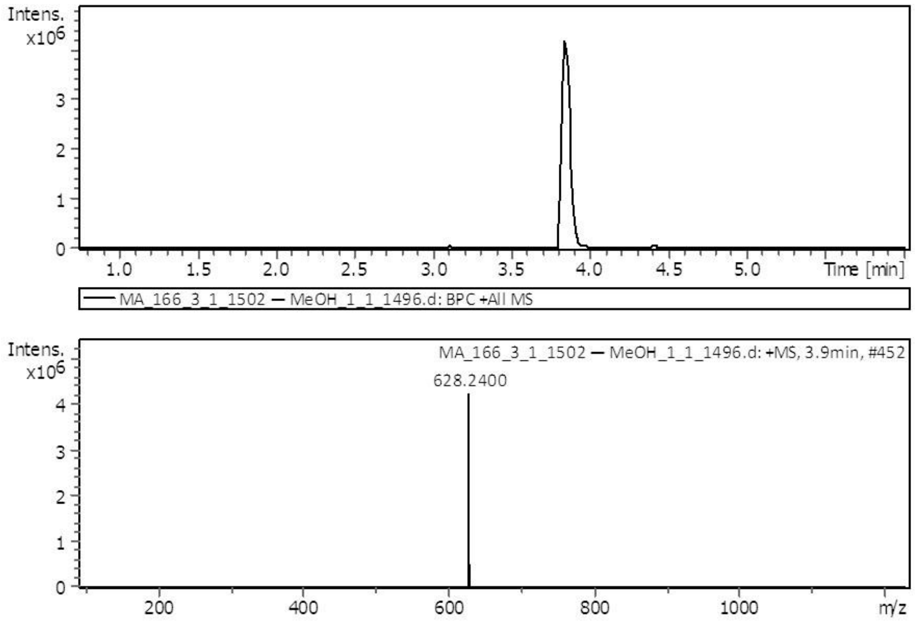

Figure S74. HPLC traces of compound **6a** and MS mass spectra

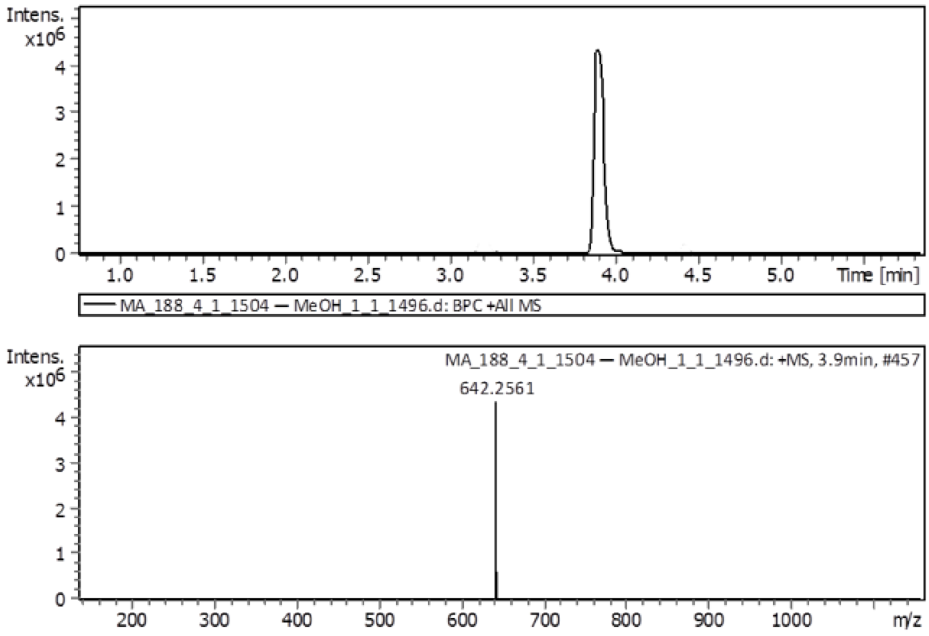

Figure S75. HPLC traces of compound **6c** and MS mass spectra
